# Quantitative Comparison of Avian and Mammalian Physiologies for Parameterization of Physiologically Based Kinetic Models

**DOI:** 10.3389/fphys.2022.858386

**Published:** 2022-04-05

**Authors:** Colin G. Scanes, Johannes Witt, Markus Ebeling, Stephan Schaller, Vanessa Baier, Audrey J. Bone, Thomas G. Preuss, David Heckmann

**Affiliations:** ^1^Department of Poultry Science, University of Arkansas, Fayetteville, AR, United States; ^2^Department of Biological Science, University of Wisconsin–Milwaukee, Milwaukee, WI, United States; ^3^Bayer CropScience AG, Monheim am Rhein, Germany; ^4^esqLABS GmbH, Saterland, Germany; ^5^Bayer Crop Science, Chesterfield, MO, United States

**Keywords:** avian, mammalian, parameterization, physiologically based kinetic (PBK) models, physiologically based toxicological kinetic (PBTK) models

## Abstract

Physiologically based kinetic (PBK) models facilitate chemical risk assessment by predicting *in vivo* exposure while reducing the need for animal testing. PBK models for mammals have seen significant progress, which has yet to be achieved for avian systems. Here, we quantitatively compare physiological, metabolic and anatomical characteristics between birds and mammals, with the aim of facilitating bird PBK model development. For some characteristics, there is considerable complementarity between avian and mammalian species with identical values for the following: blood hemoglobin and hemoglobin concentrations per unit erythrocyte volume together with relative weights of the liver, heart, and lungs. There are also systematic differences for some major characteristics between avian and mammalian species including erythrocyte volume, plasma concentrations of albumin, total protein and triglyceride together with liver cell size and relative weights of the kidney, spleen, and ovary. There are also major differences between characteristics between sexually mature and sexually immature female birds. For example, the relative weights of the ovary and oviduct are greater in sexually mature females compared to immature birds as are the plasma concentrations of triglyceride and vitellogenin. Both these sets of differences reflect the genetic “blue print” inherited from ancestral archosaurs such as the production of large eggs with yolk filled oocytes surrounded by egg white proteins, membranes and a calciferous shell together with adaptions for flight in birds or ancestrally in flightless birds.

## Introduction

Environmental and human risk assessment of xenobiotic substances currently requires a high number of *in vivo* animal tests in multiples species. The use of *in silico* methods to support risk assessment has been promoted because of ongoing efforts to reduce animal testing (Lilienblum et al., 2008; European Chemicals Agency [ECHA], 2011). One example for such *in silico* tools are physiologically based kinetic (PBK) models, previously referred to as physiologically based toxicokinetic (PBTK) models. This class of models aims to describe the kinetics of xenobiotics using a set of physiological compartments that interact mechanistically to describe the absorption, distribution, metabolism and excretion (ADME) of a substance. Within PBK models, physiology of the species and compound-specific properties of the ADME process are treated separately. PBK models can be used for risk assessment applications like dose extrapolation, exposure scenario extrapolation, or interspecies extrapolation (see e.g., Organisation for Economic Co-operation and Development [OECD], 2017; Paini et al., 2019).

Due to applications in pharmacology, significant progress has been made for PBK models that describe mammal species. For example, models for mouse, rat and of course human have been developed and applied successfully (e.g., Mavroudis et al., 2018). Even though birds are an important group in environmental risk assessment (e.g., for pesticides), development of integrated PBK models is not as advanced as for mammals. PBK models are available for the chicken (Yang et al., 2014, 2015; Zeng et al., 2019; Lautz et al., 2020) with PBK models which have been developed in chickens for specific chemicals including ampicillin (Zurich et al., 1984), dioxin (Van Eijkeren et al., 2006), ivermectin (Moreno et al., 2015), midazolam (Cortright et al., 2009), and polychlorinated biphenyls (Van Eijkeren et al., 2006). Substance-specific models have also been developed for the American kestrel methylmercury (Nichols et al., 2010) and hexabromocyclododecane in American kestrels (Fernie et al., 2011) together with turkey, pheasant, and quail (Cortright et al., 2009). Nevertheless, models for many bird species that are relevant in environmental risk assessment are still lacking. Ideally, PBK models should allow determination of the kinetics of all chemical agents for which physico-chemical characteristics are known.

Parameterization of PBK models can be challenging—especially in the case of focal or endangered species—and frequently requires extrapolation of parameters between species. A systematic comparison between the relevant physiological, anatomical, metabolic, and allometric parameters between birds and mammals would help with the parameterization and species extrapolation that is required to construct bird PBK models.

Birds and mammals share many physiological, metabolic and anatomical characteristics. The divergence of the two groups is estimated to have occurred about 315 million years ago in the Late Carboniferous period (313 million years ago: Benton and Donoghue, 2006; 318 million years ago: Ford and Benson, 2020), thus explaining some similar characteristics. Others are due to convergent evolution (Wu and Wang, 2019). Examples of the former include the presence of liver, lungs and kidneys and the latter of endothermy, high blood pressure and a four chambered heart (Brusatte et al., 2014, 2015; Wu and Wang, 2019). In addition, there are series of characteristics where there are major qualitative differences reflecting an overarching blueprint (Brusatte et al., 2014, 2015). These include the presence of erythrocytes with nuclei in birds together with the production of large yolky eggs. It is questioned whether adaptations for flight impact these characteristics. Rather they reflect the importance of successful reproduction and/or the evolutionary history of the taxa. However, the presence of feathers is critical to flight (reviewed e.g., Brusatte et al., 2014, 2015), important to thermoregulation and represents a morphological novelty in the a*rchosaurs* (Xu et al., 2014). The present paper quantitatively compares physiological, metabolic and anatomical characteristics of avian and mammalian species. An understanding of the quantitative magnitude of these characteristics is required to decide whether transfer of quantitative PBK models between the two groups is feasible for the physiological process in question.

In homothermic vertebrates, allometric relationships exist not only for metabolic rate but also for organ weights [e.g., negative for heart mass in birds: Grubb (1983) and African mammals (reviewed: Smith, 1984) and positive for skeletal mass in mammals and birds: Prange et al. (1979)], development (Cooney et al., 2020), pharmacokinetics (Knibbe et al., 2005) and calculating drug dosages for laboratory animals (Lindstedt and Schaeffer, 2002). In addition, allometric relationships have been reported in invertebrate species; there being, for instance, such relationships for size or weight of the stomach (Griffen et al., 2018) and claws (Vermeiren et al., 2020) in crabs and multiple organs in coleopteran and hymenopteran insects (Polilova and Makarova, 2017).

The present communication provides an analysis of these characteristics (including allometric relationships) between avian and mammalian species and between sexually mature versus immature female birds. These differences are of both evolutionary importance and may also be critical in the development of physiologically based kinetic (PBK) avian models. Some, but arguable not all, physiological, metabolic and anatomic characteristics are also important in the development of sound PBK models for birds.

## Materials and Methods

### Development of Data Bases for Physiological, Biochemical, and Morphological Characteristics

Data bases were developed based on published reports. Among the key search terms employed were the following:

Hematocrit AND mammals (or birds) AND scholarly

Hemoglobin AND mammals (or birds) AND scholarly

Erythrocyte count AND mammals (or birds) AND scholarly

Mean cell volume AND mammals (or birds) AND scholarly

Plasma AND protein (or albumin or lipid or triglyceride or phospholipid or cholesterol or immunoglobulin) AND concentrations AND mammals (or birds) AND scholarly

Ovary (or uterus/oviduct or lungs or kidneys or heart or other organs) AND weight AND mammals (or birds) AND scholarly

Liver (together other organs) AND mammals (or birds) AND cellularity (or lipids) scholarly

It is noted that many of the first four parameters were reported in the supplements to Scanes (2015).

This was followed by replacing the term “mammals” or “birds” with each of the following: major domestic animals (mammals: cats, cattle, dogs, ferrets, goats, pigs, sheep; birds: chickens, ducks, geese, Muscovy ducks, ostriches, pheasants, and guinea fowl), model species (rats, mice, monkeys, hedgehogs, mallards, bobwhite quail, and Japanese quail) and much studied wild species (white-tailed deer, dolphins). In addition, daughter references were employed. The objective of the search was to accumulate a compendium of data from as many avian species as possible.

These data bases with references to the cited publications are available in the supplements.

### Choice of Avian Species and Their Ages

The analyses did not factor in phylogenic considerations. It is recognized that for some physiological and anatomical parameters, the available data in the literature are from domesticated species and two avian orders (Anseriformes and Galliformes). This was not viewed as a problem as PBK models are most important for an anseriform bird, mallards, together with galliform birds, e.g., laying hens and bobwhite quail.

Sexually mature female birds were those that were laying eggs either in domesticated species or wild birds during the breeding season. Sexually immature birds included in the analysis were included in the analysis when birds were over 75% of mature body weight and were not laying eggs or incubating eggs.

### Statistical Analysis

Comparisons between characteristics in avian and mammalian species were analyzed by the unpaired student’s *t*-test; there being no evidence that the data were not normally distributed. In contrast, comparisons between sexually mature female birds and sexually immature/male birds were analyzed by paired *t*-tests where data was available from the same reports for sexually mature female birds and sexually immature/male birds. Moreover, linear regression was employed to analyze allometric relationships between relative organ weight (organ weight as a percentage of body weight) and log_10_ body weight.

## Results

Tables 1–3 summarize, the mean ± (*n* = species) SEM of the following: (1) blood parameters in avian and mammalian species, (2) blood parameters and reproductive organs in sexually immature female/male with sexually mature female birds, and (3) Liver parameters together with relative weights of organs.

**TABLE 1:** Blood parameters in avian and mammalian species.

Parameters	Mean ± (n = number of species) SEM	
BLOOD	Mammals^A^	Birds^B^
**ERYTHROCYTES**		
Hematocrit/packed cell volume %	42.0 ± (209) 0.48	44.5 ± (349) 0.31***
Hemoglobin g dL^–1^	15.0 ± (209) 0.21	15.1 ± (262) 0.18
Erythrocyte # × 10^6^ μL^–1^	7.13 ± (209) 0.18	2.97 ± (262) 0.061***
Mean cell (erythrocyte) volume (MCV) fL	71.9 ± (209) 2.25	162.0 ± (259) 2.50***
Mean cell hemoglobin (MCH pg)	24.3 ± (210) 0.87	59.3 ± (102) 0.87***
Mean cell (erythrocyte) hemoglobin concentrations (MCHC) fg fL^–1^	337 ± (186) 3.5	337 ± (102) 4.4

**BLOOD PLASMA**	**Mammals^C^**	**Birds^C^**

Total lipid g dL^–1^	0.359 ± (18) 0.035	0.623 ± (3) 0.105**
Triglyceride g dL^–1^	0.057 ± (25) 0.015	0.237 ± (16) 0.043***
Phospholipid g dL^–1^	0.136 ± (21) 0.0181	0.295 ± (6) 0.105
Cholesterol g dL^–1^	0.149 ± (28) 0.0127	0.227 ± (9) 0.030**
Protein g dL^–1^	6.59 ± (41) 0.14^A^	3.83 ± (195) 0.052***
Albumen g dL^–1^	3.59 ± (39) 0.09^A^	1.57 ± (150) 0.033***

Difference **P < 0.01; ***P < 0.001.

*^A^Calculated from data on individual species is available in or calculated from Supplementary Table 1.*

*^B^Based on supplementary data in Scanes (2022). It is assumed that these data do not include sexually mature (laying) female birds.*

*^C^Calculated from data on individual avian and mammalian species available in from Supplementary Tables 2, 3.*

**TABLE 2:** Parameters in laying and non-laying birds.

Parameter	Mean ± (*n* = number of species) SEM	
	Sexually immature females^A^	Sexually mature female birds (laying)^A^
BLOOD		
Hematocrit/PCV%	48.5 ± (12) 2.11	42.1 ± (12) 1.97***
Hemoglobin g dL^–1^	13.4 ± (7) 1.05	12.6 ± (7) 0.96
Plasma triglyceride g dL^–1^	0.237 ± (16) 0.043	1.840 ± (4) 0.150***
Plasma phospholipids g dL^–1^	0.295 ± (6) 0.105	0.381 ± (3) 0.018
Plasma cholesterol g dL^–1^	0.227 ± (9) 0.890	0.178 ± (3) 0.003

**REPRODUCTIVE ORGANS**	**Sexually immature females^B^**	**Sexually mature female birds (laying)^B^**

Ovary weight g%	0.116 ± (11) 0.0478	1.627 ± (15) 0.298***
Oviduct weight g%	0.318 ± (9) 0.134	3.202 ± (12) 0.800**

** Difference P < 0.01, *** P < 0.001.

*^A^Calculated from data on individual avian and mammalian species available in Supplementary Table 4.*

*^B^Calculated from data on individual avian and mammalian species available in from Supplementary Table 5.*

**TABLE 3:** Comparison of the relative weights of major organs (as a percentage of body weight) between avian and mammalian species.

	Mean ± (*n* = number of species) SEM	
	Birds (Sexually immature females/males)	Mammals
**LIVER**		
**Relative weight** g 100 g^–1AB^	2.839 ± (112) 0.0906	2.846 ± (90) 0.151
**Cellularity**		
DNA mg g^–1C^	3.26 ± (4)1.20	2.02 ± (9) 0.40
Liver cell number × 10^9^ per g^C^	1.58 ± (4) 0.43	0.354 ± (9) 0.073**
Genome size pg^C^	1.93 ± (4) 0.31***	5.88 ± (9) 0.24**
Liver cell volume pL^D^	0.81 (4) 0.22	5.54 ± (9) 1.97
**Composition**		
Total lipid g 100 g^–1EF^	5.40 ± (8) 0.83	3.89 ± (15) 0.18*
Triglyceride g 100 g lipids^–1GH^	24.8 ± (7) 5.9	25.4 ± (4) 7.0
Phospholipids g 100 g lipids^–1GH^	61.7 ± (7) 7.2	64.6 ± (4) 7.3
Cholesterol g 100 g lipids^–1GH^	10.8 ± (7) 4.2	10.1 ± (4) 2.0
**RELATIVE WEIGHTS g 100g^–1^**		
Brain^AI^	2.266 ± (95) 0.159	1.153 ± (117) 0.118***
Heart^AI^	1.184 ± (112) 0.0455	0.643 ± (93) 0.03***
Kidney^AI^	1.148 ± (96) 0.046	0.766 ± (206) 0.034***
Lungs^AI^	1.526 ± (45) 0.110	1.436 ± (91) 0.079
Ovary^J^	0.116 ± (11) 0.0478	0.0193 ± (12) 0.00774*
Oviduct/non-gravid uterus^J^	0.318 ± (9) 0.134	0.103 ± (5) 0.0360
Small intestine plus proventriculus/gizzard (birds) or stomach (mammals)^AK^	6.774 ± (63) 0.624	9.799 ± (45) 1.080*
Spleen^AI^	0.134 ± (21) 0.050	0.331 ± (53) 0.039**
**RELATIVE LENGTH**		
Small intestine cm kg^–1L^	395 ± (61) 49.7	275 ± (21) 105
**VILLUS HEIGHT/LENGTH μm**		
Duodenum^M^	967 ± (4) 188.0	574 ± (9) 61.7^R^
Jejunum^M^	701 + (4) 143.3	529 + (10) 58.4
Ileum^M^	535 ± (4) 108.5	370.2 ± (9) 53.7
**ORGAN FUNCTIONING**	Mean ± (*n* = number of species) SEM in mL min^–1^ kg^–1^	
Glomerular filtration rate (GFR^)N^	4.50 ± (35) 0.61	2.82 ± (18) 0.46^†×^

Different *P < 0.05; **P < 0.01; ***P < 0.001 from birds.

*^†^P < 0.1.*

*^A^Calculated from data on individual avian species in Supplementary Table 7.*

*^B^Calculated from data on individual mammalian species in Supplementary Table 8.*

*^C^Determined from liver DNA concentration and diploid genome mass based on data in Supplementary Table 9.*

*^D^Estimated from the liver cell number per gram.*

*^E^Birds: Patel et al., 1977; Desmeth, 1981; Hermier et al., 2003; Wan et al., 2018.*

*^F^Mammals: Ralli et al., 1941; Kwiterovich et al., 1970; Neill et al., 1977; Christie, 1985; Hall et al., 1997; Wang et al., 2000; Cable et al., 2009; Fernando et al., 2012; Tajik et al., 2012; Siques et al., 2014; U.S. Department of Agriculture, Agricultural Research Service [USDA ARS], 2020.*

*^G^Neill et al., 1977; Patel et al., 1977; Desmeth, 1981; Wang et al., 2000; Hermier et al., 2003; Wan et al., 2018; U.S. Department of Agriculture, Agricultural Research Service [USDA ARS], 2020.*

*^H^Ralli et al., 1941; Kwiterovich et al., 1970; Christie, 1985; Hall et al., 1997; Helbig, 2006; Cable et al., 2009; Siques et al., 2014; U.S. Department of Agriculture, Agricultural Research Service [USDA ARS], 2020.*

*^I^Calculated from data on individual mammalian species in Supplementary Table 8.*

*^J^Calculated from data on individual species in Supplementary Tables 5 (birds), 6 (mammals).*

*^K^Calculated from data on individual mammalian species in Supplementary Table 10.*

*^L^Calculated from data on individual mammalian species in Supplementary Tables 12, 13.*

*^M^Calculated from data on individual mammalian and avian species in Supplementary Tables 14 (mammals), 15 (birds).*

*^N^Calculated from data on individual mammalian and avian species in Supplementary Table 15.*

### Blood

#### Hematocrit/Packed Cell Volume

The hematocrit (Hct) or packed cell volume (PCV) were 6.0% higher (*P* < 0.001) in birds than mammals (Table 1). The Hct/PCV was lower (13.2%) (*P* < 0.001) in sexually mature female birds than in sexually mature female birds and male birds (Table 2). Parenthetically, it is noted that the Hct/PCV is 13.6% lower (*P* < 0.001) in gallinaceous birds (chickens, quail, turkeys, pheasants, guinea fowl, and related birds) [38.7 ± (18) 1.31%] than other avian species [Hctt/PCV: 44.8 ± (332) 0.32%].

#### Blood Hemoglobin

Both blood hemoglobin concentrations and mean cell (erythrocyte) hemoglobin concentrations (MCHC) in mammals and birds were identical (Table 1). However, blood concentrations of hemoglobin were lower (*P* < 0.05) in sexually mature female birds than in sexually immature plus male birds (by 6.7%) (Table 2). Moreover, blood concentrations of hemoglobin were 15.1% lower (*P* < 0.01) in gallinaceous birds [138.5 ± (16) 9.19 fL] than other avian species [163.4 ± (246) 2.55 fL] (Table 3).

#### Erythrocyte Numbers

There was a marked difference (*P* < 0.001) between blood erythrocyte numbers in mammals and birds (Table 1) with erythrocyte numbers being 60.9% lower in birds than mammals. In turn, the mean cell (erythrocyte) volume (MCV) was 225.3% greater (*P* < 0.001) in avian than mammalian species (Table 1). This may reflect the presence of nuclei, mitochondria, ribosomes and other organelles in avian but not mammalian erythrocytes. MCV was 15.2% smaller (*P* < 0.05) in gallinaceous birds [138.5 ± (16) 9.19] than other avian species [163.4 ± (246) 2.55] (Table 3).

#### Plasma/Serum Concentrations of Protein and Albumen

The mean plasma concentrations of total protein and albumen were markedly lower *(P* < 0.001) in birds than mammals (see Table 1); with avian concentrations being, respectively, 41.9 and 56.3% lower than those in mammals.

#### Plasma/Serum Concentrations of Lipids

There were marked differences in plasma concentrations of total lipid (*P* < 0.01), triglyceride (*P* < 0.001) and cholesterol (*P* < 0.001) between non-laying birds and mammals (Table 1). Plasma concentrations of lipids were higher in birds than mammals with total lipids being 73.1% higher, triglyceride being 3.54 fold high and cholesterol being 58.7% higher. The plasma concentrations of total lipids and triglyceride are greater in sexually mature female birds than immature females/males (Table 2). The increases were, respectively, 3.71 and 7.76 fold greater in sexually mature female birds than immature females/males (Table 2). Similarly, there were higher (*P* < 0.05) concentrations of triglyceride in the plasma of laying hens than prepuberal female chickens [prepubertal female: 0.408 ± (3) 0.063 g dL^–1^; laying hens: 2.268 + (5) 0.516 g dL^–1^]. In contrast, there were not differences (*P* > 0.05) in plasma concentrations of cholesterol or phospholipids between laying and non-laying birds (Table 2).

#### Plasma/Serum Concentrations of Immunoglobulin

Reports of serum/plasma concentrations of IgG (mammals) and IgY (birds) exhibit marked variation with coefficients of variation (>100%) (for details see Supplementary Table 11). In addition, there are relatively few reports from either avian or mammalian species. Under these circumstances, it was considered not possible to make comparisons between mammals and birds.

### Major Organs

#### Organs Weights (Liver, Kidneys, Lungs, Spleen, and Heart)

Both the liver and kidneys are critical to the clearance and metabolism (detoxification) of drugs, pesticides and other chemical agents. There was no difference in the relative weights of the liver between avian and mammalian species (Table 3). Similarly, there were no differences in the relative weights of the lungs (Table 3). In contrast, the relative weights of the kidneys and heart were, respectively, 78.0 and 84.1% greater (*P* < 0.001) in birds than mammals (Table 3). Moreover, the relative weights of the spleen were 59.5% lower in birds than in mammals (Table 3).

#### Organs Weights (Ovary and Oviduct)

Relative ovary weights were greater (*P* < 0.05) in sexually mature birds than in sexually immature mammals (by 6.01 fold) (see Table 2). Avian oviducts tended (*P* = 0.15) to be greater than non-gravid uteri in mammals (see Table 2).

There were marked differences in ovarian weights and oviducts between sexually mature and immature birds (see Table 4). During sexual maturation/seasonal breeding, weights of the ovaries and oviducts are increased (*P* < 0.01) by, respectively, 14.0 and 10.1 fold (see Table 4). Similarly, weights of the ovary and oviduct are increased by, respectively, 52.6 and 61.7 fold in female chickens following photostimulation and consequent induction of sexual maturation in chickens (see Table 5).

**TABLE 4:** Allometric relationships between log_10_ organ weight or physiological function versus log_10_body weight (g) in birds and mammals.

	Birds	Mammals
**Adjusted *R*^2^ [*P* = ]**		
Organ weights g		
Brain	0.897 (5.518E^–48^)	0.872 (2.478E^–53^)
Heart	0.972 (5.002E^–88^)	0.983 (3.490E^–99^)
Kidneys	0.965 (3.095E^–71^)	0.946 (3.739E^–131^)
Liver	0.980 (9.285E^–97^)	0.980 (2.623E^–93^)
Lungs	0.874 (1.060E^–20^)	0.955 (3.011E^–68^)
Small intestine + Proventriculus and gizzard/Stomach	0.926 (2.024E^–36^)	0.800 (8.813E^–16^)
Spleen	0.750 (2.382E^–7^)	0.890 (2.029E^–22^)
**Organ length** cm		
Small intestine	0.838 (3.020E^–25^)	0.841 (3.049E^–9^)
**Physiological function**		
GFR ml min^–1^	0.874 (1.25E^–16^)	0.932 (1.49E^–10^)
**Mean ± (number of observations/species) SEM**		
Slope		
Organ weights g		
Brain	0.591 ± (95) 0.021	0.753 ± (117) 0.027 ***
Heart	0.926 ± (113) 0.0149	0.960 ± (112) 0.0120
Kidneys	0.865 ± (97) 0.0168	0.926 ± (206) 0.0155*
Liver	0.911 ± (114) 0.0124	0.884 ± (110) 0.0122
Lungs	0.900 ± (44) 0.0521	1.007 ± (100) 0.0218*
Small intestine + Proventriculus and gizzard/Stomach	1.038 ± (63) 0.0372	0.860 ± (45) 0.0122***
Spleen	0.956 ± (21) 0.122	0.941 ± (45) 0.0499
**Organ length** cm		
Small intestine	0.519 ± (61) 0.029	0.430 ± (21) 0.042
**Physiological function**		
GFR ml min^–1^	0.744 ± (35) 0.048	0.916 ± (18) 0.064*
**Intercept**		
Organ weights g		
Brain	–0.941 ± (95) 0.047	–1.210 ± (117) 0.109*
Hear	–1.800 ± (113) 0.0352	–2.061 ± (112) 0.0516***
Kidneys	–1.693 ± (97) 0.0387	–1.987 ± (206) 0.0535***
Liver	–1.372 ± (114) 0.0299	–1.132 ± (110) 0.0526***
Lungs	–1.607 ± (44) 0.1474	–1.977 ± (100) 0.0925*
Small intestine + Proventriculus and gizzard/Stomach	–1.322 ± (63) 0.0813	–0.610 ± (45) 0.298**
Spleen	–2.967 ± (21) 0.327	–2.337 ± (45) 0.225
**Organ length** cm		
Small intestine	0.504 ± (61) 0.076	0.971 ± (21) 0.168**
**Physiological function**		
GFR ml min^–1^	0.324 ± (35) 0.059	0.429 ± (18) 0.098

* Difference between birds and mammals P < 0.05, **P < 0.01, ***P < 0.001.

**TABLE 5:** Relationships between relative weights of organs (as % of body weight), relative lengths of the small intestine (cm g^–1^) and organ functions (per kg body weight) versus log_10_body weight (g) [for data from individual species see Supplementary Table 7 (mammals), 8 (birds)].

	Birds	Mammals
**Adjusted *R*^2^ [*P* = ]**		
**Organ weights**		
Brain	0.757 (1.625E^–30^)	0.297 (1.286E^–10^)
Heart	0.158 (7.558E^–6^)	0.0479 (0.01198)
Kidneys	0.386 (6.383E^–16^)	0.183 (8.786E^–11^)
Liver	0.250 (1.155E^–8^)	0.483 (2.188E^–17^)
**Organ lengths**		
Small intestine	0.708 (1.224E^–17^)	0.532 (0.000105)
**Physiological function**		
GFR	0.259 (0.0011)	0.206 (0.0336)
**Mean ± (number of observations/species) SEM**		
Slope		
Organ weights		
Brain	1.446 ± (95) 0.084	–0.543 ± (117) 0.077***
Heart	–0.189 ± (113) 0.040	–0.0599 ± (110) 0.023**
Kidneys	–0.304 ± (97) 0.087	–0.183 ± (206) 0.020
Liver	–0.517 ± (112) 0.083	–0.746 ± (110) 0.073*
**Organ lengths**		
Small intestine	–0.333 ± (61) 0.027	–0.236 ± (21) 0.048
**Physiological function**		
GFR	–1.966 ± (35) 0.548	–0.843 ± (18) 0.363
**Intercept**		
Organ weights		
Brain	5.300 ± (95) 0.194	3.248 ± (117) 0.312***
Heart	1.577 ± (113) 0.095	0.902 ± (110) 0.100***
Kidneys	1.789 ± (97) 0.089	1.197 ± (206) 0.069***
Liver	3.995 ± (112) 0.203	5.887 ± (110) 0.316***
**Organ lengths**		
Small intestine	1.195 ± 0.071	1.164 ± 0.196
**Physiological function**		
GFR	2.994 ± (35) 0.670	3.694 ± (18) 0.0.558

#### Small Intestine Weights and Lengths

The relative weight of the small intestine plus proventriculus/gizzard (birds) or stomach (mammals) was 47.4% greater (*P* = 0.0113) in mammals versus birds (Table 3). There were no differences in the relative small intestine length (cm kg^–1^) or villus height/length (duodenum, jejunum, and ileum) in birds and mammals (Table 3) (for data on individual species see Supplementary Tables 12, 13 for small intestine lengths together with Supplementary Table 14 for villus heights in mammals and birds).

#### Liver Composition and Cellularity

Table 3 summarizes data on the number of cells per unit mass of the liver together with liver composition and relative organ weights in birds and mammals. The cellularity was calculated from the DNA concentration and the mass of the genome (Table 3). Subsequently, the volume of liver cells was calculated. There were 4.46 fold more (*P* < 0.01) liver cells per g in birds than in mammals.

Total lipid concentrations in the liver were higher (38.8%) (*P* < 0.05) in birds than mammals (Table 1). There were, however, no differences in the relative concentrations of triglyceride, phospholipid and total cholesterol between mammals and birds (see Supplementary Data).

### Allometric Relationships

There were allometric relationships (adjusted *R*^2^ > 0.75) based on regressions between log_10_ organ weights and log_10_ body weights for the brain, heart, kidneys liver, lungs, stomach (mammals)/proventriculus + gizzard plus small intestine and spleen in both mammals and birds (Table 4). There were small but significant *(P* < 0.05) differences between mammals and birds for the slope [brain, kidneys, lungs and stomach (mammals)/proventriculus and gizzard plus small intestine] and intercept [brain, heart, kidneys, liver, lungs and stomach (mammals)/proventriculus and gizzard plus small intestine].

There were relationships between the relative weight of the liver and log_10_ body weight in both birds and mammals (Table 4). In both the birds and mammals, there were negative slopes in the relationship between relative organ weight and log_10_ body weight (see Table 4). Both the slope and intercept for the relationship for the liver were greater (respectively *P* = 0.0209 and *P* < 0.001) in mammals than birds (see Table 4). Moreover, there were a negative relationship (*P* < 0.001) between the relative weight of the heart and kidneys and log_10_ body weight in both birds and mammals (see Table 4). Both the slope and intercept for this relationship for the heart and kidney were greater or tended to be greater in birds than mammals [heart slope: *P* = 0.0059, intercept: *P* < 0.001; kidney slope: *P* = 0.0692, intercept: *P* < 0.001] (see Table 4). There was no relationship (*P* > 0.05) between the relative weight of the lungs and spleen and log_10_ body weight in either birds or mammals.

There were no relationships (*P* > 0.05) between relative weights of the following components of the gastro-intestinal tract and log_10_ body weight: small intestine [birds: adjusted *R*^2^ –0.00345 (*P* = 0.366)], proventriculus and gizzard [birds: adjusted *R*^2^ –0.0198 (*P* = 0.861)] and combined proventriculus, gizzard and small intestine [birds: adjusted *R*^2^ 0.0350 (*P* = 0.0765); mammals: adjusted *R*^2^ –0.00317 (*P* = 0.356)]. In contrast, there was a strong (*P* < 0.001) allometric relationship between log_10_ small intestine length and log_10_ body weight [adjusted *R*^2^ 0.838 (birds), 0.841 (mammals)]. The slopes of the relationships were similar in birds and mammals (Table 4) (for individual species data see Supplementary Tables 12, 13). The height/length of villi tended to be higher in birds than mammals (Table 4). However, there are limited reports of villus characteristics in birds (Table 4). Restricting analysis to a comparison between mammals and domesticated birds, the lengths/heights of duodenal villi are greater (*P* = 0.0006) in mammals than domesticated birds [duodenum: 1137 ± (3 species) 115.2 μm]. There are insufficient reports of absorptive area in avian species (only two species) for an allometric analysis.

### Glomerular Filtration Rates

Glomerular filtration rates per kg body weight (GFR) tended to be higher (*P* = 0.0722) in birds than mammals (Table 3) (for individual species data see Supplementary Table 15). There was a high coefficient of variation for GFR of 80.2% in birds. There were allometric relationships between log_10_ GFR and log_10_ body weight (*P* < 0.001) in both mammalian and avian species (Table 4). The slope was somewhat greater (23.1%) in mammals than birds (*P* < 0.05) (Table 4) but there was no difference in the intercept (*P* > 0.05). There was a relationship, albeit relatively small (adjusted *R*^2^ 0.2 – 0.3) (*P* < 0.05), between GFR (per kg body weight) and log_10_ body weight in both avian and mammalian species (see Table 5). However, the relationship did not differ (*P* > 0.05) between birds and mammals.

## Discussion

The presented differences in physiological, morphological and allometric parameters between birds and mammals (Tables 1–4) are important for the transfer of physiologically based kinetic (PBK) models. In cases of close similarity between birds and mammals, parameterization for PBK modeling can be facilitated.

Blood concentrations of hemoglobin and MCHC were identical between birds and mammals (Table 1). This is consistent with a physiological “blueprint” for birds and mammals existing prior to the divergence of the ancestors of birds and mammals about 315 million years ago (Benton and Donoghue, 2006; Ford and Benson, 2020). It is argued that the difference in hematocrit/PCV between avian and mammalian species (Table 1) was trivial and consistent with the same overall physiological “blueprint.” Supporting the physiological “blueprint” is the identical lung and liver relative weights (Table 3). However, the size of the hepatic cells and erythrocytes were, respectively, considerably smaller and larger in birds than mammals (Tables 1, 3). Plasma concentrations of total lipids, triglyceride, phospholipid, cholesterol, total protein and albumen differed between avian and mammalian species (Table 1).

Plasma/serum concentrations of total protein are similarly low in reptiles [Crocodilia: 5.83 g dL^–1^; Testudines: 4.46 g dL^–1^, Squamata: 5.01 g dL^–1^] (Dessauer, 1970). Moreover, in recent studies, plasma/serum albumin concentrations are low [e.g., Chinese alligators: 2.2 g dL^–1^ (Tang et al., 2018); American alligators: 1.1 g dL^–1^ (Hamilton et al., 2016)]. There is a corollary to changes in albumen etc. and that is when a chemical agent is transported into the yolk bound to albumen. A decrease in plasma concentrations of albumen etc. will be expected to be accompanied by increases in the fraction unbound.

There were marked differences in the relative weights of the kidneys, heart, spleen, oviduct, and ovary between avian and mammalian species (Table 1). It is speculated that the increased relative size of the heart in birds represents an adaption to the metabolic needs of flight as is the case in bats (e.g., Maina and King, 1984; Maina, 2000; Canals et al., 2009).

What are the implications of structural and size differences between erythrocytes in mammals and birds? Mammalian erythrocytes are biconcave discoids; there being increased surface area allowing erythrocytes to deform. The surface area of human erythrocytes is estimated as 40% greater than for a sphere of the same volume based on shape (Mohandas and Gallagher, 2008). In contrast, avian erythrocytes are spheroid with the shape less effective in increasing surface area than the biconcave discoids in mammals. A further consideration is the smaller MCV in mammals than birds (Table 1). The corollary is that this further increases the surface area of mammalian erythrocytes compared to avian erythrocytes.

The corollary to the reduction in MCV in gallinaceous birds compared to other avian species is that it increases the surface area (per unit of blood) across which oxygen and chemical agents can pass. It is suggested that increased surface area of erythrocytes in gallinaceous birds allows PCV to decrease. The ancestors of the Galliformes diverged from those of Anseriforms about 100 million years ago in the middle of the Cretaceous period and from the Neoaves somewhat earlier in the Cretaceous period (e.g., Pereira and Baker, 2006).

We would argue that reproductive strategy is responsible for the differences in multiple parameters where sexually mature females diverge from sexually immature females/males. The Hct/PCV was depressed in sexually mature female birds compared to sexually immature female birds and male birds (Table 2). This is consistent with the conclusions of Wagner et al. (2008) based on their studies in zebra finches. In contrast, a lack of differences in Hct/PCV was reported between male and female (presumably sexually immature) across multiple avian species (Fair et al., 2007). It is suggested that the reduction in Hct/PCV in sexually mature female birds compared to prepubertal females/males reflects adaptations to egg laying with the plasma providing the following for egg production:

• Glucose and fatty acids to fuel production of the egg.
• Amino-acids for the synthesis of egg white proteins together with shell membranes in the oviduct and of yolk precursors in the liver.
• Triglycerides and other lipids as yolk precursors.
• Calcium and bicarbonate for the production of the calcareous shell.

Moreover, compatible with the needs of egg production, there were marked higher plasma concentrations of lipid and triglyceride together with ovarian and oviductal weights in sexually mature females versus prepubertal female and male birds (Table 2).

Two of the major yolk precursors synthesized in the liver are vitellogenin and very-low-density lipoprotein (VLDL). Transport of these yolk precursors into the oocyte is mediated by the same receptor, the VLDL receptor (Stifani et al., 1990; Barber et al., 1991; Bujo et al., 1994; reviewed: Bujo et al., 1997). As female birds transition from non-laying to laying there are large increases in plasma concentrations of both vitellogenin [increasing from non-detectable to 1.63 g dL^–1^] (chicken: Redshaw and Follett, 1976) and VLDL [European starling (*Sturnus vulgaris*): non-laying – 0.3 g dL^–^1, egg laying – 2.7 g dL^–1^] (Challenger et al., 2001). Similarly, the plasma concentrations of apolipoprotein VLDL-11 (apo-II) increases from less than 0.0005 mg dL^–1^ in sexually immature females to 159.9 mg dL^–1^ in laying hens (chicken: Pinchasov et al., 1994). Other constituents of the yolk include albumin (referred to as α-livetin) and immunoglobulin Y (γ-livetin). Surprisingly, there is no difference between plasma concentrations of albumin in the plasma in sexually mature female versus sexually immature female birds/male birds (Table 2). This would have been predicted based on the flux of albumin into the oocyte.

There were large increases in the plasma concentrations of total lipid and triglyceride in sexually mature female birds compared to pre-pubertal females and male birds (Table 2). These, reflect their role as yolk precursors. Triglycerides also may bind lipid soluble vitamins and other hydrophobic chemical agents.

Surprisingly, avian liver cells were markedly smaller than their mammalian counterparts (Table 3). The veracity of this is supported by the compliance between the calculated liver cell volume and the hepatic cell volume determined by morphometric analysis. The volume of hepatocytes in late embryonic chick development was estimated based on microscopic analysis as 1.014 pL (Wong and Cavey, 1992). This is within 25% of that estimated from DNA (Table 3).

It is noted that the calculated number of cells (as opposed to hepatocytes) per gram of liver in the present study was determined by dividing the DNA concentration by the mass of the genome. The calculated concentrations in the present study (Table 3) were 2–5 folds higher than those previously reported (476 million per gram in rat – see Supplementary Table 9). For instance, the numbers of cells per gram of rat liver were approximately 120 million per gram (Bayliss et al., 1990); there being the following multiple consistent numbers of cells per gram of liver: 85 million cells (Carlile et al., 1997), 117 million cells (Sohlenius-Sternbeck, 2006), 128 million cells (Seglen, 1973; Zahlten and Stratman, 1974). The reason for this discrepancy is not readily apparent. In the present study, total liver cells were estimated as opposed to hepatocytes. Another possible explanation are the presence of binucleate cells (20%) in the liver (Seglen, 1973). Irrespective of the absolute numbers, there was a marked difference in the number of cells per gram was higher in birds than mammals largely due to the smaller genome size. For PBK modeling, it is open to question whether the number of cells per gram and cellular volume (Table 3) may be used.

The presence of allometric relationships (Table 4) is not surprising given, for instance, the previous report of allometric relationships of heart weight in birds (Grubb, 1983). Moreover, allometric relationships impact physiological parameters (e.g., in mammals – Lindstedt and Schaeffer, 2002; White and Seymour, 2005) and pharmacokinetics (e.g., in birds: Knibbe et al., 2005).

The difference in ovary weight between avian and mammalian species is probably inconsequential to PBK modeling. However, the large increases in the weights of the ovary and oviduct during sexual maturity may impact PBK modeling with chemical agents passing into the yolk along with VLDL and/or vitellogenin (VTG) via a receptor mediated process. In an analogous manner, riboflavin binds to riboflavin binding protein (ribBP) with ribBP associating with vitellogenin (VTG) in the circulation and thereby transported into the oocyte (reviewed: Bujo et al., 1997).

Despite the higher relative kidney weights in birds than mammals (Table 3), overall kidney functioning is similar in birds and mammals (e.g., GFR) (Table 3). This would suggest that the avian kidney was less efficient than the mammalian homolog. This may be explicable with the types of nephrons present. The avian kidney has mammalian type (both long and short) together with reptilian type nephrons (i.e., lacking loops of Henle) (Braun, 1976). The glomerular volume per nephron has been estimated as follows in the desert quail (a Galliform species): mammalian long loop –15.8 nL min^–1^, mammalian short loop – 10.9 nL min^–1^ and reptilian –6.4 nL min^–1^ (Braun and Dantzler, 1972).

Allometric relationships between log_10_ small intestine length and log_10_ body weight have been reported previously for both mammalian and avian species (Caviedes-Vidal et al., 2007). There were no differences in the slopes for birds greater than 195 g in body weight and mammals. In contrast, birds with body weights of less than 195 g, have shorter relative small intestine lengths than larger birds or mammals (Caviedes-Vidal et al., 2007).

There were high coefficients of variation m in GFR. Such high coefficients of variation have been reported previously (Braun, 1982). In the present study, there were allometric relationships between log_10_ GFR and log_10_ body weight based on 18 mammalian species and 35 avian species (Table 4). Similarly, allometric relationships for GFR have been reported for birds based on 11 species (Yokota et al., 1985) and mammals based on 12 species (Singer and Morton, 2000; Singer, 2001). The slope of the allometric relationship in the present study for birds (Table 4) was identical to that of Yokota et al. (1985) but higher for mammals than that of Singer and Morton (2000) (slope 0.77). There are shifts in GFR with physiological state (e.g., fasting in the broad-tailed hummingbird: Hartman Bakken et al., 2004 and water loading in desert quail: Braun and Dantzler, 1975). It is reasonable to suggest that chemical agents that influence GFR are indirectly influencing their clearance.

The greater relative weights of the kidney in birds may impact PBK modeling as there may be renal re-cycling of a chemical agent or its metabolites. Recycling involves a chemical agent or its metabolite passing from the blood to the kidneys where it is filtered into nephrons and hence into the urine. This passes from the cloaca to the colon and caeca by retrograde peristalsis (Hughes et al., 1999) and is then absorbed (Son and Karasawa, 2000) into the blood stream.

In view of the binding of fatty acids to albumin (reviewed Spector, 1984), the reduced concentration of albumin in birds may impact transport of fatty acids and of drugs, pesticides and other chemicals that bind to albumin. Moreover, were a drug, pesticide and chemical agent to bind to another protein, hemoglobin, then the lower blood concentrations of hemoglobin in gallinaceous versus non-gallinaceous birds and between sexually mature female birds and prepuberal females together with males might be expected to influence PBK and other modeling.

## Conclusion

A database of physiological and anatomical data across avian species has been developed. This has allowed a series of comparisons with mammalian species and, consequently, an understanding of the major differences. Moreover, the availability of physiological and anatomical data for birds provides a critically important step toward the development of PBK models for multiple avian species. What is needed now is the development of a PBK model for multiple compounds in birds. What is missing is an analysis of the composition of organs due to the lack of available data. Mammalian parameters will need to be used in any PBK model. In addition, any such model will require refinement with the addition of more information quantifying the physiology of birds.

## Data Availability Statement

The original contributions presented in the study are included in the article/Supplementary Material, further inquiries can be directed to the corresponding author/s.

## Author Contributions

CS, JW, ME, SS, VB, AB, TP, and DH conceived the study, revised the manuscript, and critiqued analysis. CS and VB collected the data. CS conducted the analyses and drafted the manuscript. All the authors contributed to the article and approved the submitted version.

## Conflict of Interest

JW, ME, AB, TP, and DH were employed by the company Bayer AG. SS and VB were employed by the company esqLABS GmbH. CS declares that his company Scanes Technology and Research and esqLABS GmbH received funding from Bayer AG. The funder (in the person of the Bayer authors) had the following involvement with the study: contributed to conceiving the study, revising the manuscript, and critiquing the analysis.

## Publisher’s Note

All claims expressed in this article are solely those of the authors and do not necessarily represent those of their affiliated organizations, or those of the publisher, the editors and the reviewers. Any product that may be evaluated in this article, or claim that may be made by its manufacturer, is not guaranteed or endorsed by the publisher.
