# Supplementary material for: Quantitative Comparison of Avian and Mammalian Physiologies for Parameterization of Physiologically Based Kinetic Models
Source: Front Physiol. 2022 Apr 5;13:858386. doi: 10.3389/fphys.2022.858386 (PMC9016154; doi:10.3389/fphys.2022.858386)
Supplement: Supplementary file 1 [file Table_1.DOCX]

**SUPPLEMENTARY DATA**

**Supplementary Table 1.** Hematocrit and other blood parameters in individual mammalian species. These data were employed in Table 1

| Animal | species | Pcv % | Hb  % | Rbc  # x 10^6^ µL^-1^ | Mcv fL | references |
| --- | --- | --- | --- | --- | --- | --- |
| Sub-class *Prototheria* |  |  |  |  |  |  |
| Order *Monotremata* |  |  |  |  |  |  |
| Platypus | *Ornithorhynchus anatinus* | 49.0 | 18.0 | 8.97 | 52.0 | Mean of Clark, 2004 and Geraghty *et al*., 2011 |
| Short-beaked Echida | *Tachyglossus aculeatus* | 42.9 | 15.9 | 6.7 | 66.6 | mean 9 studies cited in Clark, 2004 |
| Long beaked Echida | *Zaglossus bruijnii* | 29 | 12.6 | 1.5 | 198 | Cited Clark, 2004 |
|  |  |  |  |  |  |  |
| Sub-class *Marsuliala* |  |  |  |  |  |  |
| Order *Dasyuromorphia* |  |  |  |  |  |  |
| Family *Dasyuridae* |  |  |  |  |  |  |
| Brown Antechinus | *Antechinus stuartii* | 44.6 | 14.1 | 9. 34 | 42.2 | Mean 3 studies cited in Clark, 2004 |
| Eastern Quoll | *Dasyurus viverrinus* | 45.0 | 16.9 | 10.3 | 43.3 | Mean from 4 studies cited in Clark, 2004 |
| Northern Quoll | *Dasyurus hallucatus* | 46.2 | 15.6 | - |  | Mean from 3 studies cited in Clark, 2004 |
| Western Quoll | *Dasyurus geoffroii* | 40.9 | 14.5 | 7.91 | 53 | Mean from 4 studies cited in Clark, 2004 |
| Fat-tailed Dunnart | *Sminthopsis crassicaudata* | 34.5 | 11.4 | 6.75 | 39.4 | Mean from 3 studies cited in Clark, 2004 |
| Striped-faced Dunnart | *Sminthopsis macroura* | - | - | 6.65 | - | Mean from 3 studies cited in Clark, 2004 |
| Kowari | *Dasyuroides byrnei* | 55 | 16.7 | 8.1 | 64 | Clark, 2004 |
| Red-tailed phascogale | *Phascogale calura* | 47.5 | 16.4 | 11.15 | 45 | Mean from 2 studies cited in Clark, 2004 |
| Brush-tailed phascogale | *Phascogale tapoatafa* | 46 | 16.15 | 9.2 | 51 | Clark, 2004 |
| Tasmanian devil | *Sarcophilus harrisii* | 41.3 | 15.46 | 6.42 | 67 | Mean 5 studies cited in Clark, 2004 |
| Order *Didelphimorphia* |  |  |  |  |  |  |
| Family *Didelphidae* |  |  |  |  |  |  |
| American Woolly Opossum | *Caluromys derbianus* | 37 | 13.8 | 4.7 | 78.8 | [Rothstein and](http://www.ncbi.nlm.nih.gov/pubmed?term=Rothstein%20R%5BAuthor%5D&cauthor=true&cauthor_uid=4336994) [Hunsaker,](http://www.ncbi.nlm.nih.gov/pubmed?term=Hunsaker%20D%202nd%5BAuthor%5D&cauthor=true&cauthor_uid=4336994) 1972 |
| Grayshort-tailed Opossum | *Monodelphis domestica* | 34.9 | 13.1 | 6.61 | 53 | Evans *et al*., 2010 |
| Virginia Opossum | *Didelphis virginiana* | 39 | 11.2 | 4.9 | 82 | Youatt *et al*., 1961 |
| Order *Diprotodontia* |  |  |  |  |  |  |
| Family *Macropodidae* |  |  |  |  |  |  |
| Agile Wallaby | *Macropus agilis* | 49 | 16.7 | 5.38 | 92.5 | Clark, 2004 |
| Antilopine Kangaroo | *Macropus antilopinus* | 45 | 15.3 | 7.37 | 61 | Cited in Clark, 2004 |
| Black Striped Wallaby | *Macropus dorsalis* | 52 | 17.3 | 6.84 | 76 | Clark, 2004 |
| Common Wallaroo | *Macropus robustus* | 42 | 12.4 | 7.37 | 78 | mean 5 studies cited in Clark, 2004 |
| Eastern Grey Kangaroo | *Macropus giganteus* | 45.1 | 15.3 | 5.86 | 81 | mean 5 studies cited in Clark, 2004 |
| Parma Wallaby | *Macropus parma* | 47.2 | 14.8 | 7.11 | 68.7 | Clark *et al*., 2003; Clark, 2004 |
| Red Kangaroo | *Macropus rufus* | 44.3 | 16.4 | 4.95 | 94.7 | Mean from 9 studies cited in Clark, 2004 |
| Red-necked Wallaby | *Macropus rufogriseus* | 47.3 | 16.47 | 5.42 | 87.5 | Mean 6 studies cited in Clark, 2004 |
| Tammar Wallaby | *Macropus eugenii* | 37.5 | 14.2 | 5.90 | 73.2 | Mean 6 studies cited in Clark, 2004 |
| Western Grey Kangaroo | *Macropus fuliginosus* | 47.5 | 16.6 | 5.25 |  | Cited in Clark, 2004 |
| Whip-tail Wallaby | *Macropus parryi* | 52 | 17.4 | 4.93 | 113 | Clark, 2004 |
| Bridled Nail-tail Wallaby | *Onychogalea fraenata* | 51 | 17.1 | 5.65 | 91 | Clark, 2004 |
| Northern Nail-tail Wallaby | *Onychogalea unguifera* | 62 | 21.7 | 5.80 | 107 | Clark, 2004 |
| Swamp Wallaby | *Wallabia bicolor* | 52 | 17.3 | 6.86 | 71 | Clark, 2004 |
| Allied Rock-wallaby | *Petrogale assimilis* | 41.2 | 13.5 | 5.01 | 82.5 | Mean 3 studies cited in Clark, 2004 |
| Brush-tailed Rock-Wallaby | *Petrogale penicillata* | 42 | 14.9 | 5.9 | 70 | Barnes *et al*., 2008 |
| Proserpine Rock-wallaby | *Petrogale persephone* | 46 | 14.9 | 6.81 | 65 | Clark, 2004 |
| Purple-necked Rock-wallaby | *Petrogale purpureicollis* | 55 | 18.9 | 5.87 | 94 | Clark, 2004 |
| Unadorned Rock-wallaby | *Petrogale inornata* | 37 | 11.6 | 5.09 | 72 | Clark, 2004 |
| Yellow-footed Rock-wallaby | *Petrogale xanthopus* | 44 | 14.6 | 5.54 | 79 | Clark, 2004 |
| Rufous Hare-Wallaby | *Lagorchestes hirsutus* | 55 | 18.9 | 8.0 | 69 | Clark, 2004 |
| Spectacled Hare-Wallaby | *Lagorchestes conspicillatus* | 51.5 | 17.5 | 6.65 | 69 | Clark, 2004 |
| Quokka | S*etonix brachyurus* | 41.0 | 14.3 | 6.19 | 62.2 | Mean 9 studies cited in Clark, 2004 |
| Red-legged Pademelon | *Thylogale stigmatica* | 52.0 | 17.03 | 7.36 | 70 | Mean 3 studies cited in Clark, 2004 |
| Tasmanian Pademelon | *Thylogale billardierii* | 46.5 | 16.22 | 6.32 | 77.2 | Mean 2 studies cited in Clark, 2004 |
| Goodfellow’s Tree-kangaroo | *Dendrolagus goodfellowi* | 50 | 17.5 | 6.10 | 81.4 | Clark, 2004 |
| Lumholtz’s tree-kangaroo | *Dendrolagus lumholtzi* | 46 | 15.5 | 5.72 | 81 | Clark, 2004 |
| Matschie’s Tree- kangaroo | *Dendrolagus matschiei* | 46.2 | 17.0 | 6.18 | 77 | Mean 2 studies cited in Clark, 2004 |
| Family *Phascolarctidae* |  |  |  |  |  |  |
| Koala | *Phascolarctos cinereus* | 38.96 | 12.19 | 3.54 | 112.2 | Mean 13 studies cited in Clark, 2004 |
| Family *Phalangeridae* |  |  |  |  |  |  |
| Common Brush-tailed Possum | *Trichosurus vulpecula* | 40.6 | 13.3 | 6.09 | 67.0 | mean 9 studies cited in Clark, 2004 |
| Mountain Brushtail Possum | *Trichosurus cunninghami* | 36.0 | 12.3 | 4.95 | 74.1 | Clark, 2004 |
| Scaly-tailed Possum | *Wyulda squamicaudat* | 43.0 | 14.0 | - | - | mean 2 studies cited in Clark, 2004 |
| F[amily](http://en.wikipedia.org/wiki/Family_(biology)) *Potoroidae* |  |  |  |  |  |  |
| Boodie | *Bettongia lesueur* | 40 | 10.7 |  |  | Clark, 2004 |
| Gilbert’s Potoroo | *Potorous gilbertii* | 36.0 | 12.58 | 6.20 | 61.8 | Vaughan *et al*., 2009 |
| Long-nosed Potoroo | *Potorous tridactylus* | 49.0 | 15.7 | 8.95 | - | Mean 3 studies cited in Clark, 2004 |
| Rufous Rat-kangaroo | *Aepyprymnus rufescens* | 50.0 | 16.5 | 6.08 | 83 | Clark, 2004 |
| Family Mean |  | 42.7  + (4)  3.42 | 13.9  + (4)  1.35 | 7.08 + (4) 0.94 |  |  |
| Family *Pseudocheiridae* |  |  |  |  |  |  |
| Common Ringtail Possum | *Pseudocheirus peregrinus* | 44.0 | 14.25 | 5.57 | 79.25 | mean 2 studies cited in Clark, 2004 |
| Herbert River Ringtail Possum | *Pseudochirulus herbertensis* |  |  |  |  | Clark, 2004 |
| Greater Glider | *Petauroides volans* | 37.0 | 12.15 | 5.40 | 66.0 | Clark, 2004 |
| Sugar Glider | *Petaurus breviceps* | 45.5 | 14.5 | 7.40 | 64 | Clark, 2004 |
| Scaly-tailed possum | *Wyulda squamicaudata* | 43.0 | 14.05 | - | - | mean 2 studies cited in Clark, 2004 |
| F[amily](http://en.wikipedia.org/wiki/Family_(biology)) *Vombatidae* |  |  |  |  |  |  |
| Common Wombat | *Vombatus ursinus* | 38.08 | 12.6 | 5.438 | 69.1 | Mean 6 studies cited in Clark, 2004 |
| Northern Hairy-nosed Wombat | *Lasiorhinus krefftii* | 36.5 | 12.3 | 4.27 | 85.5 | Reiss *et al*., 2008 |
| Southern Hairy-nosed Wombat | *Lasiorhinus latifrons* | 41 | 13.5 | 5.04 | 80.5 | Mean 5 studies cited in Clark, 2004 |
|  |  |  |  |  |  |  |
| Order *Peramelemorphia* |  |  |  |  |  |  |
| Family *Thalacomyidae* |  |  |  |  |  |  |
| Bilby | *Macrotis lagotis* | 55 | 17.1 | 7.47 | 73 | Clark, 2004 |
| Family *Peramelidae* |  |  |  |  |  |  |
| Eastern Barred Bandicoot | *Perameles gunnii* | 45 | 16.1 | - | - | Clark, 2004 |
| Northern Brown Bandicoot | *Isoodon macrourus* | 41 | - | - | - | Mean 2 studies cited in Clark, 2004 |
| Southern Brown Bandicoot | *Isoodon obesulus* | 44 | 14.6 | - | - | Clark, 2004 |
| Super-order *Afrotheria* |  |  |  |  |  |  |
| Order | Order |  |  |  |  |  |
| African Elephant | *Loxadonta africana* |  | 7.08 | 3.77 |  | Debbie and Clausen, 1975 |
| Asian Elephant | *Elephas maximus* | 38 | 12.7 | 3.2 | 118 | Silva and Kuruwita, 1993 |
| Order *Hyracoidea* |  |  |  |  |  |  |
| Rock hyraxes | *Procavia capensis* | 31.4 | 8.3 | 4.66 | 69.5 | Aroch *et al*., 2007 |
| Order |  |  |  |  |  |  |
| West Indian Manatee | *Trichechus manatus* | 35.1 | 11.2 | 2.76 | 128 | Harvey *et al*., 2009 |
| Super-order *Xenarthra* |  |  |  |  |  |  |
| Order *Cingulata* |  |  |  |  |  |  |
| Big Hairy Armadillo | *Chaetophractus villosus* | 34.7 | 19.95 | 3.83 | 91.6 | [Casanave](http://link.springer.com.ezproxy.lib.uwm.edu/search?facet-author=%22Dra.+E.+B.+Casanave%22) and [Polini](http://link.springer.com.ezproxy.lib.uwm.edu/search?facet-author=%22N.+N.+Polini%22), 1999 |
| Nine-banded armadillo | *Dasypus novemcinctus* | 38 |  | 3.0 |  | Deem *et al.,* 2009 |
| Three-Banded armadillo | *Tolypeutes matacus* | 33 |  | 3.1 |  | Deem *et al.,* 2009 |
| Pichi | *Zaedyus pichiy* | 49.3 | 16 | 4.30 | 120.2 | Superina *et al*., 2008 |
| Order *Pilosa* |  |  |  |  |  |  |
| Hoffmann's Two-Toed Sloths | *Choloepus hoffmanni* | 39.9 | 14.25 | 3.15 | 122.7 | Wallace and Oppenheim, 1996 |
| Southern Two -toed Sloth | *Choloepus didactylus* | 35.7 | 11.5 | 2.6 | 135.9 | Vogel *et al*., 1999 |
| Giant Anteater | *Myrmecophaga tridactyla* | 37.7 | 11.8 | 2.36 | 165.2 | Sanches *et al*., 2013 |
| Collared Anteater | *Tamandua tetradactyla* | 34.8 | 10.73 | 3.15 | 116.1 | Sanches *et al*., 2013 |
| Order *Cetacea* |  |  |  |  |  |  |
| Family *Balaenopteridae* |  |  |  |  |  |  |
| Blue Whale | *Balaenoptera musculus* |  | 9.6 | 3.84 |  | Clark, 2004 |
| Bryde’s Whale | *Balaenoptera edeni* | 48 | 15.4 | 3.6 | 133 | Priddel and Wheeler, 1998 |
| Bowhead whale | *Balaena mysticetus* | 59 | 20.3 | 3.3 | 178 | Castellini *et al*., 2010 |
| Common Minke Whale | *Balaenoptera acutorostrata* | 50 | 18.25 |  |  | Clark, 2004 |
| Fin Whale | *Balaenoptera physalus* |  |  |  |  | Clark, 2004 |
| Sei Whale | *Balaenoptera borealis* |  | 15.6 |  |  | Clark, 2004 |
| Family *Eschrichtiidae* |  |  |  |  |  |  |
| Gray Whale | *Eschrzchtzus robustus* | 48 | 14.0 | 3.3 | 129 | Cited in Priddel and Wheeler, 1998 |
| Family *Delphinidae* |  |  |  |  |  |  |
| Killer Whale | *Orcinus orca* | 44.4 | 15.9 | 4.03 | 110.8 | mean 7 studies summarized in Clark, 2004 |
| False Killer Whale | *Pseudorca crassidens* | 48.0 | 15.2 | 4.43 | 108 | Clark, 2004 |
| Short-finned Pilot Whale | *Globicephala macrorhynchus* | 43.3 | 15.7 | 3.69 | 116 | mean 3 studies summarized in Clark, 2004 |
| Common Bottlenose Dolphin | *Tursiops truncatus* | 44.7 | 15.4 | 3.90 | 115.2 | mean 9 studies Clark, 2004 |
| Hector's Dolphin | *Cephalorhynchus hectori* |  | 19.45 |  |  | Clark, 2004 |
| Pacific White-sided Dolphin | *Lagenorhynchus obliquidens* | 48.9 | 17.1 | 5.32 | 92.4 | Shirai and Sakai, 1998 |
| Risso's Dolphin | *Grampus griseus* | 51.5 | 21.4 | 4.63 | 112.5 | mean 2 studies Clark, 2004 |
| Short-beaked Common Dolphin | *Delphinus delphis* | 50.5 | 17.7 | 4.75 | 107 | Clark, 2004 |
| Striped Dolphin | *Stenella coeruleoalba* | 56.5 | 20.9 | - | - | Clark, 2004 |
| Family *Monodontidae* |  |  |  |  |  |  |
| Beluga whale | *Delphinapterus leucas* | 53 | 19.9 | 3.7 | 145.4 | Norman *et al*., 2012 |
| Family *Physeteridae* |  |  |  |  |  |  |
| Sperm whale | *Physeter macrocephalus* | 42 | 15.1 | 2.10 | 195 | Clark, 2004 |
| Pygmy Sperm Whale | *Kogia breviceps* | 50 | 15.7 |  |  | Clark, 2004 |
| Mean Order |  | 49.3  + (15) 1.25 | 16.8  + (18) 0.68 | 3.89 + (13) 0.22 | 128.5  + (12) 8.81 |  |
| Order *Carnivora* |  |  |  |  |  |  |
| Family *Phocidae* |  |  |  |  |  |  |
| Crab-eater Seal | *Lobodon carcinophaga* |  | 20.0 | 4.31 |  | mean 5 studies Clark, 2004 |
| Harbor Seal | *Phoca vitulina* | 57.8 | 19.8 | 5.81 | 102 | Greig *et al*., 2010 |
| Harp Seal | *Phoca groenlandica* | 55 | 23 | 3.85 | 135 | Boily *et al*., 2006 |
| Hooded Seal | *Cystophora cristata* | 61.5 | 26.3 | 3.88 | 141.5 | Boily *et al*., 2006 |
| Leopard Seal | *Hydrurga leptonyx* | 50 | 18.9 | 4.59 | 105.5 | mean 2 studies Clark, 2004 |
| Northern Elephant Seal | *Mirounga angustirostris* | 57 | 22.3 | 2.50 | 225 | Yochem *et al*., 2008 |
| Southern Elephant Seal | Mirounga leonina | 59.5 | 19.5 | 5.38 | 184.7 | Mean 6 studies cited in Clark, 2004 |
| Ringed Seal | *Pusa hispida* | 51 | 26 | 4.2 | 122 | Castellini *et al*., 2010 |
| Weddell Seal | *Leptonychotes weddellii* | 50.1 | 21.4 | 3.73 | 168 | Mean 6 studies cited in Clark, 2004 |
| Family *Otariidae* |  |  |  |  |  |  |
| Antarctic Fur Seal | *Arctocephalus gazella* | 49 | - | 6.2 | 78.5 | Fayolle *et al*., 2000 |
| Australian Sea Lion | *Neophoca*  *cinerea* | 56.3 | 19 | 5.53 | 102.9 | Needham *et al*., 1980 |
| California Seal Lion | *Zalophus*  *californianus* | 48.5 | 17.5 | 4.5 | 106.5 | Calculated from Norberg *et al*., 2011 |
| New Zealand Sea lion | *Phocarctos hookeri* | 51 | - | - | - |  |
| Northern Fur Seal | *Callorhinus ursinus* | 46 | 15.1 | 4.6 | 101 | Norberg *et al*., 2011 |
| Steller Sea lion | *Eumetopias jubatus* |  | 17.6 | 4.3 | 115.2 | Calculated from Richmond *et al*., 2005 |
| Family *Mustelidae* |  |  |  |  |  |  |
| Eurasian otter | *Lutra lutra* | 54.7 | 15.1 | 6.4 | 85.2 | Fernández-Morán et al., 2001 |
| North American River Otter | *Lontra Canadensis* | 47.6 | 15.1 | 10.99 | 43.3 | Tocidlowski *et al*., 2000 |
| Sea Otter | *Enhydra lutris* | 55.4 | 17.92 | 5.11 | 110.2 | Williams and Pulley, 1983 |
| Giant Otter | *Pteronura brasiliensis* | 50.7 | 16.4 | 6.42 | 74.6 | Rosas *et al*., 2008 |
| European Badger | *Meles meles* | 33.2 | 11.2 | 7.1 | 46.2 | [Mahmood](http://www.ncbi.nlm.nih.gov/pubmed/?term=Mahmood%20KH%5Bauth%5D) *et al*., 1988 |
| Ferret | *Mustela putorius* | 45.9 | 15.1 |  |  | Lee *et al*., 1982 |
| Long-tailed Weasel | *Mustela frenata* | 44 | 12.3 | 7.4 | 36 | Youatt *et al*., 1961 |
| American Mink | *Mustela vison* | 46.6 | 15.5 | 7.90 | 59.0 | Weiss *et al*., 1994 |
| Family *Feverridae* |  |  |  |  |  |  |
| Common Palm Civet | *Paradoxurus hermaphroditus* | 41.7 | 13.3 | 13.3 | 32.5 | Salakij *et al.,* 2007 |
| [Family](http://en.wikipedia.org/wiki/Family_(biology)) *Ursidae* |  |  |  |  |  |  |
| American Black Bear | *Ursus americanus* | 45.5 | 15.3 | 6.75 | 67.5 | Schoeder, 1987 |
| Andean Bear | *Tremarctos ornatu* | 43 | 14.4 | 7.87 | 53.2 | Castellanos *et al*., 2010 |
| Asiatic Black Bear | *Ursus thibetanus* | 43.2 | 15.3 | 6.44 | 66.4 | Pospísil *et al*., 1987a;  Chang *et al*., 2006 |
| Brown Bear | *Ursus arctos* | 46.7 | 15.7 | 6.48 | 71.9 | Kusak *et al*., 2005 |
| Malayan Sun Bear | *Helarctos malayanus* | 36.7 | 13.0 | 5.06 | 73.6 | Bush *et al*., 1980 |
| Sloth Bear | *Melursus ursinus* | 42.25 | 15.2 | 5.92 | 73.8 | Mean Bush *et al*., 1980; Shanmugam *et al*., 2008 |
| Spectacled Bear | *Tremarctos ornatus* | 44.3 | 15.4 | 8.43 | 50.4 | Bush *et al*., 1980 |
| Family *Ailuridae* |  |  |  |  |  |  |
| Red Panda  *Ailurus fulgens* | *Ailurus fulgens* | 42.4 |  | 9.0 |  | Wolff *et al*., 1990 |
| Family *Mephitidae* |  |  |  |  |  |  |
| Striped Skunk | *Mephitis mephitis* | 38.8 | 12.2 | 7.33 | 53.0 | Mustonen et al., 2013 |
| Western Spotted Skunk | *Spilogale gracilis* | 33.6 | 10.4 | 7.54 | 44.2 | Crooks *et al*., 2003 |
| Family *Procyonidae* |  |  |  |  |  |  |
| Raccoon | *Procyon lotor* | 31 | 8.3 | 8.7 | 37 | Youatt *et al*., 1961 |
| Family *Felidae* |  |  |  |  |  |  |
| Canada Lynx | *Lynx canadensis* | 39 | 14.0 | 7.40 | 52 | Moen et al., 2010 |
| Eurasian Lynx | *Lynx lynx* | 39.2 | 14.8 | 8.51 | 46.4 | Pospísil *et al*., 1987b |
| European Wildcat | *Felis silvestris* | 37.7 | 12.1 | 9.38 | 40.4 | Marco *et al*., 2000 |
| Sand Cat | *Felis margarita* | 46.2 | 13.6 | 9.4 |  | Chege *et al*., 2013 |
| Bobcat | *Felis rufus* | 36.44 | 11.88 | 8.03 | 44.42 | Miller *et al.,* 1999 |
| Fishing Cat | Felis viverrina | 41.5 | 12.1 | 6.9 | 63.1 | Prihirunkit *et al*., 2007 |
| Cougar | *Felis concolor* | 43 | 16.39 | 8.51 | 46.3 | Pospísil *et al*., 1987b |
| Jungle Cat | *Felis chaus* | 36.0 | 12.0 | 6.14 | 58.3 | Salakij *et al*., 2010 |
| Pampas Cat | *Leopardus colocolo* | 47.8 |  | 6.8 |  | Beltrán *et al*., 2009 |
| Clouded Leopard | *Neofelis nebulosa* | 41.4 | 13.53 | 7.09 | 58.6 | Mean: Pospísil *et al*., 1987b; Singh *et al*., 1999; Salakij *et al*., 2008a |
| Jaguar | *Panthera onca* | 36.65 | 12.255 | 7.96 | 46.3 | Mean: Pospísil *et al*., 1987b; Mussart *et al*., 2009 |
| Tiger | *Panthera tigris* | 53.4 | 15.7 | 8.7 | 54.45 | Mean: Pospísil *et al*., 1987b; Singh *et al*., 1999; Sajjad *et al.,* 2012 |
| Leopard | *Panthera pardus* | 44.9 | 14.6 | 8.58 | 53.55 | Mean: Pospísil *et al*., 1987b; Singh *et al*., 1999 |
| Lion | *Panthera leo* | 40.85 | 13.85 | 8.47 | 48.8 | Mean: Pospísil *et al*., 1987b; Maas et *al*., 2013 |
| Cheetah | *Acinonyx jubatus* | 38.45 | 13.375 | 7.445 | 52 | Mean: Pospísil *et al*., 1987b; Bechert *et* *al*., 2002 |
| Family *Herpestidae* |  |  |  |  |  |  |
| Egyptian Mongoose | *Herpestes*  *Ichneumon* | 41.7 | 14.9 | 8.6 | 48.3 | Palomares *et al*., 1992 |
|  |  |  |  |  |  |  |
| Family *Hyaenidae* |  |  |  |  |  |  |
| Striped Hyena | *Hyaena hyaena* | 44.5 | 17.8 | 8.11 | 51.2 | Pospísil *et al*., 1987a |
| Family *Canidae* |  |  |  |  |  |  |
| Hunting Dog | *Lycaon pictus* | 43.3 | 17.34 | 8.45 | 50.95 | Pospísil *et al*., 1987a |
| Grey Wolf | *Canus lupus* | 46.5 | 17.2 | 7.45 | 63.8 | Pospísil *et al*., 1987a |
| Coyote | *Canis latrans* | 49.0 | 14.7 | 7.7 | 63.4 | Gates and Goering, 1976 |
| Golden Jackal | *Canis* *aureus* | 38.4 | 12.6 | 6.28 | 60 | Aroch *et al*., 2005 |
| Asian Wild Dog | *Cuon alpinus* |  |  |  |  | Salakij *et al*., 2000 |
| Maned Wolf | *Chrysocyon brachyurus* | 40.7 | 13.1 | 5.0 | 82.1 | May-Júnior et al., 2009 |
| Crab-eating Fox | *Cerdocyon thous* | 38.1 | 12.9 | 4.27 | 89.8 | Mattoso *et al*., 2012 |
| Ranch Gray Fox | *Urocyon cinereoargenteus* | 48 | 17.0 | 10.8 |  | Benn *et al*., 1986 |
| Family *Eupleridae* |  |  |  |  |  |  |
| Fossa | *Cryptoprocta ferox* | 48.4 | 15.2 | 9.6 | 51.3 | Langer *et al*., 2013 |
| Order *Pholidota* |  |  |  |  |  |  |
| Tree Pangolin | *Manis tricuspis* | 40.4 | 10.0 | 4.2 | 97.7 | Oyewale *et al*., 1997 |
| Order *Rodentia* |  |  |  |  |  |  |
| Family *Cricetidae* |  |  |  |  |  |  |
| Hispid Cotton Rat | *Sigmodon hispidus* | 34.4 | 10.5 | 4.8 | 70.3 | Robel *et al.,* 1996 |
| Norwegian Lemming | *Lemmus lemmus* | 41.9 | 14.4 | 9.91 | 42.3 | Wiger, 1977 |
| Muskrat | *Ondatra zibethicus* | 46.6 | 16.1 | 5.95 | 78.3 | Ahlers *et al*., 2011 |
| Pine Vole | Microtus pinetorum | 40.8 | 15.0 | 11.0 | 37.5 | Harvey *et al*., 2008 |
| Family *Dipodidae* |  |  |  |  |  |  |
| Northern Birch Mouse | *Sicista betulina* | 43.8 | 14.7 | 9.85 | 45.4 | Wołk, 1985 |
| Family *Erethizontidae* |  |  |  |  |  |  |
| Brazilian Porcupine | *Coendou prehensilis* | 33.75 | 11.0 | 3.6 | 94.0 | Moreau *et al*., 2003 |
| Black-tailed Hairy Dwarf Porcupine | *Coendou melanurus* | 33.0 | 10.8 | 3.5 | 93.7 | Moreau *et al*., 2003 |
| Bristle-spined Rat or Thin-spined Porcupine | *Chaetomys subspinosus* | 33.2 | 10.3 | 3.45 | 100.8 | de Almeida Curi *et al.,* 2012 |
| Family *Muridae* |  |  |  |  |  |  |
| Algerian mouse | *Mus spretus* | 47.0 | 13.2 | 7.0 | 70.1 | Mira and da luz Mathias, 1994 |
| [Carpentarian Rock-rat](http://link.springer.com.ezproxy.lib.uwm.edu/article/10.1007/s00580-007-0694-y) | *Zyzomys palatalis* | 40 | 11.8 | 5.99 | 67.0 | Old *et al*., 2007 |
| Central Rock-rat | Zyzomys pedunculatus | 51 | 12.2 | 6.22 | 64 | Old *et al*., 2005 |
| House Mouse | *Mus musculus domesticus* | 51.2 | 13.5 | 7.13 | 67.8 | Mira and da luz Mathias, 1994 |
| Libyan Jird | *Meriones libycus* | 39.9 | 12.4 | 6.36 | 45.9 | Madjdzadeh *et al*., 2011; Alagaili *et al*., 2013 |
| Persian Jird | *Meriones persicus* |  |  | 3.87 |  | Madjdzadeh *et al*., 2011 |
| Dusky-footed Wood Rat | *Neotoma fuscipes* | 36.96 | 11.23 | 7.72 | 48.4 | Weber et al., 2002 |
| Plains Rat | Pseudomys australis | 42 | 13.3 | 7.25 | 58 | [Old](http://link.springer.com.ezproxy.lib.uwm.edu/search?facet-author=%22J.+M.+Old%22) *et al*., 2005 |
| Short-tailed Bandicoot Rat | *Nesokia indica* |  |  | 4.51 |  | Madjdzadeh *et al*., 2011 |
| Spinifex Hopping-mouse | Notomys alexis | 38 | 12.8 | 7.78 | 48.7 | Old *et al*., 2005 |
| Indian Gerbil | *Tatera indica* |  |  | 4.07 |  | Madjdzadeh *et al*., 2011 |
| Lab Rat | Rattus norvegicus |  |  | 4.78 |  | Madjdzadeh *et al*., 2011 |
| Sand Rat | Psammomys obesus | 41.15 | 11.9 | 6.72 | 61.8 | Kane *et al*., 2012 |
| Wood Mouse | *Apodemus sylvaticus* | 48.0 | 17.3 | 10.1 | 47.9 | Rogival *et al*., 2006 |
| Family *Myocastoridae* |  |  |  |  |  |  |
| Coypu | *Myocastor coypus* | 43.0 | 9.6 | 4.5 | 96.8 | Martino *et al*., 2012 |
| Family *Nesomyidae* |  |  |  |  |  |  |
| Gambian Pouched Rat | *Cricetomys gambianus* | 48.3 | 14.36 | 5.90 | 86.8 | Oyewale *et al.,* 1998 |
| [Family](http://en.wikipedia.org/wiki/Family_(biology)) *Sciuridae* |  |  |  |  |  |  |
| American Red Squirrel | *Tamiasciurus hudsonicus* | 43 | 11.5 | 8.9 | 50 | Youatt *et al*., 1961 |
| Fox Squirrel | *Sciurus niger* | 44 | 11.4 | 8.5 | 58 | Youatt *et al*., 1961 |
| Woodchuck | *Marmota monax* | 41 | 11.6 | 7.4 | 35 | Youatt *et al*., 1961 |
| Family *Thryonomyidae* |  |  |  |  |  |  |
| Greater Cane Rat | *Thryonomys swinderianus* | 41.5 | 14.2 | 8.4 | 71.9 | Opara *et al.,* 2006 |
| Order *Lagomorpha* |  |  |  |  |  |  |
| Eastern Cottontail Rabbit | *Sylvilagus floridanus* | 43 | 11.5 | 6.3 | 69 | Youatt *et al*., 1961 |
| European Brown Hare | *Lupus europaeus* | 60.0 | 20.8 | 10.0 | 60.5 | Marco *et al*., 2003 |
| Riparian Brush Rabbit | *Sylvilagus bachmani* | 37.75 | 12.1 | 5.95 | 63.7 | Black *et al*., 2009 |
| Order *Artiodactyla* |  |  |  |  |  |  |
| F[amily](http://en.wikipedia.org/wiki/Family_(biology)) *Cervidae* |  |  |  |  |  |  |
| Axis deer | *Axis axis* | 38 | 14.2 | 12.5 | 30.3 | Hawkey and Hart, 1985 |
| Barasingha | *Cervus duvauceli* | 43 | 15.3 | 8.7 | 48.8 | Hawkey and Hart, 1985 |
| Elk | *Cervus canadensis* | 67.2 | 19.2 | 11.0 | 62.0 | Pedersen and Pedersen, 1975 |
| Red Deer | *Cervus elaphus* | 48.8 | 16.3 | 9.8 | 49.5 | Shideler *et al*., 2002 |
| Fallow deer | *Dama dama* | 41.4 | 15.9 | 9.6 | 43.4 | English and Lepherd, 1981 |
| Persian fallow deer | *Dama mesopotamica* |  | 15.0 | 7.76 | 49.9 | Mohri *et* *al*., 2000 |
| Marsh deer | *Blastocerus dichotomus* | 41.0 | 14.09 | 4.75 | 48.1 | Szabó *et al*., 2005 |
| Moose | *Alces alces* | 46 | 16.1 | 6.8 | 69 | Rostal *et al*., 2012 |
| Mule Deer | *Odocoileus hemionus* | 48 | 18.2 | 13.0 | 37.0 | DelGiudice *et al*., 1990 |
| White-tailed Deer | *Odocoileus virginianus* | 32 | 11.7 | 11.96 | 26.5 | Presidente *et al*., 1973 |
| Père David’s Deer | *Elaphus davidianus* | 42 | 15.2 | 8.3 | 50.3 | Hawkey and Hart, 1985 |
| Pudú | *Pudu pudu* | 51.7 | 19.7 | 11.2 | 48.2 | Montes et al., 2004 |
| Reindeer | *Rangifer tarandus* | 44 | 17.2 | 9.29 | 47.8 | Catley *et al*., 1990. |
| Roe Deer | *Capreolus capreolus* | 44 | 16.7 | 12.42 | 35.9 | Montane *et al*., 2002 |
| Rusa Deer | *Cervus timorensis* | 35 | 13.3 | 6.2 | 58.1 | Tomkins and Jonsson, 2005 |
| Sambar Deer | *Cervus unicolor* | 42 | 13.1 | 9.6 | 44.3 | Gono, 1993 |
| Sika Deer | *Cervus nippon* | 28 | 10.7 | 9.0 | 31 | Yamanaka, 1989. |
| Eld's Deer | *Panolia eldii* | 397 | 12.3 |  |  | Nimitsuntiwong *et al*., 2000 |
| *F*[*amily*](http://en.wikipedia.org/wiki/Family_(biology)) *Bovidae* |  |  |  |  |  |  |
| Sub-family *Aepycerotinae* |  |  |  |  |  |  |
| Impala | *Aepyceros melampus* | 44.2 | 15.0 | 22.89 |  | Drevemo *et al*., 1974 |
| Sub-family *Antilopinae* |  |  |  |  |  |  |
| Erlanger’s Gazelle | *Gazella erlangeri* | 50.8 | 18.97 | 12.98 | 39.3 | Aljumaah and Hussein, 2011 |
| Goitered Gazelle | *Gazella subgutturosa* | 51.6 | 18.85 | 11.7 | 45.1 | Yaralioğlu *et al*., 2004 |
| Grant’s Gazelle | *Gazella granti* | 40.9 | 15.7 | 9.64 |  | Drevemo *et al*., 1974 |
| Speke's Gazelle | *Gazella spekei* | 46.6 | 17.5 |  |  | [Travis](http://www.ncbi.nlm.nih.gov/pubmed?term=Travis%20EK%5BAuthor%5D&cauthor=true&cauthor_uid=17312817) and [Eby,](http://www.ncbi.nlm.nih.gov/pubmed?term=Eby%20C%5BAuthor%5D&cauthor=true&cauthor_uid=17312817)  2006 |
| Thompson’s Gazelle | *Gazella thomsonii* | 44.9 | 16.7 | 10.22 |  | Drevemo *et al*., 1974 |
| Sub-family *Bovinae* |  |  |  |  |  |  |
| African Buffalo | *Syncerus caffer* | 30.5 | 11.6 | 9.38 | 36 | Mean of Drevemo *et al*., 1974; Beechler *et al*., 2009 |
| American Bison | *Bison bison* | 50 | 17.2 |  |  | Marler, 1975 |
| Greater Kudu | *Tragelaphus strepsiceros* | 42.0 | 15.5 | 7.09 | 61.3 | Pospísil *et al*., 1984a |
| Subfamily *Caprinae* |  |  |  |  |  |  |
| Spanish Ibex | *Capra pyrenaica* | 39.5 | 14.0 | 17.16 | 23 | Casas-Díaz et al., 2008 |
| Mountain Goat | *Oreamnos americanus* | 41.5 | 13.3 | 9.7 | 43.5 | Rice and Hall, 2007 |
| Rocky Mountain Bighorn Sheep | *Ovis Canadensis* | 53.1 | 18.6 | 10.1 |  | Woolf and Kradel, 1970; McDonald *et al*., 1981 |
| Stone Sheep | *Ovis dalli* | 50.6 | 18.5 | 6,842 |  | Franzmann, 1971 |
| Big horn Sheep | *Ovis canadensis* |  |  | - |  | McDonald *et al*., 1981 |
| Domestic Sheep | *Ovis aries* | 35 | 12.3 | 9.0 |  | McDonald *et al*., 1981 |
| Family *Giraffidae* |  |  |  |  |  |  |
| Giraffe | *Giraffa camelopardalis* | 43 | 14.4 | 10.15 |  | Drevemo *et al*., 1974 |
| Sub-family *Hippotraginae* |  |  |  |  |  |  |
| Roan Antelope | *Hippotragus equinus* | 38 | 13.5 | 11.5 | 35.1 | Pospísil *et al*., 1984a |
| Sable Antelope | *Hippotragus niger* | 44 | 15.6 | 15.5 | 28.8 | Pospísil *et al*., 1984a |
| Mountain Reedbuck | *Redunca fulvorufula* | 53.0 | 17.87 | 8.69 | 59.9 | Pospísil *et al*., 1984b |
| Waterbuck | *Kobus ellipsiprymnus* | 44.7 | 15.35 | 11.24 | 40.7 | Pospísil *et al*., 1984b |
| Lechwe | *Kobus leche* | 53.8 | 19.61 | 8.39 | 68.8 | Pospísil *et al*., 1984b |
| Mrs Gray’s Waterbuck | *Kobus megaceros* | 46.6 | 17.31 | 8.77 | 51.8 | Pospísil *et al*., 1984b |
| Springbok | *Antidorcas marsupialis* | 48.0 | 15.59 | 11.06 | 43.3 | Pospísil *et al*., 1984b |
| Adax Antelope | *Addax nasomaclatus* | 47.7 | 16.46 | 10.37 | 45.4 | Pospísil *et al*., 1984a |
| Gemsbok Oryx | *Oryx gazella* | 43.0 | 14.75 | 12.62 | 34.05 | Pospísil *et al*., 1984a |
| Scimitar-horned Oryx | *Oryx dammah* | 48.3 | 12.1 | 11.36 | 43.5 | Pospísil *et al*., 1984a |
| Nyala | *Tragelaphus angasii* | 48.8 | 14.87 | 9.93 | 55.1 | Pospísil *et al*., 1984a |
| Common Eland | *Taurotragus oryx* | 40.1 | 13.3 | 8.28 | 49.4 | Mean of Drevemo *et al*., 1974; Pospísil *et al*., 1984a |
| Bongo | *Tragelaphus eurycerus* | 42.0 | 10.55 | 6.44 | 65.4 | Pospísil *et al*., 1984a |
| Mountain Reedbuck | *Redunca fulvorupula* | 43.3 | 15.4 | 8.34 |  | Drevemo *et al*., 1974 |
| Blue Wildebeest | *Connochaetes taurinus* | 43.0 | 15.9 | 14.96 |  | Drevemo *et al*., 1974 |
| Coke’s Hartebeest | *Alcelaphus buselaphus* | 41.5 | 16.1 | 9.49 |  | Drevemo *et al*., 1974 |
| Topi | *Damaliscus korrigum* | 38.0 | 13.6 | 11.76 |  | Drevemo *et al*., 1974 |
| Order *Perissodactyla* |  |  |  |  |  |  |
| Feral horse | *Equus caballus* | 47.5 | 17.7 | 9.3 | 50.7 | Seal et al., 1985 |
| [Grevy's Zebra](http://en.wikipedia.org/wiki/Grevy%27s_zebra) | *Equus grevyi* | 45 | 14.3 | 9.5 | 49.1 | Pospísil *et al*., 1985 |
| Mountain Zebra | *Equus zebra* | 43 | 16.6 | 9.7 | 45 | Pospísil *et al*., 1985 |
| Ongar | *Equus hemionus* | 36.9 | 10.2 | 6.38 | 52.1 | Jani *et al*., 2004 |
| Plains Zebra | *Equus quagga* | 40 | 11.9 | 8.4 | 48.5 | Pospísil *et al*., 1985 |
| Baird's Tapir | *Tapirus bairdii* | 25.7 |  |  |  | Hernandez-Divers *et al*., 2005 |
| Sumatran Rhinoceros | *Dicerorhinus sumatrensis* | 39 | 13.2 | 5.1 |  | Andriansyah et al., 2013 |
| Black Rhinoceros | *Diceros bicornis* | 43 | 16.1 | 5.26 | 82.5 | Kock *et al*., 1990 |
| Order *Chiroptera* |  |  |  |  |  |  |
| Sub-order *Microchiroptera* |  |  |  |  |  |  |
| Family *Emballonuridae* |  |  |  |  |  |  |
| Black-bearded Tomb Bat | *Taphozous melanopogon* | 59.2 | 16.0 | 8.9 | 59.3 | Ratnasooriya *et al*., 2005 |
| Proboscis Bat | *Rhynchonycteris naso* | 62.2 |  |  |  | Schinnerl *et al*., 2011 |
| Greater sac-winged Bat | *Saccopteryx bilineata* | 54.6 |  |  |  | Schinnerl *et al*., 2011 |
| Bonda mastiff bat | *Molossus bondae* | 64.0 |  |  |  | Schinnerl *et al*., 2011 |
| Sinaloan Mastiff Bat | *Molossus sinaloae* | 65.8 |  |  |  | Schinnerl *et al*., 2011 |
| Chestnut Short-tailed Bat | *Carollia castanea* | 55.0 |  |  |  | Schinnerl *et al*., 2011 |
| Seba’s Short-tailed Bat | *Carollia perspicillata* | 58.9 |  |  |  | Schinnerl *et al*., 2011 |
| Sowell’s Short-tailed Bat | *Carollia sowelli* | 55.3 |  |  |  | Schinnerl *et al*., 2011 |
| Commissaris’s Long-tongued Bat | *Glossophaga commissarisi* | 56.4 |  |  |  | Schinnerl *et al*., 2011 |
| Hairy Big-eared Bat | *Micronycteris hirsuta* | 56.1 |  |  |  | Schinnerl *et al*., 2011 |
| Striped Hairy-nosed Bat | *Mimon crenulatum* | 55.9 |  |  |  | Schinnerl *et al*., 2011 |
| Pale Spear-nosed Bat | *Phyllostomus discolor* | 51.8 |  |  |  | Schinnerl *et al*., 2011 |
| Fringe-lipped Bat | *Trachops cirrhosus* | 51.8 |  |  |  | Schinnerl *et al*., 2011 |
| Jamaican Fruit Bat | *Artibeus jamaicensis* | 54.0 |  |  |  | Schinnerl *et al*., 2011 |
| Thomas’s Fruit-eating Bat | *Artibeus watsoni* | 57.2 |  |  |  | Schinnerl *et al*., 2011 |
| Honduran White Bat | *Ectophylla alba* | 57.2 |  |  |  | Schinnerl *et al*., 2011 |
| Elegant Myotis | *Myotis elegans* | 56.5 |  |  |  | Schinnerl *et al*., 2011 |
| Chestnut Sac-winged Bat | *Cormura brevirostris* | 56.8 |  |  |  | Schinnerl *et al*., 2011 |
| Big Naked-backed Bat | *Pteronotus gymnonotus* | 63.3 |  |  |  | Schinnerl *et al*., 2011 |
| Great Fruit-eating Bat | *Artibeus lituratus* | 53.3 |  |  |  | Schinnerl *et al*., 2011 |
| Common Vampire Bat | *Desmodus rotundus* | 57 |  |  |  | Schinnerl *et al*., 2011 |
| Underwood’s Long-tongued Bat | *Hylonycteris underwoodi* | 60.5 |  |  |  | Schinnerl *et al*., 2011 |
| Greater Spear-nosed Bat | *Phyllostomus hastatus* | 55.7 |  |  |  | Schinnerl *et al*., 2011 |
| Heller’s Broad-nosed Bat | *Platyrrhinus helleri* | 64.3 |  |  |  | Schinnerl *et al*., 2011 |
| Stripe-headed Round-eared Bat | *Tonatia saurophila* | 55.85 |  |  |  | Schinnerl *et al*., 2011 |
| Striped Yellow-eared Bat | *Vampyressa nymphaea* | 60.9 |  |  |  | Schinnerl *et al*., 2011 |
| Black Myotis | *Myotis nigricans* | 49.5 |  |  |  | Schinnerl *et al*., 2011 |
| Indian Roundleaf Bat | *Hipposideros lankadiva* | 59.2 |  | 8.9 | 70.0 | Ratnasooriya *et al*., 2005 |
| Family *Vespertilionidae* |  |  |  |  |  |  |
| Common Bent-wing Bat | *Miniopterus schreibersii* | 50.5 | 18.5 | 10.9 | 49.3 | Mean of Clark, 2004 and Ratnasooriya *et al*., 2005 |
| Gould's Wattled Bat | *Chalinolobus gouldii* | 52 | - | - | - | Clark, 2004 |
| Lesser Long-eared Bat | *Nyctophilus geoffroyi* | 54.5 | - | - | - | Clark, 2004 |
| Sub-order *Megachiroptera* |  |  |  |  |  |  |
| Family *Pteropodidae* |  |  |  |  |  |  |
| Grey-headed Flying-fox | *Pteropus poliocephalus* | 48.5 | 17.85 | 8.8 | 56.5 | mean 2 studies cited in Clark, 2004 |
| Little Red Flying -fox | *Pteropus scapulatus* | 52 | 18.15 | 10.75 | 50.5 | mean 2 studies cited in Clark, 2004 |
| Malaysian Flying-fox | *Pteropus* *vampyrus* | 44 | 14.6 | 8.88 | 49.1 | Heard and Whittier, 1997 |
| Rodriguez Island Flying-fox | *Pteropus rodricensis* | 43 | 14.2 | 7.95 | 54.4 | Heard and Whittier, 1997 |
| Small Flying-fox | *Pteropus* *hypomelanus* | 46.2 | 11.5 | 8.6 | 51.5 | Mean Heard and Whittier, 1997 and 4 studies cited in Clark, 2004 |
| Order Primates |  |  |  |  |  |  |
| Red ruffed Lemur | *Varecia rubra* | 45.7 |  |  |  | Dutton *et al.,* 2008 |
| Ring-tailed lemur | *Lemur catta* | 45.0 | - | 6.5 | - | Moresco *et al*., 2012 |
| White-Footed Tamarin | *Saguinus leucopus* | 49 | 16.1 | 6.74 | 73.6 | Fox *et al*., 2008 |
| Order *Erinaceomorpha* | *Laurasiatheria* |  |  |  |  |  |
| European Hedgehog | *Erinaceous europaeus* | 33 | 12.5 | 8.1 | 40.6 | Lewis *et al*., 2002 |
| Eastern Mole | Scalopus aquaticus | 56.4 | 19.2 | 12.6 | 46.0 | Campbell *et al*., 2010 |
| Coast Mole | Scapanus orarius | 46.8 | 17.4 | 10.5 | 42.6 | Campbell *et al*., 2010 |

**Supplementary Table 2.** Plasma lipid concentrations in individual avian and mammalian species

|  | Laying | Non-laying | Reference |
| --- | --- | --- | --- |
| **Triglyceride g dL^-1^** |  |  |  |
| **Birds** |  |  |  |
| Chicken (*Gallus gallus*) | 2.268 + (5) 0.516 | 0.408 + (3) 0.063 | Neill et al., 1977; Hagan et al., 1984; Lien et al., 2001;  Peebles et al., 2004; Lv et al., 2018 |
| Duck (*Anas platyrhynchos*) | 1.706 | 0.162 | Hermier et al., 2003;  Lien et al., 1999;  Liu et al., 2011 |
| Bar-tailed godwit (*Limosa lapponica*) |  | 0.253 | Landys et al., 2005 |
| European starling (*Sturnus vulgaris*) |  | 0.402 | Juráni et al., 2004 |
| Eurasian tree sparrow (*Passer montanus*) |  | 0.218 | Li et al., 2019 |
| Hispaniolan amazon parrots (*Amazona ventralis*) |  | 0.0845 | Robertson et al., 2020 |
| King penguin (*Aptenodytes patagonicus*) |  | 0.068 | Bernard et al., 2002 |
| Least sandpiper (*Calidris minutilla*) |  | 0.193 | Thomas and Swanson, 2013 |
| Monk parakeet (*Myiopsitta monachus*) |  | 0.386 | Belcher et al., 2014 |
| Mynah (*Acridotheres tristis*). |  | 0.127 | Jahantigh et al., 2019 |
| Orange-winged Amazon parrots (*Amazona amazonica*). |  | 0.0136 | Vergneau-Grosset et al., 2016 |
| Pectoral sandpiper (*Calidris melanotos*) |  | 0.117 | Thomas and Swanson, 2013 |
| Semipalmated sandpipers (*Calidris pusilla*) |  | 0.204 | Lyons et al., 2008; Thomas and Swanson, 2013 |
| Turkey (*Meleagris gallopavo*) | 1.581 | 0.207 | Bacon et al., 1974; Bacon, 1981; Mossab et al., 2002; Riot et al., 2015; Diot et al., 2015 |
| White-throated sparrow (*Zonotrichia albicollis*) |  | 0.228 | Smith et al., 2009 |
| Zebra finch (*Taeniopygia guttata*) | 1.805 | 0.722 | Salvante et al., 2007 |
| **Mammals** |  |  |  |
| African green monkey (*Chlorocebus aethiops*) |  | 0.054 | Yin et al., 2012 |
| Armadillo (species not specified) |  | 0.0529 | Maldonado et al., 2002 |
| Black rhinoceros (*Diceros bicornis*) |  | 0.0059 | Leat et al., 1979 |
| Cat (*Felis catus*) |  | 0.0167 | Maldonado et al., 2002; Kaabia et al., 2018 |
| Cattle (*Bos indicus*) |  | 0.099 | Kaabia et al., 2018 |
| Common zebra (*Equus quagga*) |  | 0.0096 | Leat et al., 1979 |
| Cynomolgus monkey (*Macaca fascicularis*) |  | 0.058 | Yin et al., 2012; Kaabia et al., 2018 |
| Dog (*Canis lupus*) |  | 0.052 | Maldonado et al., 2002; Yin et al., 2012; Kaabia et al., 2018 |
| Donkey (*Equus africanus*) |  | 0.0093 | Leat et al., 1979 |
| Ferret (*Mustela putorius*) |  | 0.0185 | Marini et al., 2017 |
| Hamster (species not specified) |  | 0.156 | Yin et al., 2012; Kaabia et al., 2018 |
| Horse (*Equus ferus*) |  | 0.024 | Leat et al., 1979; Kaabia et al., 2018 |
| Human (*Homo sapiens*) |  | 0.062 | Kaabia et al., 2018 |
| Indian rhinoceros (*Rhinoceros* *unicornis*) |  | 0.0043 | Leat et al., 1979 |
| Malayan tapir (*Tapirus indicus*) |  | 0.0273 | Leat et al., 1979 |
| Marmoset (species not specified) |  | 0.363 | Yin et al., 2012 |
| Mountain zebra (*Equus zebra*) |  | 0.0174 | Leat et al., 1979 |
| Mouse (*Mus musculus*) |  | 0.145 | Maldonado et al., 2002; Kaabia et al., 2018 |
| Onager (*Equus hemionus*) |  | 0.0252 | Leat et al., 1979 |
| Pig (*Sus scrofa*) |  | 0.037 | Yin et al., 2012; Kaabia et al., 2018 |
| Przewalski horse (*Equus przewalskii*) |  | 0.015 | Leat et al., 1979 |
| Rabbit (*Oryctolagus cuniculus*) |  | 0.047 | Yin et al., 2012 |
| Rat (*Rattus norvegicus*) |  | 0.066 | Siques et al., 2014; Kaabia et al., 2018 |
| Rhesus monkey (*Macaca mulatta*) |  | 0.042 | Yin et al., 2012 |
| White rhinoceros (*Ceratotherium simum*) |  | 0.0157 | Leat et al., 1979 |
|  |  |  |  |
| **Cholesterol g dL^-1^** |  |  |  |
| **Birds** |  |  |  |
| Chicken (*Gallus gallus*) | 0.172 + (5) 6.4 | 0.101 + (3) 0.0023 | Neill et al., 1977; Hagen et al., 1984; Lien et al., 2001; Peebles et al., 2004; Lv et al., 2018 |
| Turkey (*Meleagris gallopavo*) | 0.180 | 0.191 | Mossab et al., 2002; Diot et al., 2015 |
| Duck (*Anas platyrhynchos*) | 0.183 | 0.146 | Lien et al., 1999;  Ismoyowati and Sumarmono, 2010; Liu et al., 2011 |
| European starling (*Sturnus vulgaris*) |  | 0.199 | Juráni et al., 2004 |
| Eurasian tree sparrow (*Passer montanus*) |  | 0.218 | Li et al., 2019 |
| Hispaniolan amazon parrots (*Amazona ventralis*) |  | 0.384 | Robertson et al., 2020 |
| Monk parakeet (*Myiopsitta monachus*) |  | 0.310 | Belcher et al., 2014 |
| Mynah (*Acridotheres tristis*). |  | 0.189 | Jahantigh et al., 2019 |
| Orange-winged Amazon parrots (*Amazona amazonica*). |  | 0.307 | Vergneau-Grosset et al., 2016 |
|  |  |  | |
| **Mammals** |  |  |  |
| African green monkey (*Chlorocebus aethiops*) |  | 0.134 | Yin et al., 2012 |
| Bear (*Ursus arctos*) [mean of European and grizzly] |  | 0.279 | Hissa, 1997; Rivet et al., 2017 |
| Black rhinoceros (*Diceros bicornis*) |  | 0.1173 | Leat et al., 1979 |
| Cat (*Felis catus*) |  | 0.237 | Morris and Courtice, 1955; Yin et al., 2012; Kaabia et al., 2018 |
| Cattle (*Bos indicus*) |  | 0.081 | Morris and Courtice, 1955; Kaabia et al., 2018 |
| Common zebra (*Equus quagga*) |  | 0.2407 | Leat et al., 1979 |
| Cynomolgus monkey (*Macaca fascicularis*) |  | 0.132 | Yin et al., 2012; Kaabia et al., 2018 |
| Dog (*Canis lupus*) |  | 0.173 | Leat et al., 1979; Kaabia et al., 2018 |
| Donkey (*Equus africanus*) |  | 0.1848 | Leat et al., 1979 |
| Goat (*Capra aegagrus*) |  | 0.034 | Morris and Courtice, 1955 |
| Ferret (*Mustela putorius*) |  | 0.174 | Marini et al., 2017 |
| Guinea pig (*Cavia porcellus*) |  | 0.050 | Morris and Courtice, 1955 |
| Hamster (species not specified) |  | 0.178 | Yin et al., 2012; Kaabia et al., 2018 |
| Horse (*Equus ferus*) |  | 0.142 | Morris and Courtice, 1955; Leat et al., 1979 |
| Human (*Homo sapiens*) |  | 0.207 | Morris and Courtice, 1955; Kaabia et al., 2018 |
| Indian rhinoceros (*Rhinoceros* *unicornis*) |  | 0.0982 | Leat et al., 1979 |
| Malayan tapir (*Tapirus indicus*) |  | 0.3003 | Leat et al., 1979 |
| Marmoset (species not specified) |  | 0.161 | Yin et al., 2012 |
| Mountain zebra (*Equus zebra*) |  | 0.1827 | Leat et al., 1979 |
| Mouse (*Mus musculus*) |  | 0.116 | Morris and Courtice, 1955; Kaabia et al., 2018 |
| Onager (*Equus hemionus*) |  | 0.1481 | Leat et al., 1979 |
| Pig (*Sus scrofa*) |  | 0.114 | Yin et al., 2012; Kaabia et al., 2018 |
| Przewalski horse (*Equus przewalskii*) |  | 0.1625 | Leat et al., 1979 |
| Rabbit (*Oryctolagus cuniculus*) |  | 0.037 | Morris and Courtice, 1955; Yin et al., 2012 |
| Rat (*Rattus norvegicus*) |  | 0.113 | Morris and Courtice, 1955 |
| Rhesus monkey (*Macaca mulatta*) |  | 0.120 | Yin et al., 2012 |
| Sheep (*Ovis aries*) |  | 0.064 | Morris and Courtice, 1955 |
| White rhinoceros (*Ceratotherium simum*) |  | 0.182 | Leat et al., 1979 |
|  |  |  |  |
| **Phospholipids g dL^-1^** |  |  |  |
| **Birds** |  |  |  |
| Chicken (*Gallus gallus*) | 0.453 + (3) 0.247 | 0.141 + (3) 0.040 | Neill et al., 1977; Hagan et al., 1984 |
| Duck (*Anas platyrhynchos*) | 0.182 | 0.208 | Lien et al., 1999; Hermier et al., 2003; Diop et al., 2015 |
| Muscovy duck (*Cairina moschata*) |  | 0.228 | Hermier et al., 2003 |
| Semipalmated sandpipers (*Calidris pusilla*) |  | 0.752 | Lyons et al., 2008 |
| Turkey (*Meleagris gallopavo*) | 0.507 | 0.146 | Bacon, 1981; Mossab et al., 2002; Diot et al., 2015 |
|  |  |  |  |
| **Mammals** |  |  |  |
| Black rhinoceros (*Diceros bicornis*) |  | 0.0368 | Leat et al., 1979 |
| Cat (*Felis catus*) |  | 0.183 | Morris and Courtice, 1955; Yin et al., 2012; Kaabia et al., 2018 |
| Cattle (*Bos indicus*) |  | 0.089 | Kaabia et al., 2018 |
| Common zebra (*Equus quagga*) |  | 0.2618 | Leat et al., 1979 |
| Dog (*Canis lupus*) |  | 0.231 | Kaabia et al., 2018 |
| Donkey (*Equus africanus*) |  | 0.182 | Leat et al., 1979 |
| Goat (*Capra aegagrus*) |  | 0.026 | Morris and Courtice, 1955 |
| Guinea pig (*Cavia porcellus*) |  | 0.021 | Morris and Courtice, 1955 |
| Horse (*Equus ferus*) |  | 0.105 | Morris and Courtice, 1955; Leat et al., 1979 |
| Human (*Homo sapiens*) |  | 0.231 | Kaabia et al., 2018 |
| Indian rhinoceros (*Rhinoceros* *unicornis*) |  | 0.0608 | Leat et al., 1979 |
| Malayan tapir (*Tapirus indicus*) |  | 0.1881 | Leat et al., 1979 |
| Mountain zebra (*Equus zebra*) |  | 0.1908 | Leat et al., 1979 |
| Mouse (*Mus musculus*) |  | 0.261 | Kaabia et al., 2018 |
| Onager (*Equus hemionus*) |  | 0.1733 | Leat et al., 1979 |
| Pig (*Sus scrofa*) |  | 0.129 | Kaabia et al., 2018 |
| Przewalski horse (*Equus przewalskii*) |  | 0.1341 | Leat et al., 1979 |
| Rabbit (*Oryctolagus cuniculus*) |  | 0.032 | Morris and Courtice, 1955; Yin et al., 2012 |
| Rat (*Rattus norvegicus*) |  | 0.223 | Kaabia et al., 2018 |
| Sheep (*Ovis aries*) |  | 0.041 | Morris and Courtice, 1955 |
| White rhinoceros (*Ceratotherium simum*) |  | 0.0631 | Leat et al., 1979 |
|  |  |  |  |
| **Total lipids** **g dL^-1^** |  |  |  |
| **Birds** |  |  |  |
| Black-tailed godwit *Limosa limosa* |  | 0.175 | Araújo et al., 2019 |
| Chicken (*Gallus gallus)* | 2.899 + (3) 0.842 | 0.408 + (3) 0.063 | Neill et al., 1977; Hagen et al., 1984; Lien et al., 2001 |
| Turkey (*Meleagris gallopavo*) | 2.304 |  | Diot et al., 2015 |
| Duck (*Anas platyrhynchos*) | 1.745 | 0.445 | Lien et al., 1999 |
| European starling (*Sturnus vulgaris*) |  | 0.883 | Juráni et al., 2004 |
| Gentoo penguin (*Pygoscelis papua*) |  | 0.83 | Ibañez et al., 2015 |
| Adélie penguin (*Pygoscelis adeliae*) |  | 0.79 | Ibañez et al., 2015 |
| Chinstrap penguin (*Pygoscelis antarcticus*) |  | 0.83 | Ibañez et al., 2015 |
|  |  |  |  |
| **Mammals** |  |  | |
| Cynomolgus monkey (*Macaca fascicularis*) |  | 0.268 | Kaabia et al., 2018 |
| Mouse (*Mus musculus*) |  | 0.507 | Kaabia et al., 2018 |
| Rat (*Rattus norvegicus*) |  | 0.42 | Kaabia et al., 2018 |
| Hamster (species not specified) |  | 0.522 | Kaabia et al., 2018 |
| Pig (*Sus scrofa*) |  | 0.172 | Kaabia et al., 2018 |
| Cattle (*Bos indicus*) |  | 0.199 | Kaabia et al., 2018 |
| Dog (*Canis lupus*) |  | 0.703 | Kaabia et al., 2018 |
| Horse (*Equus ferus*) |  | 0.339 | Leat et al., 1979; Kaabia et al., 2018 |
| Human (*Homo sapiens*) |  | 0.293 | Kaabia et al., 2018 |
| Przewalski horse (*Equus przewalskii*) |  | 0.312 | Leat et al., 1979 |
| Mountain zebra (*Equus zebra*) |  | 0.391 | Leat et al., 1979 |
| Common zebra (*Equus quagga*) |  | 0.512 | Leat et al., 1979 |
| Onager (*Equus hemionus*) |  | 0.347 | Leat et al., 1979 |
| Donkey (*Equus africanus*) |  | 0.376 | Leat et al., 1979 |
| White rhinoceros (*Ceratotherium simum*) |  | 0.261 | Leat et al., 1979 |
| Black rhinoceros (*Diceros bicornis*) |  | 0.160 | Leat et al., 1979 |
| Indian rhinoceros (*Rhinoceros* *unicornis*) |  | 0.163 | Leat et al., 1979 |
| Malayan tapir (*Tapirus indicus*) |  | 0.516 | Leat et al., 1979 |

**Supplementary Table 3.** Plasma/serum concentrations of total proteins and albumen in individual mammalian species

| Species | Concentrations in g dL^-1^ | | Reference |
| --- | --- | --- | --- |
|  | Total protein | Albumen |  |
| African buffalo (*Syncerus caffer*) | 8.37 | 3.92 | Couch et al., 2017 |
| Agoutis (*Dasyprocta leporina*) | 5.99 | 5.65 | Jones et al., 2019 |
| Anubis baboon (*Papio Anubis*) | 7.60 | 3.2 | Ogunro et al., 2019 |
| Brown hyenas (*Parahyaena brunnea*) | 5.39 | 2.70 | Wiesel et al., 2018 |
| California mouse (*Peromyscus* *californicus*) | 6.12 | 3.21 | Wiedmeyer et al., 2014 |
| Cat | 7.09 | 3.65 | Morris and Curtice, 1955 |
| Cattle | 7.58 | 3.69 | Morris and Curtice, 1955 |
| Chital deer (*Axis axis*) | 6.70 | 3.59 | Chapple et al., 1991 |
| Common bottlenose dolphins (*Tursiops truncatus*) | 5.1 | 3.8 | Schwacke et al., 2009 |
| Crab-eating Fox (*Cerdocyon thous*) | 6.50 | 2.98 | Mattoso et al., 2012 |
| Cynomolgus monkeys | 7.4 | 4.4 | Matsuzawa et al., 1993 |
| Deer mouse (*Peromyscus* *maniculatus*) | 5.67 | 3.08 | Wiedmeyer et al., 2014 |
| Dog (*Canis lupus*) | 5.90 | 3.47 | Morris and Curtice, 1955;  Matsuzawa et al., 1993 |
| Eurasian beaver (*Castor fiber*) | 6.49 | 3.64 | Girling et al., 2015 |
| Ferret (*Mustela putorius*) | 5.9 | 3.75 | Marini et al., 2017 |
| Goat | 7.66 | 4.09 | Morris and Curtice, 1955 |
| Green monkey (*Cercocebus sebaeus*) | 7.55 | 3.00 | Ogunro et al., 2019 |
| Guinea pig | 4.71 | 3.07 | Morris and Curtice, 1955 |
| Harbor seal (*Phoca vitulina*) | 5.6 | 3.5 | Greif et al., 2010 |
| Horse | 7.41 | 3.33 | Morris and Curtice, 1955 |
| Howler monkey (*Alouatta pigra*) | 7.27 | 4.37 | Canales-Espinosa et al., 2020 |
| Human | 6.85 | 4.59 | Morris and Curtice, 1955 |
| Island fox (*Urocyon littoralis*) | 6.65 | 3.0 | Inoue *et al*., 2012 |
| Libyan jird (*Meriones libycus*) | 6.11 | 3.33 | Alagaili et al., 2013 |
| Mona monkey (*Cercopithecus mona*) | 8.15 | 3.4 | Ogunro et al., 2019 |
| Muskox (*Ovibos moschatus*) | 6.26 | 3.71 | Harmes et al., 20 |
| Mouse | 5.66 | 2.77 | Morris and Curtice, 1955 |
| Nutria (*Myocastor coypus*) | 4.59 | 2.98 | Němeček et al., 2019 |
| Oldfield mouse (*Peromyscus* *polionotus*) | 7.15 | 3.90 | Wiedmeyer et al., 2014 |
| Patas monkey *(Erythrocebus patas)* | 7.65 | 3.05 | Ogunro et al., 2019 |
| Rabbit | 5.64 | 3.56 | Morris and Curtice, 1955 |
| Red deer (Cervus elaphus) | 6.5 | 3.86 | Marco and Lavín, 1999 |
| Reindeer (*Rangifer tarandus*) | 6.5 | 3.86 | Milner et al., 2003 |
| Rat | 5.94 | 3.3.5 | Morris and Curtice, 1955;  Matsuzawa et al., 1993 |
| Sheep | 7.48 | 3.64 | Morris and Curtice, 1955 |
| Silver fox | 6.8 |  | Zhan et al., 1991 |
| Spanish ibex (*Capra pyrenaica*) | 7.20 | 4.75 | Pérez et al., 2003 |
| Spotted dolphins (*Stenella attenuata*) | 7.2 | 3.7 | St. Aubin et al., 2011 |
| White-nosed coati (*Nasua narica*) | 7.04 | 3.00 | Rovirosa-Hernández et al., 2012 |
| White-footed mouse (*Peromyscus leucopus*) | 6.52 | 3.41 | Wiedmeyer et al., 2014 |
| White-tailed deer (*Odocoileus virginianus*) | 6.3 | 3.3 | Presidente et al., 1973 |
| **Means + (n=) SEM** | **6.59 + (41) 0.140** | **3.59 + (40) 0.0886** |  |

**Supplementary Table 4.**  Hematocrit and hemoglobin concentrations in sexually mature and sexually immature females or male birds

|  | Sexually immature females or males | Mature laying hens | Reference |
| --- | --- | --- | --- |
| **Hematocrit/PCV** |  |  |  |
| American kestrel (*Falco sparverius)* | 52.2 | 45.3 | Hunter and Powers, 1980 |
| Brown-headed cowbird (*Molothrus ater*) | 55.8 | 51.8 | Keys et al., 1986 |
| Chicken (*Gallus gallus*) | 37.3 | 28.3 | Medway and Kare, 1958; Sturkie, 1986; Schaal et al., 2016 |
| Common pheasant (*Phasianus colchicus*) | 38 | 33 | Hrabčáková et al., 2014 |
| Great tit (*Parus major*) | 48.1 | 47.1 | Horak et al., 1998 |
| Green-rumped parrotlet (*Forpus passerinus*) | 55.6 | 48.6 | Sheridan et al., 2004 |
| Guinea fowl (*Numida meleagris*) | 37.4 | 36.7 | Fallaw et al., 1976 |
| Mallard (*Anas platyrhynchos*) | 49 | 40 | Shave and Howard, 1976 |
| Muscovy duck (*Cairina moschata*) | 46.0 | 41.5 | Okeudo et al., 2003 |
| Pigeon (*Columba livia*) | 54.4 | 43.1 | Gayathri et al., 2006 |
| White-crowned Sparrow (*Zonotrichia leucophrys*) | 56.2 | 41.9 | Morton, 1994 |
| Zebra finch (*Taeniopygia guttata*) | 51.6 | 47.9 | Wagner et al., 2008 |
|  |  |  |  |
| **Hemoglobin** |  |  |  |
| Chicken (*Gallus gallus*) | 8.1 | 7.98 | Schaal et al., 2016 |
| Common pheasant (*Phasianus colchicus*) | 11.8 | 10.8 | Hrabčáková et al., 2014 |
| Green-rumped parrotlet (*Forpus passerinus*) | 14.9 | 15.4 | Sheridan et al., 2004 |
| Guinea fowl (*Numida meleagris*) | 12.5 | 13.4 | Fallaw et al., 1976 |
| Mallard (*Anas platyrhynchos*) | 15.0 | 11.9 | Shave and Howard, 1976 |
| Muscovy duck (*Cairina moschata*) | 15.7 | 14.2 | Okeudo et al., 2003 |
| Zebra finches (*Taeniopygia guttata*) | 15.57 | 14.33 | Wagner et al., 2008 |

**Supplementary Table 5.**  Relative ovary and oviduct weights in sexually mature and immature female birds

| Species | Sexually immature females | Sexually mature females | Reference |
| --- | --- | --- | --- |
| **BIRDS** |  |  |  |
| **Ovary** |  |  |  |
| American golden plover (*Pluvialis dominic*a) | 0.247 | 2.082 | Johnson, 1973 |
| Bank swallow (*Riparia riparia*) | 0.235 | 2.90 | Peterson, 1955 |
| Chicken (*Gallus gallus*) | 0.046 | 2.42 | From Table 5 |
| Duck (*Anas platyrhynchos*) | 0.52 | 2.47 | Krapu, 1986; Cui et al., 2019 |
| Japanese quail (*Coturnix japonica*) |  | 4.37 | Arora and Samples, 2011 |
| Jungle crow (*Corvus macrorhynchos*) | 0.008 | 1.37 | Islam et al., 2010 |
| Ostrich (*Struthio camelus*) |  | 0.844 | Olivier et al., 2009 |
| Pied flycatcher (*Ficedula hypoleuc*a) | 0.0146 | 0.304 | Silverin, 1980 |
| Pigeon (*Columba livia*) |  | 1.92 | Hegde, 2008 |
| Pink-backed pelican (*Pelicanus rufescens*) | 0.0398 | 0.304 | Din and Eltringham, 1977 |
| Ruddy duck (*Oxyura jamaicensis*) | 0.0344 | 2.035 | Tome, 1984 |
| Starling (*Sturnus vulgaris*) |  | 0.46 | Ricklefs, 1974 |
| Turkey (*Meleagris gallopavo*) | 0.057 | 1.49 | Lilburn and Nestor, 1993; Melnychuk et al., 1997 |
| White crowned sparrow (*Zonotrichia leucophrys)* | 0.0138 | 1.23 | King et al., 1966 |
| Zebra finch (*Taeniopygia guttata*) | 0.0569 | 0.21 | Sossinka, 1980 |
|  |  |  |  |
|  | | | |
| **Oviduct** |  |  |  |
| American golden plover (*Pluvialis dominic*a) | 0.082 | 0.951 | Johnson, 1973 |
| Bank swallow (*Riparia riparia*) | 0.810 | 8.78 | Peterson, 1955 |
| Chicken (*Gallus gallus*) | 0.046 | 2.84 | From Table 5 |
| Duck/mallard (*Anas platyrhynchos*) | 1.125 | 2.37 | Krapu, 1986; Cui et al., 2019 |
| Japanese quail (*Coturnix japonica*) |  | 5.17 | Arora and Samples, 2011 |
| Ostrich (*Struthio camelus*) |  | 0.844 | Olivier et al., 2009 |
| Pied flycatcher (*Ficedula hypoleuc*a) | 0.104 | 7.04 | Silverin, 1980 |
| Pink-backed pelican (*Pelicanus rufescens*) | 0.0174 | 0.217 | Din and Eltringham, 1977 |
| Ruddy duck (*Oxyura jamaicensis*) | 0.155 | 1.042 | Tome, 1984 |
| Starling (*Sturnus vulgaris*) |  | 5.32 | Ricklefs, 1974 |
| Turkey (*Meleagris gallopavo*) | 0.029 | 0.905 | Lilburn and Nestor, 1993; Melnychuk et al., 1997 |
| White crowned sparrow (*Zonotrichia leucophrys)* | 0.491 | 2.943 | King et al., 1966 |

**Supplementary Table 6.**  Relative weights of the ovary and uterus (non-gravid) in mature mammals

| **Ovaries** |  |  |
| --- | --- | --- |
| Brown rat (*Rattus norvegicus*) | 0.0710 | Sun et al., 2016 |
| Cattle (*Bos taurus*) | 0.00237 | Kouamo et al., 2014 |
| Cynomolgus macaque (*Macaca fascicularis*) | 0.0291 | Buse et al., 2008 |
| Estuarine dolphin (*Sotalia guianensis*) | 0.010 | Weber Rosa et al., 2002 |
| European hedgehog (*Erinaceous europaeus*) | 0.0111 | Deanesly, 1934 |
| Goat (*Capra aegagrus*) | 0.00136 | Islam et al., 2007 |
| Grey wolf (*Canis lupus*) | 0.00256 | Liu et al., 2010 |
| Horse (*Equus ferus*) | 0.0221 | Ono et al., 2015 |
| Pig (*Sus scrofa*) | 0.00252 | Oxender et al., 1979; Brüssow et al., 2004 |
| Sheep (*Ovis aries*) | 0.00225 | Kennedy et al., 1974 |
| Squirrel monkey (*Saimiri sciureus*) | 0.076 | Bennett, 1967 |
| White-tailed deer (*Odocoileus virginianus*) | 0.00185 | Gastal et al., 2017 |
|  |  |  |
| **Uterus (non-gravid)** |  |  |
| Brown rat (*Rattus norvegicus*) | 0.152 | Sun et al., 2016 |
| European hedgehog (*Erinaceous europaeus*) | 0.0602 | Deanesly, 1934 |
| Pig (*Sus scrofa*) | 0.214 | Brüssow et al., 2004 |
| Sheep (*Ovis aries*) | 0.0173 | Kennedy et al., 1974 |
| White-tailed deer (*Odocoileus virginianus*) | 0.058 | Gastal et al., 2017 |

**Supplementary Table 7.**  Relative organ weights in wild birds

| Species | B. wt. | Brain | Liver | Heart | Kidney | Lung | Spleen | Stomach & small intestine | Stomach | Small intestine | Reference |
| --- | --- | --- | --- | --- | --- | --- | --- | --- | --- | --- | --- |
| **Accipitriformes** |  |  |  |  |  |  |  |  |  |  |  |
| African White-backed Vulture Pseudogyps africanus | 5270 | 0.372 | 1.33 | 0.718 | 0.679 |  |  |  |  |  | 1 |
| African fish eagle *Haliaeetus vocifer* | 3500 | 0.369 | 1.32 | 0.921 | 0.527 | 1.352 |  | 4.286 |  |  | 1 |
| Buzzard *Buteo buteo* | 704 | 1.416 | 2.05 | 0.86 | 0.761 | 0.832 |  | 2.47 | 1.48 | 0.99 | 1,2 |
| Goshawk *Accipiter gentilis* | 1110 |  | 1.65 | 0.86 |  |  |  |  |  |  | 2 |
| Red Kite *Milvus milvus* | 1040 |  | 1.13 |  |  |  |  |  |  |  | 2 |
| Red-tailed hawk *Buteo jamaicensis* | 1029 | 0.974 | 1.37 | 0.674 | 0.668 | 0.901 |  | 1.786 |  |  | 2 |
| Sharp-shinned hawk *Accipiter striatus* | 520 | 1.096 |  | 0.712 |  | 1.481 |  |  |  |  | 1 |
| Sparrowhawk *Accipiter risus* | 220 |  | 2.63 | 1.12 | 0.81 |  |  | 1.58 | 0.95 | 0.63 | 2 |
| Tawny eagle *Aquila rapax* | 2244 | 0.641 | 1.92 | 0.614 | 0.548 | 1.284 |  | 5.714 |  |  | 1 |
| Western marsh harrier | 918 |  |  |  | 0.632 |  |  |  |  |  | 21 |
| White-backed vulture (*Gyps africanus*) | 5270 | 0.372 | 1.33 | 0.718 | 0.679 |  |  |  |  |  | 1 |
| Turkey buzzard *Cathartes aura* | 494 | 1.883 | 4.25 | 2.073 | 2.047 | 2.982 |  | 34.41 |  |  | 1 |
| **Anseriformes** |  |  |  |  |  |  |  |  |  |  |  |
| Black-bellied whistling duck *(Dendrocygna autumnalis)* | 360 | 0.890 |  |  |  |  | 0.0468 |  |  |  | 9 |
| Brazilian teal *(Amazonetta brasiliensis)* | 580 |  |  |  |  |  | 0.0431 |  |  |  | 9 |
| Cackling goose *Branta hutchinsii* | 1426 |  | 1.95 |  |  |  |  |  |  |  | 18 |
| Egyptian goose *Alopochen aegyptiaca* | 1935 | 0.395 | 1.773 | 0.957 | 0.504 | 1.819 |  |  |  |  | 1 |
| Fulvous whistling duck *Dendrocygna bicolor* | 730 |  |  |  |  |  | 0.0822 |  |  |  | 9 |
| Green-winged teal *Anas carolinensis* | 305 | 1.022 | 2.679 | 0.822 | 1.018 | 3.022 |  | 11.10 |  |  | 9 |
| Greater scaup *Aythya marila* | 787 | 0.608 | 2.922 | 1.017 | 1.199 | 2.288 |  | 12.60 |  |  | 1/19 |
| Lesser scaup *Aythya affinis* | 1041 |  | 5.60 | 1.29 | 1.74 | 1.69 |  |  |  |  | 1/19 |
| Mallard *Anas platyrhynchos* | 1220 | 0.481 | 2.32 | 1.13 | 0.63 |  | 0.062 | 8.82 |  |  | 21- 26, 29 |
| Muscovy duck (*Cairina moschata*) | 4420 |  | 1.49 |  |  |  |  |  |  |  |  |
| Pintail *Anas acuta* | 670 | 0.679 | 4.527 | 1.24 | 1.245 | 2.557 | 0.133 | 14.84 |  |  | 1/19 |
| Red-breasted merganser *Mergus serrator* | 770 | 0.687 | 2.568 | 1.430 | 1.258 | 2.358 | 0.0286 | 6.013 |  |  | 1/19 |
| Rosy-billed pochard *Netta peposaca* | 1100 |  |  |  |  |  | 0.0636 |  |  |  | 9 |
| White-faced whistling duck *Dendrocygna viduata* | 590 |  |  |  |  |  | 0.0729 |  |  |  | 9 |
| **Apodiformes** |  |  |  |  |  |  |  |  |  |  |  |
| Rufous-tailed hummingbird *Amazilia tzacatl* | 5.975 | 4.5 | 3.5955 | 1.68 | 0.9975 | 1.981 |  |  |  | 0.37 | 1, 20 |
| Ruby-throated Hummingbird *Archilochus colubris* | 2.7 | 2.66 | 5.15 | 2.53 | 1.25 |  |  |  | 0.54 | 1.93 | 20 |
| White-necked jacobin *Florisuga mellivora* | 7.0 | 2.18 | 2.58 | 2.65 | 1.30 |  |  |  |  |  | 20 |
| **Bucerotiformes** |  |  |  |  |  |  |  |  |  |  |  |
| Southern ground hornbill *Bucorvus leadbeateri* | 2150 | 1.221 | 2.678 | 2.63 | 1.084 | 2.431 |  | 12.093 |  |  | 1 |
| **Charadriiformes** |  |  |  |  |  |  |  |  |  |  |  |
| Bonaparte’s gull *Chroicocephalus philadelphia* | 205 | 1.215 | 4.449 | 1.678 | 1.561 | 0.486 |  | 10.51 |  |  | 1 |
| European herring gull *Larus argentatus* | 535 | 0.950 | 5.108 | 0.979 |  |  |  |  |  |  | 1 |
| Ring-billed gull *Larus delawarensis* | 720 | 1.211 | 3.265 | 1.021 | 1.035 | 1.268 |  | 21.39 |  |  | 1 |
| Little auk/dovekie *Alle alle* | 103 | 1.874 | 3.320 | 1.204 | 2.029 | 1.612 |  | 6.476 |  |  | 1 |
| Sooty shearwater *Ardenna grisea* | 268 | 1.123 | 3.743 | 0.918 | 1.206 | 0.914 |  | 6.794 |  |  | 1 |
| **Ciconiiformes** |  |  |  |  |  |  |  |  |  |  |  |
| Abdim's stork *Ciconia abdimii* | 950 | 0.768 | 2.941 | 0.884 | 0.926 | 1.08 |  |  |  |  | 1 |
| White stork *Ciconia ciconia* | 3342 | 0.479 | 1.981 | 0.911 | 0.689 | 0.910 |  |  |  |  | 1 |
| **Columbiformes** |  |  |  |  |  |  |  |  |  |  |  |
| Mourning dove *Zenaida macroura* | 122.8 | 0.83 | 2.38 | 1.32 | 0.83 |  |  |  | 3.65 | 0.91 | 20 |
| Pigeon *Columba livia* | 264.5 | 0.940 |  | 1.799 |  |  |  |  |  |  | 1 |
| Ruddy ground dove *Columbina talpacoti* | 37.9 | 1.41 | 2.08 | 0.95 | 0.56 |  |  |  | 5.19 | 1.07 | 20 |
| White-tipped dove *Leptotila verreauxi* | 132.1 | 0.84 | 2.73 | 1.37 | 0.54 |  |  |  | 7.47 | 1.07 | 20 |
| **Falconiformes** |  |  |  |  |  |  |  |  |  |  |  |
| American kestrel *Falco sparverius* | 112 | 2.241 | 2.25 | 1.00 | 0.991 | 1.357 |  | 6.634 |  |  | 1 |
| Kestrel *Falco tinnunculus* | 180 |  | 3.24 | 1.13 | 1.14 |  |  | 3.37 | 2.62 | 0.75 | 2 |
| Merlin *Falco columbarius* | 190 |  | 3.24 | 1.10 | 1.10 |  |  | 2.12 | 1.56 | 0.56 | 2 |
| Peregrine *Falco peregrinus* | 760 |  | 2.76 | 1.81 | 0.86 |  |  | 1.77 | 1.24 | 0.53 | 2 |
| **Galliformes** |  |  |  |  |  |  |  |  |  |  |  |
| Bobwhite quail *Colinus* *virginianus* | 187 |  | 1.64 | 0.49 | 0.68 | 0.50 | 0.021 |  |  |  | 10 |
| Crested francolin *Dendroperdix sephaena* | 208 | 0.721 | 4.159 | 0.702 | 1.298 |  |  |  |  |  | 1 |
| European quail *Coturnix coturnix* | 167 |  | 1.87 |  |  |  |  |  |  |  | 27 |
| Grey partridge *Perdix perdix* | 353 |  | 2.0 | 0.9 |  |  |  |  |  |  | 11 |
| Helmeted guineafowl *Numida meleagris* | 1620 | 0.259 | 2.0825 | 0.664 | 0.509 |  |  | 1.795 |  |  | 1, 12 |
| Japanese quail *Coturnix japonica* | 173.5 |  | 2.4 | 1.0 |  |  |  |  | 2.1 |  | 28 |
| Jungle fowl *Gallus gallus* | 1809 | 0.28 | 2.01 | 0.641 |  | 0.746 | 0.133 |  |  |  | 14, 15 |
| Pheasant (Common) *Phasianus colchicus* | 625 | 0.526 | 1.468 | 0.454 | 0.771 | 0.898 |  | 9.04 |  |  | 1 |
| Red legged partridge *Alecctoris rufa* | 370 |  | 1.76 | 0.40 |  |  | 0.21 |  |  |  | 8 |
| Wild turkey *Meleagris gallopavo* | 5000 |  | 1.8 | 0.601 | 0.280 | 0.092 |  |  |  |  | 13, 16, 17 |
| Willow ptarmigan *Lagopus lagopus* | 541 | 0.978 | 2.440 | 1.601 | 0.808 | 1.881 |  |  |  |  | 1 |
| **Gaviformes** |  |  |  |  |  |  |  |  |  |  |  |
| Red-throated loon *Gavia stellata* | 1549 | 0.403 | 4.350 | 1.452 | 1.463 | 1.451 |  | 6.367 |  |  | 1 |
| **Gruiformes** |  |  |  |  |  |  |  |  |  |  |  |
| Australasian swamphen (*Porphyrio melanotus*) | 902.5 |  | 2.40 | 0.688 | 0.585 | 0.88 | 0.057 |  | 6.33 |  | 3 |
| Black crowned crane Balearica pavonina | 4448 | 0.297 | 2.363 | 0.827 | 0.584 | 0.992 |  |  |  |  | 1 |
| Sandhill crane *Antigone* canadensis | 1651 | 0.520 | 1.777 | 1.151 | 0.709 | 0.941 | 0.0406 | 4.760 |  |  | 1 |
| Takahe (*Porphyrio hochstetteri*) | 2660 |  | 2.66 | 0.71 | 0.87 | 1.25 | 0.11 |  | 5.81 |  | 3 |
| **Otidiformes** |  |  |  |  |  |  |  |  |  |  |  |
| Greater bustard *Ardeotis kori* | 7770 | 0.184 | 1.921 | 1.012 | 0.569 | 1.0967 |  | 7.368 |  |  | 1 |
| Houbara bustard (*Chlamydotis undulata*) | 1143 |  | 1.96 |  |  |  |  |  |  |  | 32 |
| Kori bustard (*Ardeotis kori*) | 7274 |  | 1.54 |  |  |  |  |  |  |  | 32 |
| Rufous-crested Bustard (*Eupodotis ruficrista*) | 509 |  | 1.85 |  |  |  |  |  |  |  | 32 |
| White-bellied Bustard (*Eupodotis senegalensis*) | 944 |  | 1.82 |  |  |  |  |  |  |  | 32 |
| **Passeriformes** |  |  |  |  |  |  |  |  |  |  |  |
| American crow Corvus brachyrhynchos | 337 | 2.760 |  | 0.950 |  | 2.958 |  |  |  |  | 1 |
| American goldfinch *Spinus tristis* | 11.4 | 4.49 | 3.03 | 1.69 | 1.56 |  |  | 3.28 | 2.16 | 1.12 | 20 |
| American robin Turdus migratorius | 71.5 | 2.52 | 2.89 | 1.36 | 1.50 |  |  |  | 1.97 | 2.03 | 1, 21 |
| Barn swallow *Hirundo rustica* | 21.25 | 4.195 |  | 1.407 |  |  |  |  |  |  | 1 |
| Barred Antshrike *Thamnophilus doliatus* | 29.9 | 3.02 | 5.13 | 1.14 | 1.61 |  |  | 8.07 | 6.20 | 1.87 | 20 |
| Blackbird *Quiscalus quiscula* | 82 | 3.561 | 3.207 | 1.413 | 1.584 | 2.126 | 0.062 | 7.78 |  |  | 1 |
| Black-striped Sparrow *Arremonops conirostris* | 39.9 | 3.32 | 3.30 | 0.88 | 0.95 |  |  | 2.74 | 0.83 | 1.91 | 20 |
| Blue-black grosbeak *Cyanoloxia cyanoides* | 26.8 | 4.19 | 2.44 | 0.95 | 0.79 |  |  | 5.01 | 3.79 | 1.22 | 20 |
| Blue-crowned manakin *Lepidothrix coronata* | 8.6 | 4.13 | 2.80 | 1.23 | 1.85 |  |  | 6.80 | 5.73 | 1.07 | 20 |
| Blue-gray tanager *Tangara episcopus* | 31.2 | 3.07 | 3.87 | 1.34 | 1.08 |  |  | 3.63 | 1.35 | 2.28 | 20 |
| Brown-headed cowbird Molothrus ater | 52.5 | 3.48 | 3.17 | 1.35 | 1.37 |  |  |  | 3.17 | 2.28 | 1, 20 |
| Buff-throated woodcreeper *Xiphorhynchus guttatus* | 43.6 | 3.26 | 2.28 | 0.94 | 1.39 |  |  | 3.98 | 3.53 | 0.45 | 20 |
| Canary *Serinus canaria* | 16.2 | 4.358 | 5.395 | 1.290 | 1.648 | 1.549 | 0.111 | 10.74 |  |  | 1 |
| Chipping sparrow *Spizella passerina* | 11 | 4.17 | 3.04 | 1.24 | 1.44 |  |  | 5.39 | 4.26 | 1.13 | 20 |
| Clay-colored thrush *Turdus grayi* | 65 | 2.27 | 2.82 | 0.98 | 1.01 |  |  | 5.14 | 2.55 | 2.59 | 20 |
| Common grackle *Quiscalus quiscula* | 108.2 | 2.48 | 3.22 | 1.14 | 1.32 |  |  | 5.53 | 3.41 | 2.12 | 20 |
| Eastern bluebird Sialia sialis | 31.5 | 4.241 |  | 1.390 |  |  |  |  |  |  | 1 |
| Eastern phoebe *Sayornis phoebe* | 17.3 | 3.83 | 2.26 | 1.144 | 1.89 |  |  | 4.74 | 3.90 | 0.84 | 1, 20 |
| Golden-collared manakin *Manacus vitellinus* | 15 | 3.33 | 3.46 | 1.16 | 1.54 |  |  | 8.25 | 6.44 | 1.81 | 20 |
| Golden-hooded tanager *Tangara larvata* | 2.45 | 2.45 | 3.57 | 1.42 | 1.50 |  |  |  | 3.50 | 1.13 | 20 |
| Grey-breasted martin *Progne chalybea* | 35.7 | 2.59 | 2.59 | 1.64 | 1.39 |  |  | 6.70 | 4.64 | 2.06 | 20 |
| Grey catbird *Dumetella carolinensis* | 33 | 4.2788 |  | 0.991 |  | 1.839 |  |  |  |  | 1 |
| House sparrow (*Passer domesticus*) | 23.4 | 4.370 | 3.743 | 1.709 | 1.499 | 1.638 | 0.148 | 11.49 |  |  | 1, 7 |
| House wren *Troglodytes aedon* | 9.4 | 5.36 | 3.72 | 1.33 | 1.94 |  |  | 5.49 | 4.21 | 1.28 | 20 |
| Mangrove swallow *Tachycineta albilinea* | 13.4 | 3.42 | 3.70 | 1.30 | 1.65 |  |  | 7.63 | 4.47 | 3.16 | 20 |
| Northern cardinal *Cardinalis cardinalis* | 30.1 | 3.49 | 2.82 | 1.18 | 1.57 |  |  | 6.09 | 4.41 | 1.68 | 20 |
| Northern mockingbird *Mimus polyglottos* | 46.6 | 2.59 | 3.63 | 1.35 | 1.67 |  |  |  | 3.79 | 2.02 | 20 |
| Palm tanager *Tangara palmarum* | 34.5 | 3.06 | 3.30 | 1.19 | 0.98 |  |  | 3.79 | 1.67 | 2.12 | 20 |
| Plain xenops *Xenops minutus* | 11 | 3.43 | 2.22 | 1.69 | 0.87 |  |  | 3.78 | 3.38 | 0.4 | 20 |
| Raven *Corvus corax* | 1338 |  |  |  | 0.181 |  |  |  |  |  | 1 |
| Red-capped manakin *Pipra mentalis* | 12.2 | 3.35 | 2.50 | 1.12 | 1.66 |  |  | 6.46 | 5.31 | 1.15 | 20 |
| Red-eyed vireo *Vireo olivaceus* | 15.1 | 3.72 | 2.96 | 1.81 | 1.94 |  |  |  | 2.54 | 1.01 | 20 |
| Red-winged blackbird (*Agelaius phoeniceus*) |  | 4.89 |  |  |  |  |  |  |  |  | 4 |
| Scarlet tanager *Piranga olivacea* | 27.3 | 3.23 | 3.03 | 1.22 | 1.67 |  |  |  | 2.80 | 1.38 | 20 |
| Social flycatcher *Myiozetetes similis* | 22.8 | 2.37 | 2.56 | 1.21 | 1.08 |  |  | 5.55 | 4.09 | 1.46 | 20 |
| Song sparrow Melospiza melodia | 20.1 | 4.79 | 3.21 | 1.119 | 1.57 |  |  | 5.36 | 3.62 | 1.74 | 1, 20 |
| Southern House wren *Troglodytes musculus* | 12.8 | 4.46 | 3.20 | 0.32 | 1.35 |  |  | 5.28 | 3.59 | 1.69 | 20 |
| Spotted antbird *Hylophylax naevioides* | 16.1 | 3.46 | 3.90 | 1.04 | 1.46 |  |  | 5.90 | 4.53 | 1.37 | 20 |
| Starling *Sturnus vulgaris* | 57.85 | 3.194 | 3.613 | 1.555 | 1.782 | 1.869 | 0.0932 | 9.422 |  |  | 1 |
| Stripe-throated wren *Contorchilus leucotis* | 20.3 | 4.25 | 3.48 | 0.99 | 1.37 |  |  |  | 4.21 | 1.37 | 20 |
| Thick-billed euphonia *Euphonia laniirostris* | 12.8 | 4.67 | 5.02 | 1.27 | 1.35 |  |  | 3.07 | 0.38 | 2.69 | 20 |
| Tree swallow *Tachycineta bicolor* | 16.3 | 2.92 | 3.41 | 1.21 | 1.94 |  |  | 6.83 | 4.62 | 2.21 | 20 |
| Tropical mockingbird *Mimus gilvus* | 59.8 | 2.54 | 2.90 | 0.81 | 1.21 |  |  | 5.67 | 3.21 | 2.46 | 20 |
| Variable seedeater *Sporophila corvina* | 10.1 | 5.38 | 3.54 | 1.28 | 0.81 |  |  | 4.73 | 3.35 | 1.38 | 20 |
| Verdin *Auriparus aviceps* | 6.92 |  | 3.42 | 1.47 | 1.12 | 1.25 |  |  |  |  | 5 |
| White-cheeked antbird *Gymnopithys leucaspis* | 30.2 | 2.38 | 3.81 | 1.28 | 1.81 |  |  | 4.05 | 3.20 | 0.85 | 20 |
| White-crowned sparrows *Zonotrichia leucophrys* | 28.4 |  | 3.496 |  |  |  | 0.128 |  |  |  | 6 |
| Yellow-green vireo *Vireo flavoviridis* | 17.1 | 3.37 | 3.25 | 1.33 | 1.55 |  |  | 4.89 | 3.02 | 1.87 | 20 |
| Yellow warbler *Dendroica petechia* | 7.2 | 5.49 | 2.98 | 1.47 | 1.87 |  |  | 3.95 | 3.01 | 0.94 | 20 |
| *Pipromorpha oliaginea* | 9.5 | 4.21 | 2.65 | 1.32 | 1.37 |  |  | 5.69 | 4.22 | 1.47 | 20 |
| **Pelecaniformes** |  |  |  |  |  |  |  |  |  |  |  |
| Brown pelican *Pelecanus occidentalis* | 3290 | 0.531 | 2.223 | 0.672 |  | 0.907 |  | 7.751 |  |  | 1 |
| Great egret *Ardea alba* | 1030 | 0.594 | 3.206 | 0.898 | 0.79 | 3.21 | 1.109 |  |  |  | 1 |
| Hamerkop *Scopus umbretta* | 317.5 | 1.238 |  | 2.27 |  | 2.570 |  |  |  |  | 1 |
| Lesser egret *Ardea intermedia* | 525 | 0.895 | 3.149 | 0.953 | 0.709 |  |  |  |  |  | 1 |
| Marabou stork *Leptoptilos crumenifer* | 7130 | 0.422 | 1.543 | 0.775 | 0.602 | 1.013 |  | 9.71 |  |  | 1 |
| **Phoenicopteriformes** |  |  |  |  |  |  |  |  |  |  |  |
| Lesser flamingo *Phoenicoparrus minor* |  |  |  |  |  |  |  |  |  |  | 1 |
| **Psittaciformes** |  |  |  |  |  |  |  |  |  |  |  |
| Orange-chinned parakeet *Brotogeris jugularis* | 57.2 | 3.76 | 2.50 | 1.58 | 0.71 |  |  | 2.27 | 1.10 | 1.17 | 20 |
| **Strigiformes** |  |  |  |  |  |  |  |  |  |  |  |
| Barn owl *Tyto alba* | 310 |  | 2.83 | 0.87 | 0.78 |  |  |  |  |  | 2 |
| Great horned owl *Bubo virginianus* | 1177 | 1.164 |  | 0.731 |  | 0.909 |  |  |  |  | 1 |
| Long-eared owl *Asio otus* | 270 |  | 2.10 | 0.98 | 0.84 |  |  |  |  |  | 2 |
| Short eared owl *Asio flammeus* | 350 |  | 3.17 | 1.22 | 0.98 |  |  |  |  |  | 2 |
| Tawny owl *Strix aluco* | 460 |  | 2.55 | 0.71 |  |  |  |  |  |  | 2 |
| **Struthioniformes** |  |  |  |  |  |  |  |  |  |  |  |
| Ostrich *Struthio camelus* | 12,300 | 0.0342 | 1.667 | 0.980 |  |  |  |  |  |  | 1 |
| **Suliformes** |  |  |  |  |  |  |  |  |  |  |  |
| Ascension frigatebird *Fregata aquila* | 1405 | 0.673 | 2.554 | 1.181 |  |  |  |  |  |  | 1 |
| Double-crested cormorants *Phalacrocorax auritus* | 1850 |  | 2.65 | 1.09 | 0.99 |  | 0.053 |  |  |  | 30 |

**References**

1. Crile and Quiring, 1940.

2. Barton and Houston, 1996.

3. Suttie and Fennessy, 1992.

4. Fisher and Bartlett, 1957.

5. Austin, 1971.

6. Oakeson, 1956.

7. Love et al., 2017.

8. Millán et al., 2001.

9. Ferreyra et al., 2015.

10. Lautenschlager et al., 1979.

11. Putaala and Hissa, 1995.

12. Musundire et al., 2018.

13. Hünigen et al., 2016.

14. Wall, and Anthony, 1995.

15. Agnvall et al., 2017.

16. Quist et al., 2000.

17. Hünigen et al., 2016.

18. Sedinger, 1986.

19. Kalisińska et al., 1999.

20. Wiersma et al., 2012.

21. Abood et al., 2014.

22. Albers et al., 2006.

23. Borin et al., 2006.

24. Gasaway and Buss, 1972.

25. Krapu, 1981.

26. White et al., 1978.

26A. Hermier et al., 2003.

27. Wilkanowska et al., 2013.

28. Donaldson et al., 2017.

29. Iwaniuk and Nelson, 2001.

30. Harr et al., 2017.

31. Kalisińska et al., 1999.

32. Bailey et al., 1997.

**Supplementary Table 8.**  Relative organ weights in mammals

| Species | B. wt.  in kg | Relative weights | | | | | | Reference |
| --- | --- | --- | --- | --- | --- | --- | --- | --- |
|  |  | Brain | Liver | Heart | Kidney | Lung | Spleen |  |
| Order **Artiodactyla** |  |  |  |  |  |  |  |  |
| Beluga whale *Delphinapterus leucas* | 372.27 | 0.394 | 1.591 | 0.568 | 0.612 | 2.617 | 0.0505 | Crile and Quiring, 1940 |
| Blue whale *Balaenoptera musculus* | 58059 | 1.827 |  |  |  |  |  | Crile and Quiring, 1940 |
| Bottlenose dolphin *Tursiops truncatus* | 166.3 | 0.885 | 2.589 | 0.565 | 0.364 | 1.837 | 0.0559 | Turner et al., 2006 |
| Chinese white dolphin *Sousa chinensis* | 189 |  | 1.93 | 0.52 | 0.67 | 3.25 |  | Plön et al., 2012 |
| Harbor porpoise *Phocoena phocoena* | 142.43 | 1.21 | 2.08 | 0.518 |  | 3.686 |  | Crile and Quiring, 1940 |
| Indo-Pacific bottlenose dolphin *Tursiops aduncus* | 230 |  | 2.03 | 0.60 | 0.8 | 3.80 |  | Plön et al., 2012 |
| Long-beaked common dolphin *Delphinus capensis* | 230 |  | 2.47 | 0.62 | 0.85 | 3.70 |  | Plön et al., 2012 |
| North Atlantic fin whale *Balaenoptera physalus* | 45045 |  | 0.926 | 0.425 | 0.176 |  |  | McAlpine, 1985 |
| Sei whale *Balaenoptera* *borealis* | 20000 |  | 1.074 | 0.377 | 0.165 |  |  | McAlpine, 1985 |
| Sperm whale *Physeter* *microcephalus* | 47045 |  | 0.838 | 0.244 | 0.155 |  |  | McAlpine, 1985 |
| African buffalo Syncerus caffer | 693.3 | 0.0944 | 1.135 | 0.505 | 0.262 | 0.984 |  | Crile and Quiring, 1940 |
| American bison *Bison bison* | 54.66 | 0.611 | 1.271 | 0.66 | 0.468 | 2.177 | 0.267 | Crile and Quiring, 1940 |
| Bohor reedbuck *Redunca redunca* | 31.7 | 0.344 | 1.65 | 0.763 | 0.322 | 1.457 |  | Crile and Quiring, 1940 |
| Caribou *Rangifer tarandus* | 987.5 | 0.361 | 1.276 | 0.959 | 0.116 | 1.842 | 0.212 | Crile and Quiring, 1940 |
| Cattle *Bos taurus* | 577.1 | 0.0821 | 1.086 | 0.374 | 0.201 | 0.702 | 0.172 | Crile and Quiring, 1940 |
| Chital *Axis axis* | 88.4 | 1.957 | 0.8473 | 0.279 | 1.952 | 0.248 |  | Crile and Quiring, 1940 |
| Collared peccary *Pecari tajacu* | 21.6 | 0.426 | 2.672 | 0.554 | 0.45 | 0.914 | 0.442 | Crile and Quiring, 1940 |
| Desert warthog *Phacochoerus aethiopicus* | 65.3 | 0.191 | 2.297 | 0.498 | 0.459 | 0.842 |  | Crile and Quiring, 1940 |
| Elk *Cervus canadensis* | 13.61 | 1.427 | 2.748 | 0.963 | 0.539 | 2.344 |  | Crile and Quiring, 1940 |
| Giraffe *Giraffa camelopardalis* | 1220 | 0.057 | 1.561 | 0.409 | 0.186 | 0.989 |  | Crile and Quiring, 1940 |
| Goat *Capra* aegagrus | 27.66 | 0.416 | 1.898 |  |  |  |  | Crile and Quiring, 1940 |
| Harnessed bushbuck *Tragelaphus scriptus* | 44.2 | 0.377 | 1.92 | 0.789 | 0.397 | 1.802 | 0.262 | Crile and Quiring, 1940 |
| Hartebeest *Alcelaphus buselaphus* | 134 | 0.205 | 1.138 | 0.653 | 0.2889 | 1.381 | 0.466 | Crile and Quiring, 1940 |
| Hippopotamus *Hippopotamus amphibius* | 1351 | 0.0533 | 1.745 | 0.336 | 0.234 | 0.839 | 0.235 | Crile and Quiring, 1940 |
| Impala *Aepyceros melampus* | 47.7 | 0.394 | 1.541 | 0.685 | 0.41 | 1.307 | 0.555 | Crile and Quiring, 1940 |
| Kirk’s dik-dik *Madoqua kirkii* | 4.57 | 0.810 | 2.039 | 0.805 | 0.468 | 0.972 |  | Crile and Quiring, 1940 |
| Mule deer *Odocoileus hemionus* | 54.5 |  |  |  | 0.415 |  |  | Crile and Quiring, 1940 |
| Pigs *Sus scrofa* | 104.9 | 0.109 | 1.599 | 0.326 | 0.227 | 0.265 | 0.131 | Crile and Quiring, 1940 |
| Red deer *Cervus elaphus* | 94.8 | 0.302 | 1.352 | 0.830 | 0.271 | 1.348 | 0.527 | Grace et al., 2008 |
| Sambar *Rusa unicolor* | 148 |  |  |  | 0.214 |  |  | Crile and Quiring, 1940 |
| Sheep *Ovis aries* | 46.1 | 0.238 | 1.836 | 0.474 | 0.274 | 0.307 |  | Crile and Quiring, 1940 |
| Sika deer *Cervus nippon* | 42 |  | 1.786 | 0.762 | 0.333 | 1.714 | 0.452 | Hanzal et al., 2018 |
| Steenbok *Raphicerus campestris* | 8.62 | 0.574 | 2.030 | 0.838 | 0.447 | 1.740 |  | Crile and Quiring, 1940 |
| Thomson’s gazelle *Eudorcas thomsonii* | 24.37 | 0.377 | 2.154 | 1.005 | 0.431 | 1.149 |  | Crile and Quiring, 1940 |
| White-tailed deer *Odocoileus virginianus* | 65.09 | 1.575 | 0.971 |  | 2.025 | 0.323 |  | Crile and Quiring, 1940 |
| Order **Carnivora** |  |  |  |  |  |  |  |  |
| Arctic fox *Vulpes lagopus* | 3.385 | 1.315 |  |  |  |  |  | Crile and Quiring, 1940 |
| Bat-eared fox *Otocyon megalotis* | 3.335 | 0.782 | 4.298 | 0.739 | 0.957 | 1.051 |  |  |
| Bearded seal *Erignathus barbatus* | 195.3 | 0.164 | 1.941 | 0.442 | 0.456 | 1.614 |  | Crile and Quiring, 1940 |
| Black-backed jackal *Canis mesomelas* | 2.85 | 1.614 | 4.298 | 0.748 | 0.80807018 | 1.055 |  | Crile and Quiring, 1940 |
| Brown fur seal *Arctocephalus pusillus* | 180 |  | 3.8 | 0.5 | 0.8 | 2.5 | 0.2 | Stewardson et al., 1999 |
| Bobcat *Lynx rufus* | 4.31 |  |  |  | 0.571 |  |  | Crile and Quiring, 1940 |
| Cape genet *Genetta tigrina* | 1.413 | 1.113 | 3.827 | 0.566 | 0.803 | 1.238 |  | Crile and Quiring, 1940 |
| Cheetah *Acinonyx jubatus* | 31.4 | 0.011 | 2.45 | 0.39 | 0.355 | 0.882 | 0.116 | Crile and Quiring, 1940 |
| Cougar *Puma concolor* | 28.8 | 0.371 | 4.359 | 0.639 | 0.471 | 1.132 |  | Crile and Quiring, 1940 |
| Coyote *Canis latrans* | 8.51 | 0.99 | 3.437 | 0.854 | 0.941 | 0.72 | 0.167 | Crile and Quiring, 1940 |
| Domestic cat | 3.304 | 0.824 | 3.955 | 0.479 | 1.002 | 0.95 | 0.294 | Crile and Quiring, 1940 |
| Dog *Canis lupus* | 19.9 | 0.488 | 4.075 | 0.938 | 0.637 | 1.487 | 0.715 | Crile and Quiring, 1940 |
| Gray fox *Urocyon cinereoargenteus* | 3.820 | 0.913 | 1.355 | 0.586 | 0.512 | 0.510 |  | Crile and Quiring, 1940 |
| Grizzly bear *Ursus arctos* | 142.9 | 0.164 |  | 0.793 | 0.383 |  | 0.213 | Crile and Quiring, 1940 |
| Jaguar *Panthera onca* | 34.5 | 0.426 | 2.594 | 0.54 | 0.477 | 1.67 | 0.177 | Crile and Quiring, 1940 |
| Kinkajou *Potos flavus* | 2.62 | 1.185 | 3.763 | 0.546 |  | 2.996 |  | Crile and Quiring, 1940 |
| Leopard *Panthera pardus* | 48 | 0.281 | 1.875 | 0.417 |  | 1.042 | 0.217 | Crile and Quiring, 1940 |
| Lion *Panthera leo* | 114.7 | 0.205 | 2.543 | 0.632 | 0.708 | 1.651 | 0.28 | Crile and Quiring, 1940 |
| Pacific harbour seal *Phoca vitulina* | 107.3 | 0.412 | 4.18 | 1.337 | 0.833 | 1.752 |  | Crile and Quiring, 1940 |
| Polar bear *Ursus maritimus* | 258.3 | 0.202 | 1.788 | 0.483 | 0.439 | 0.428 | 0.123 | Crile and Quiring, 1940 |
| Puma *Puma concur* | 26.0 | 0.497 |  |  |  |  |  | Crile and Quiring, 1940 |
| Raccoon *Procyon lotor* | 4.288 | 0.998 | 4.291 | 0.796 | 1.036 | 0.855 |  | Crile and Quiring, 1940 |
| Red fox *Vulpes vulpes* | 6.392 | 1.152 | 3.748 | 0.903 | 0.306 |  |  | Crile and Quiring, 1940 |
| Ringed seal *Pusa* *hispida* | 39.57 | 0.939 | 2.748 | 0.737 | 0.613 | 1.855 | 0.317 | Crile and Quiring, 1940 |
| Ringtail *Bassariscus astutus* | 1 | 1.71 |  |  | 1.160 |  |  | Crile and Quiring, 1940 |
| Serval *Leptailurus serval* | 7.887 | 0.792 | 1.871 | 0.431 | 0.848 |  |  | Crile and Quiring, 1940 |
| South American coati *Nasua nasua* | 3.324 | 3.273 | 3.393 | 0.555 | 1.002 | 1.328 | 0.451 | Crile and Quiring, 1940 |
| Spotted hyena *Crocuta crocuta* | 62.37 | 0.281 | 5.121 | 0.716 | 0.64133397 | 10.92 |  | Crile and Quiring, 1940 |
| Striped skunk *Mephitis mephitis* | 1.70 | 0.606 | 2.688 | 0.578 | 0.594 |  |  | Crile and Quiring, 1940 |
| Tiger *Panthera tigris* | 184.5 | 0.143 | 1.136 | 0.27 |  | 0.637 |  | Crile and Quiring, 1940 |
| Walrus *Odobenus rosmarus* | 267.39 | 0.745 | 3.797 | 0.923 | 0.99 | 2.136 | 0.433 | Crile and Quiring, 1940 |
| Weasel *Mustela arctica* | 0.145 | 3.1 | 5.129 | 1.642 | 1.28 | 2.625 | 0.421 | Crile and Quiring, 1940 |
| Western hog-nosed skunk *Conepatus leuconotus* | 1.905 | 0.827 |  |  | 0.394 |  |  | Crile and Quiring, 1940 |
| Wolf *Lupus lupus* | 26.3 | 0.516 | 2.925 | 1.068 | 2.152 | 0.148 |  | Crile and Quiring, 1940 |
| White-tailed *mongoose Ichneumia* albicauda | 4.4 | 0.643 | 1.386 | 0.655 | 0.795 | 1.318 |  | Crile and Quiring, 1940 |
| Oder **Chiroptera** |  |  |  |  |  |  |  |  |
| Vampire bat *Desmodus rotundus* | 0.028 | 3.343 |  |  |  |  |  | Crile and Quiring, 1940 |
| Order **Didelphimorphia** |  |  |  |  |  |  |  |  |
| Common opossum *Didelphis marsupialis* | 1.147 | 0.418 | 5.841 | 0.436 | 0.654 | 0.828 |  | Crile and Quiring, 1940 |
| Bare-tailed wooly opossum Caluromys philander | 0.222 |  | 4.054 | 1.351 | 1.802 | 1.35 | 0.676 | Crile and Quiring, 1940 |
| Order **Eulipotyphl** |  |  |  |  |  |  |  |  |
| Eastern mole *Scalopus aquaticus* | 0.0396 | 2.929 | 3.909 | 0.687 | 1.588 | 1.861 |  | Crile and Quiring, 1940 |
| Northern short-tailed shrew *Blarina brevicauda* | 0.0175 | 1.992 | 5.633 | 1.04 | 1.264 | 2.217 |  | Crile and Quiring, 1940 |
| Order **Hyracoidea** |  |  |  |  |  |  |  |  |
| Yellow-spotted rock hyrax *Heterohyrax brucei* | 0.75 | 1.636 | 4.204 | 0.484 | 0.86 | 0.738 |  | Crile and Quiring, 1940 |
| Order **Lagomorpha** |  |  |  |  |  |  |  |  |
| Arctic hare *Lepus arcticus* | 2.270 | 0.461 | 2.974 | 1.295 | 1.054 | 1.986 |  | Crile and Quiring, 1940 |
| Black-tailed jack rabbit *Lepus californicus* | 2.4 | 0.676 |  |  | 0.485 |  |  | Crile and Quiring, 1940 |
| Cape hare *Lepus capensis* | 2.93 | 0.349 | 1.772 | 1.024 | 0.416 |  |  | Crile and Quiring, 1940 |
| Wild rabbit *sylvilagus gabbi* | 0.439 | 1.025 | 4.442 | 0.456 | 0.767 |  |  | Crile and Quiring, 1940 |
| Order **Perissodactyla** |  |  |  |  |  |  |  |  |
| Baird’s tapir *Tapirus bairdii* | 14.26 | 0.596 | 3.387 | 0.849 | 0.849 | 1.171 | 2.230 | Crile and Quiring, 1940 |
| Black rhinoceros *Diceros bicornis* | 763 | 0.0858 | 1.875 | 0.629 | 0.393 | 0.963 |  | Crile and Quiring, 1940 |
| Donkey *Equus africanus* | 234.4 | 0.252 | 1.513 | 0.688 | 0.363 | 1.172 | 0.258 | Crile and Quiring, 1940 |
| Horse *Equus ferus* | 439.6 | 0.152 | 1.248 | 0.859 | 0.329 | 1.174 | 0.712 | Crile and Quiring, 1940 |
| Plains zebra *Equus quagga* | 289.9 | 0.200 | 1.687 | 0.707 | 0.343 | 0.867 | 0.406 | Crile and Quiring, 1940 |
| Order **Pilosa** |  |  |  |  |  |  |  |  |
| Brown throated three toed sloth *Bradypus variegatus* | 3.263 | 0.493 | 2.054 | 0.240 | 0.525 | 0.958 | 0.256 | Crile and Quiring, 1940 |
| Hoffman’s two-toed sloth *Choloepus hoffmanni* | 5.048 | 0.486 |  |  |  |  |  | Crile and Quiring, 1940 |
| Nine-banded armadillo *Dasypus novemcinctus* | 3.063 | 0.353 | 0.353 | 3.117 | 0.331 | 0.479 | 0.706 | Crile and Quiring, 1940 |
| Southern tamandua *Tamandua tetradactyla* | 2.5505 | 1.155 | 1.852 | 0.315 | 0.728 | 0.933 | 0.148 | Crile and Quiring, 1940 |
| Silky anteater *Cyclopes didactylus* | 0.086 | 4.799 |  |  |  |  |  | Crile and Quiring, 1940 |
| Order **Primates** |  |  |  |  |  |  |  |  |
| Yellow baboon *Papio cynocephalus* | 13.7 | 1.335 | 1.881 | 0.410 | 0.364 | 0.897 |  | Crile and Quiring, 1940 |
| Blue monkey *Cercopithecus mitis* | 2.89 | 2.569 | 2.161 | 0.525 | 0.465 | 0.743 |  | Crile and Quiring, 1940 |
| Mantled howler *Alouatta palliata* | 5.952 | 0.917 | 3.519 | 0.292 | 0.633 | 0.767 |  | Crile and Quiring, 1940 |
| Brown woolly monkey *Lagothrix lagothricha* | 5.26 | 1.639 |  | 0.913 |  | 1.207 |  | Crile and Quiring, 1940 |
| Senegal bushbaby *Galago senegalensis* | 0.2 | 2.5 | 3.015 | 0.69 | 0.77 | 0.72 |  | Crile and Quiring, 1940 |
| Ring-tailed lemur *Lemur catta* | 1.725 | 1.264 |  | 0.475 |  | 1.432 |  | Crile and Quiring, 1940 |
| Rhesus macaque *Macaca mulatta* | 3.459 | 2.676 | 2.096 | 0.361 |  | 1.889 |  | Crile and Quiring, 1940 |
| Geoffroy's spider monkey *Ateles geoffroyi* | 8.110 | 1.353 | 2.792 | 0.426 | 0.409 | 0.673 |  | Crile and Quiring, 1940 |
| Geoffroy's tamarin *Saguinus geoffroyi* | 5.831 | 1.722 | 3.204 | 0.493 | 0.535 | 1.069 | 0.208 | Crile and Quiring, 1940 |
| Sykes' monkey *Cercopithecus albogularis* | 4.937 | 1.229 | 2.423 | 0.607 | 0.217 | 0.433 |  | Crile and Quiring, 1940 |
| Vervet monkey *Chlorocebus pygerythrus* | 3.955 | 1.540 | 2.180 | 0.810 | 0.3808 | 0.632 |  | Crile and Quiring, 1940 |
| White-headed capuchin *Cebus capucinus* | 2.9095 | 2.481 |  | 0.600 | 0.461 | 1.099 | 0.364 | Crile and Quiring, 1940 |
| Central American squirrel monkey *Saimiri oerstedii* | 0.757 | 3.034 | 4.217 | 0.606 | 0.657 | 1.186 | 0.148 | Crile and Quiring, 1940 |
| Chimpanzee *Pan troglodytes* | 50.34 | 0.757 | 2.134 | 0.441 | 0.370 | 1.058 |  | Crile and Quiring, 1940 |
| Human *Homo sapiens* | 60.8 | 2.302 | 2.511 | 0.551 | 0.404 | 1.662 | 0.154 | Crile and Quiring, 1940 |
| Order **Proboscidea** |  |  |  |  |  |  |  |  |
| African bush elephant *Loxodonta africana* | 6654 | 0.086 | 1.618 | 0.392 | 0.273 | 2.086 |  | Crile and Quiring, 1940 |
| African forest elephant *Loxodonta cyclotis* | 4309 |  |  |  | 0.12 |  |  | Crile and Quiring, 1940 |
| Order **Sirenia** |  |  |  |  |  |  |  |  |
| Dugong (*Dugong dugon*) | 99.5 | 0.245 | 1.562 | 0.245 | 0.571 | 1.508 | 0.0212 | Kamiya et al., 1979 |
| Ord**er Rodentia** |  |  |  |  |  |  |  |  |
| Abert's squirrel *Sciurus aberti* | 0.636 | 9.827 |  |  | 0.491 |  |  | Crile and Quiring, 1940 |
| American red squirrel Tamiasciurus *hudsonicus* | 0.196 | 2.315 | 2.655 | 0.786 | 0.706 |  |  | Crile and Quiring, 1940 |
| Arctic ground squirrel *Urocitellus parryii* | 0.918 | 0.62 | 4.405 | 0.642 | 0.779 |  |  | Crile and Quiring, 1940 |
| Brown rat *Rattus norvegicus* | 0.278 | 0.896 | 5.575 | 0.508 | 1.814 |  |  | Crile and Quiring, 1940 |
| Camas pocket gopher *Thomomys bulbivorus* | 0.368 | 0.361 |  |  | 0.312 |  |  | Crile and Quiring, 1940 |
| Central American agouti *Dasyprocta punctata* | 3.172 | 0.578 | 2.68 | 0.553 | 0.485 | 0.504 | 0.19 | Crile and Quiring, 1940 |
| Desert cottontail *Sylvilagus audubonii* | 0.950 | 0.676 |  |  | 0.485 |  |  | Crile and Quiring, 1940 |
| Dormouse *Graphiurus saturatus* | 0.020 | 3.137 | 2.778 | 0.581 | 0.687 | 1.228 | 0.75 | Crile and Quiring, 1940 |
| Eastern chipmunk *Tamias striatus* | 0.075 | 2.96 | 7.4 | 0.796 | 1.008 | 0.959 | 0.296 | Crile and Quiring, 1940 |
| Guatemalan deer mouse *Peromyscus guatemalensis* | 0.0169 | 2.667 | 6 | 0.722 | 1.171 | 1.707 |  | Crile and Quiring, 1940 |
| Guinea pig *Cavia porcellus* | 0.336 | 1.181 | 5.195 | 0.462 | 1.045 | 1.244 | 0.151 | Crile and Quiring, 1940 |
| Gunnison’s prairie dog *Cynomys gunnisoni* | 0.816 | 0.686 |  |  | 0.324 |  |  | Crile and Quiring, 1940 |
| Lesser capybara *Hydrochoerus isthmius* | 27.67 | 0.189 | 2.085 | 0.304 | 0.252 | 0.82 |  | Crile and Quiring, 1940 |
| Lowland paca *Cuniculus paca* | 4.093 | 0.602 | 5.156 | 0.444 | 0.625 | 0.648 | 0.333 | Crile and Quiring, 1940 |
| Meadow jumping mouse *Zapus hudsonius* | 0.0172 | 3.52 | 5.355 | 0.842 | 1.44 | 1.437 |  | Crile and Quiring, 1940 |
| Meadow vole *Microtus pennsylvanicus* | 0.0289 | 2.503 | 5.066 | 0.683 | 1.404 | 1.555 |  | Crile and Quiring, 1940 |
| Muskrat *Ondatra zibethicus* | 0.9 | 0.0592 | 2.439 | 0.359 | 0.828 | 0.483 |  | Crile and Quiring, 1940 |
| North American beaver *Castor canadensis* | 5.005 | 0.558 | 2.935 | 0.43 | 1.051 | 1.045 | 0.081 | Crile and Quiring, 1940 |
| North American brown lemming *Lemmus trimucronatus* | 0.04 | 3.162 | 7.599 | 1.303 | 1.313 | 2.027 |  | Crile and Quiring, 1940 |
| Northern collared lemming *Dicrostonyx groenlandicus* | 0.0536 | 1.627 | 5.287 | 0.644 | 1.504 | 1.002 |  | Crile and Quiring, 1940 |
| North American porcupine *Erethizon dorsatum* | 2.978 | 0.813 | 3.584 | 0.563 | 1.039 | 0.931 |  | Crile and Quiring, 1940 |
| Red-tailed squirrel *Notosciurus granatensis* | 0.287 |  | 3.833 | 0.697 | 1.045 |  |  | Crile and Quiring, 1940 |
| Southern multimammate mouse *Mastomys coucha* | 0.0218 | 3.027 | 4.33 | 0.56 | 1.216 | 1.1055 |  | Crile and Quiring, 1940 |
| Thirteen-lined ground squirrel *Ictidomys tridecemlineatus* | 0.190 | 1.053 |  |  | 0.574 |  |  | Crile and Quiring, 1940 |
| Andean mountain cavy *Microcavia niata* | 0.181 |  |  |  | 0.434 |  |  | Al-kahtani et al., 2004 |
| Spotted agouti *Agouti paca* | 3.627 |  |  |  | 0.860 |  |  | Al-kahtani et al., 2004 |
| Agouti *Dasprocta leporina* | 2.600 |  |  |  | 0.626 |  |  | Al-kahtani et al., 2004 |
| Capybara *Hydrochaeris hydrochaeris* | 27.670 |  |  |  | 0.592 |  |  | Al-kahtani et al., 2004 |
| Ashy chinchilla rat *Abrocoma cinerea* | 0.085 |  |  |  | 0.252 |  |  | Al-kahtani et al., 2004 |
| Bennett’s chinchilla rat *Abrocoma bennetti* | 0.197 |  |  |  | 1.647 |  |  | Al-kahtani et al., 2004 |
| Long-tailed chinchilla *Chinchilla lanigera* | 0.312 |  |  |  | 0.569 |  |  | Al-kahtani et al., 2004 |
| Mountain degu *Octodontomys gliroides* | 0.187 |  |  |  | 0.647 |  |  | Al-kahtani et al., 2004 |
| Common degu *Octodon degus* | 0.160 |  |  |  | 0.920 |  |  | Al-kahtani et al., 2004 |
| Bridges's degu *Octodon bridgesi* | 0.163 |  |  |  | 0.875 |  |  | Al-kahtani et al., 2004 |
| Moon-toothed degu *Octodon lunatus* | 0.171 |  |  |  | 0.564 |  |  | Al-kahtani et al., 2004 |
| Chilean rock rat *Aconaemys fuscus* | 0.128 |  |  |  | 0.784 |  |  | Al-kahtani et al., 2004 |
| Sage's rock rat *Aconaemys sagei* | 0.106 |  |  |  | 0.875 |  |  | Al-kahtani et al., 2004 |
| Coruro *Spalacopus cyanus* | 0.099 |  |  |  | 0.642 |  |  | Al-kahtani et al., 2004 |
| Val's gundi *Ctenodactylus vali* | 0.180 |  |  |  | 0.505 |  |  | Al-kahtani et al., 2004 |
| Eastern chipmunk *Tamias striatus* | 0.075 |  |  |  | 0.738 |  |  | Al-kahtani et al., 2004 |
| Cape ground squirrel *Xerus inauris* | 0.400 |  |  |  | 0.550 |  |  | Al-kahtani et al., 2004 |
| Five-striped squirrel *Funambulus pennanti* | 0.0929 |  |  |  | 0.521 |  |  | Al-kahtani et al., 2004 |
| Arctic ground squirrel *Urocitellus*  *parryii* | 0.958 |  |  |  | 0.743 |  |  | Al-kahtani et al., 2004 |
| California ground squirrel *Otospermophilus beecheyi* | 0.468 |  |  |  | 0.504 |  |  | Al-kahtani et al., 2004 |
| Black-tailed prairie dog *Cynomys ludovicianus* | 0.972 |  |  |  | 0.284 |  |  | Al-kahtani et al., 2004 |
| White-tailed prairie dog *Cynomys leucurus* | 1.110 |  |  |  | 0.335 |  |  | Al-kahtani et al., 2004 |
| Red squirrel *Tamiasciurus hudsonicus* | 0.1425 |  |  |  | 0.969 |  |  | Al-kahtani et al., 2004 |
| Spinghare *Pedetes capensis* | 0.318 |  |  |  | 3.575 |  |  | Al-kahtani et al., 2004 |
| Merriam’s kangaroo rat *Dipodomys merriami* | 0.0367 |  |  |  | 1.235 |  |  | Al-kahtani et al., 2004 |
| Chisel-toothed kangaroo rat *Dipodomys microps* | 0.062 |  |  |  | 1.210 |  |  | Al-kahtani et al., 2004 |
| Desert pocket mouse *Chaetodipus penicillatus* | 0.0159 |  |  |  | 1.186 |  |  | Al-kahtani et al., 2004 |
| Bailey’s pocket mouse *Chaetodipus baileyi* | 0.0257 |  |  |  | 0.915 |  |  | Al-kahtani et al., 2004 |
| Arizona pocket mouse *Perognathus amplus* | 0.0058 |  |  |  | 2.307 |  |  | Al-kahtani et al., 2004 |
| Meadow jumping mouse *Zapus hudsonius* | 0.0193 |  |  |  | 1.130 |  |  | Al-kahtani et al., 2004 |
| Jerboa *Jaculus jaculus* | 0.042 |  |  |  | 0.976 |  |  | Al-kahtani et al., 2004 |
| Andean swamp rat *Neotomys ebriosus* | 0.062 |  |  |  | 0.323 |  |  | Al-kahtani et al., 2004 |
| Long-tailed colilargo *Oligoryzomys longicaudatus* | 0.029 |  |  |  | 0.690 |  |  | Al-kahtani et al., 2004 |
| *Abrothrix xanthorhnus* | 0.022 |  |  |  | 1.364 |  |  | Al-kahtani et al., 2004 |
| Long-haired grass mouse *Abrothrix longipilis* | 0.025 |  |  |  | 1.840 |  |  | Al-kahtani et al., 2004 |
| Olive grass mouse *Abrothrix olivaceus* | 0.018 |  |  |  | 1.222 |  |  | Al-kahtani et al., 2004 |
| Andean Altiplano mouse *Abrothrix andinus* | 0.0195 |  |  |  | 1.056 |  |  | Al-kahtani et al., 2004 |
| Andean long-clawed mouse *Chelemys macronyx* | 0.049 |  |  |  | 1.184 |  |  | Al-kahtani et al., 2004 |
| Rufous-bellied bolo mouse *Bolomys lactens* | 0.036 |  |  |  | 0.778 |  |  | Al-kahtani et al., 2004 |
| Variable grass mouse *Akodon varius* | 0.033 |  |  |  | 0.545 |  |  | Al-kahtani et al., 2004 |
| *Akodon berlepschi* | 0.018 |  |  |  | 1.778 |  |  | Al-kahtani et al., 2004 |
| White-bellied grass mouse *Akodon albiventer* | 0.0225 |  |  |  | 1.468 |  |  | Al-kahtani et al., 2004 |
| Large vesper mouse *Calomys callosus* | 0.016 |  |  |  | 0.016 |  |  | Al-kahtani et al., 2004 |
| Andean vesper mouse *Calomys lepidus* | 0.014 |  |  |  | 0.014 |  |  | Al-kahtani et al., 2004 |
| Drylands vesper mouse *Calomys musculinus* | 0.015 |  |  |  | 0.015 |  |  | Al-kahtani et al., 2004 |
| Andean gerbil mouse *Eligmodontia puerulus* | 0.018 |  |  |  | 0.018 |  |  | Al-kahtani et al., 2004 |
| *Eligmodontia typus* | 0.016 |  |  |  | 0.016 |  |  | Al-kahtani et al., 2004 |
| *Eligmodontia hirtipes* | 0.017 |  |  |  | 1.059 |  |  | Al-kahtani et al., 2004 |
| *Eligmodontia marica* | 0.017 |  |  |  | 1.092 |  |  | Al-kahtani et al., 2004 |
| Roig's pericote *Andalgalomys roigi* | 0.029 |  |  |  | 1.075 |  |  | Al-kahtani et al., 2004 |
| Gray leaf-eared mouse *Graomys griseoflavus* | 0.056 |  |  |  | 1.084 |  |  | Al-kahtani et al., 2004 |
| Pale leaf-eared mouse *Graomys domorum* | 0.082 |  |  |  | 1.080 |  |  | Al-kahtani et al., 2004 |
| Andean mouse *Andinomys edax* | 0.065 |  |  |  | 1.082 |  |  | Al-kahtani et al., 2004 |
| Pikumche pericote *Loxodontomys pikumche* | 0.043 |  |  |  | 1.233 |  |  | Al-kahtani et al., 2004 |
| Andean big-eared mouse *Auliscomys sublimis* | 0.036 |  |  |  | 0.778 |  |  | Al-kahtani et al., 2004 |
| Bolivian big-eared mouse *Auliscomys boliviensis* | 0.033 |  |  |  | 1.515 |  |  | Al-kahtani et al., 2004 |
| Bunchgrass leaf-eared mouse *Phyllotis osilae* | 0.053 |  |  |  | 0.415 |  |  | Al-kahtani et al., 2004 |
| Osgood's leaf-eared mouse *Phyllotis osgoodi* | 0.064 |  |  |  | 0.594 |  |  | Al-kahtani et al., 2004 |
| yellow-rumped leaf-eared mouse *Phyllotis xanthopygus* | 0.058 |  |  |  | 1.264 |  |  | Al-kahtani et al., 2004 |
| *Phyllotis chilensis* | 0.027 |  |  |  | 0.963 |  |  | Al-kahtani et al., 2004 |
| *Phyllotis rupestris* | 0.036 |  |  |  | 0.778 |  |  | Al-kahtani et al., 2004 |
| *Phyllotis magister* | 0.113 |  |  |  | 0.814 |  |  | Al-kahtani et al., 2004 |
| *Phyllotis darwini* | 0.060 |  |  |  | 0.957 |  |  | Al-kahtani et al., 2004 |
| Southern grasshopper mouse *Onychomys torridus* | 0.219 |  |  |  | 1.511 |  |  | Al-kahtani et al., 2004 |
| White-footed mouse *Peromyscus leucopus* | 0.0283 |  |  |  | 0.680 |  |  | Al-kahtani et al., 2004 |
| Cactus mouse *Peromyscus eremicus* | 0.0228 |  |  |  | 1.952 |  |  | Al-kahtani et al., 2004 |
| Field vole *Microtus agrestis* | 0.030 |  |  |  | 1.6 |  |  | Al-kahtani et al., 2004 |
| Meadow vole *Microtus pennsylvanicus* | 0.02655 |  |  |  | 1.171 |  |  | Al-kahtani et al., 2004 |
| Golden hamster *Mesocricetus auratus* | 0.0593 |  |  |  | 1.029 |  |  | Al-kahtani et al., 2004 |
| Common hamster *Cricetus cricetus* | 0.108 |  |  |  | 0.922 |  |  | Al-kahtani et al., 2004 |
| Bushy- tailed jiird *Skeetamys calurus* | 0.0577 |  |  |  | 0.790 |  |  | Al-kahtani et al., 2004 |
| Shaw's jird *Meriones shawi* | 0.1707 |  |  |  | 0.367 |  |  | Al-kahtani et al., 2004 |
| Jird *Meriones tristrami* | 0.0551 |  |  |  | 0.686 |  |  | Al-kahtani et al., 2004 |
| Sundevall's Jird *Meriones crassus* | 0.0671 |  |  |  | 0.686 |  |  | Al-kahtani et al., 2004 |
| Wagner's gerbil *Gerbillus dasyurus* | 0.028 |  |  |  | 0.629 |  |  | Al-kahtani et al., 2004 |
| Lesser Egyptian gerbil *Gerbillus gerbillus* | 0.0327 |  |  |  | 0.803 |  |  | Al-kahtani et al., 2004 |
| Australian hopping mouse *Notomys alexis* | 0.029 |  |  |  | 1.159 |  |  | Al-kahtani et al., 2004 |
| Eastern broad-toothed field mouse *Apodemus mystacinus* | 0.0364 |  |  |  | 0.687 |  |  | Al-kahtani et al., 2004 |
| House mouse *Mus musculus* | 0.012 |  |  |  | 0.667 |  |  | Al-kahtani et al., 2004 |
| African grass rat *Arvicanthis niloticus* | 0.122 |  |  |  | 1.067 |  |  | Al-kahtani et al., 2004 |
| Angoni vlei rat *Otomys angoniensis* | 0.1158 |  |  |  | 1.045 |  |  | Al-kahtani et al., 2004 |
| Sloggett's vlei rat *Otomys sloggetti* | 0.1023 |  |  |  | 0.938 |  |  | Al-kahtani et al., 2004 |
| Southern African vlei rat *Otomys irroratus* | 0.1723 |  |  |  | 1.021 |  |  | Al-kahtani et al., 2004 |
| Bush vlei rat *Otomys unisulcatus* | 0.1176 |  |  |  | 1.599 |  |  | Al-kahtani et al., 2004 |
| Littledale's whistling rat *Parotomys littledalei* | 0.107 |  |  |  | 1.310 |  |  | Al-kahtani et al., 2004 |
| Brants's whistling rat *Parotomys brantsii* | 0.112 |  |  |  | 1.454 |  |  | Al-kahtani et al., 2004 |
| Cairo spiny mouse *Acomys caririnus* | 0.0347 |  |  |  | 0.518 |  |  | Al-kahtani et al., 2004 |
| Golden spiny mouse *Acomys russatus* | 0.0528 |  |  |  | 0.434 |  |  | Al-kahtani et al., 2004 |

**Supplementary Table 9.**  Liver DNA and cellularity

| Species | DNA mg  g^-1^ | Reference for liver DNA concentration | Genome size diploid pg | Reference to genome size | Cells x 10^9^ per g |
| --- | --- | --- | --- | --- | --- |
| **Mammals** |  |  |  |  |  |
| Cat | 2.05 | Center et al., 2002 | 5.4 | Kapusta et al., 2017 | 0.380 |
| Cattle | 3.55 | Patterson et al., 1968; Munro and Gray, 1969 | 6 | Kapusta et al., 2017 | 0.592 |
| Dog | 2.14 | Munro and Gray, 1969; Center et al., 2002 | 5 | Kapusta et al., 2017 | 0.428 |
| Guinea pig | 0.375 | Bhatavdekar et al., 1977 | 6.8 | Kapusta et al., 2017 | 0.055 |
| Horse | 0.40 | Munro and Gray, 1969 | 5.2 | Kapusta et al., 2017 | 0.077 |
| Mouse | 1.69 | Munro and Gray, 1969; Bhatavdekar et al., 1977 | 6 | Kapusta et al., 2017 | 0.282 |
| Rabbit | 1.40 | Munro and Gray, 1969 | 7.0 | Kapusta et al., 2017 | 0.200 |
| Rat | 3.00 | Munro and Gray, 1969; | 6.3 | Kapusta et al., 2017 | 0.476 |
| Sheep | 3.62 | Johns and Bergen, 1976; Burrin et al., 1992 | 5.2 | Kapusta et al., 2017 | 0.693 |
|  |  |  |  |  |  |
| **Birds** |  |  |  |  |  |
| Chicken | 4.3 | Neill et al., 1977 | 2.15 | Mendonça et al., 2010; 2016 | 2.10 |
| Domestic duck | 6.13 | Holmes and Stewart, 1968 | 2.42 | Kapusta et al., 2017 | 2.53 |
| Domestic Goose | 0.842 | Bulla et al., 2010 | 1.01 | Andraszek et al., 2009 | 0.83 |
| Japanese quail | 1.765 | Bulla et al., 1974; Malik et al., 2012 | 2.15 | Based on chicken | 0.86 |

**Supplementary Table 10**. Comparison of body weights with combined weight of the stomach and small intestine

| Species | B. wt.  in kg | SI + stomach g | Reference |
| --- | --- | --- | --- |
| Order **Artiodactyla** |  |  |  |
| Beluga whale *Delphinapterus leucas* | 372.27 | 21371 | Crile and Quiring, 1940 |
| Harbor porpoise *Phocoena phocoena* | 142.43 | 13255 | Crile and Quiring, 1940 |
| African buffalo *Syncerus caffer* | 6665 | 14800 | Crile and Quiring, 1940 |
| Bushbuck (*Tragelaphus scriptus*) | 53.07 | 6700 | Crile and Quiring, 1940 |
| Caribou *Rangifer tarandus* | 128.47 | 25400 | Crile and Quiring, 1940 |
| Cattle *Bos taurus* | 503 | 23751 | Crile and Quiring, 1940 |
| Giraffe *Giraffa camelopardalis* | 1220 | 130000 | Crile and Quiring, 1940 |
| Goat *Capra aegagrus* | 27.66 | 1466 | Crile and Quiring, 1940 |
| Hartebeest *Alcelaphus buselaphus* | 134 | 14540 | Crile and Quiring, 1940 |
| Hippopotamus *Hippopotamus amphibius* | 1351 | 374000 | Crile and Quiring, 1940 |
| Impala *Aepyceros melampus* | 37.86 | 5800 | Crile and Quiring, 1940 |
| Pigs *Sus scrofa* | 104.9 | 1824 | Crile and Quiring, 1940 |
| Steenbok *Raphicerus campestris* | 8.62 | 550 | Crile and Quiring, 1940 |
| Thomson’s gazelle *Eudorcas thomsonii* | 2.43 | 380 | Crile and Quiring, 1940 |
| Warthog (*Phacochoerus aethiopicus)* | 65.32 | 950 | Crile and Quiring, 1940 |
| Order **Carnivora** |  |  |  |
| Bat-eared fox *Otocyon megalotis* | 3.335 | 660 | Crile and Quiring, 1940 |
| Domestic cat | 3.304 | 194.5 | Crile and Quiring, 1940 |
| Dog *Canis lupus* | 19.9 | 1362 | Crile and Quiring, 1940 |
| Genet (*Genettatigrina* ) | 1525 | 97 | Crile and Quiring, 1940 |
| Gray fox *Urocyon cinereoargenteus* | 3.820 | 425 | Crile and Quiring, 1940 |
| Jackal (*Thos mesometas*) | 2.85 | 308 | Crile and Quiring, 1940 |
| Polar bear *Ursus maritimus* | 258.3 | 8186 | Crile and Quiring, 1940 |
| Raccoon (*Procyon lotor*) | 2.266 | 225 | Crile and Quiring, 1940 |
| Ringer seal (*Phoca hispida*) | 36.46 | 2992 | Crile and Quiring, 1940 |
| Serval *Leptailurus serval* | 7.887 | 375 | Crile and Quiring, 1940 |
| Spotted hyena *Crocuta crocuta* | 62.37 | 6804 | Crile and Quiring, 1940 |
| Walrus *Odobenus rosmarus* | 667 | 29484 | Crile and Quiring, 1940 |
| Wild cat (*Felis ocreata* ) | 2.7 | 78 | Crile and Quiring, 1940 |
| White-tailed *mongoose Ichneumia* albicauda | 4.4 | 275 | Crile and Quiring, 1940 |
| Order **Hyracoidea** |  |  |  |
| Yellow-spotted rock hyrax *Heterohyrax brucei* | 0.75 | 255 | Crile and Quiring, 1940 |
| Order **Lagomorpha** |  |  |  |
| Arctic hare *Lepus arcticus* | 2.93 | 295 | Crile and Quiring, 1940 |
| Order **Primates** |  |  |  |
| Senegal bushbaby *Galago senegalensis* | 0.2 | 17.6 | Crile and Quiring, 1940 |
| Gray monkey *Cercopithecusmitis* | 2.06 | 300 | Crile and Quiring, 1940 |
| Vervet monkey *Chlorocebus pygerythrus* | 1.255 | 250 | Crile and Quiring, 1940 |
| Chimpanzee *Pan troglodytes* | 59.69 | 6200 | Crile and Quiring, 1940 |
| Order **Proboscidea** |  |  |  |
| African bush elephant *Loxodonta africana* | 6654 | 924000 | Crile and Quiring, 1940 |
| Ord**er Rodentia** |  |  |  |
| African Mouse (*Mastomys coucha)* | 0.0218 | 2.63 | Crile and Quiring, 1940 |
| Brown rat *Rattus norvegicus* | 0.278 | 7.02 | Crile and Quiring, 1940 |
| Chipmunk *Tamias striatus* | 0.075 | 4.65 | Crile and Quiring, 1940 |
| Dormouse *Graphiurus saturatus* | 0.020 | 0.75 | Crile and Quiring, 1940 |
| Muskrat *Ondatra zibethicus* | 0.9 | 176 | Crile and Quiring, 1940 |
| Order **Sirenia** |  |  |  |
| Dugong (*Dugong dugon*) | 139.2 | 4783 | Kamiya et al., 1979 |

**Supplementary Table11.** Plasma/serum concentrations of immunoglobulin in mammals and birds

| Species | Concentration mg mL^-1^ | | | Reference |
| --- | --- | --- | --- | --- |
|  | IgG/IgY | IgA | IgM |  |
| **Mammals** |  |  |  |  |
| Forest musk deer (*Moschus berezovskii*) | 0.154 | 0.015 | 0.0267 | Liu et al., 2019 |
| Human (*Homo sapiens*) | 10.9 | 2.33 | 1.27 | Lobo et al., 2004; Gonzalez-Quintela et al., 2008 |
| Crab-eating macaque (*Macaca fascicularis*) | 15.4 | 2.33 | 1.33 | Cole and Bowen, 1976 |
| Rhesus macaque (*Macaca mulatta*) | 10.89 | 3.3 | 0.68 | Monte-Wicher et al., 1970; Challcombe et al., 1979 |
| Horse (*Equus ferus*) | 23.32 | 3.05 | 1.19 | Kohn et al., 1989; Perkins et al., 2003 |
| Plains zebras (*Equus quagga)* | 0.306 |  |  | Seeber et al., 2020 |
| Mountain zebras (*Equus zebra*) | 0.312 |  |  | Seeber et al., 2020 |
| Cattle (*Bos taurus*) | 2.34 | 0.121 | 2.454 | Zhao et al., 2010. |
| European bison (*Bison bonasus*) | 32.5 | 0.19 | 2.05 | Pomorska-Mól et al., 2020 |
| Pig (*Sus scrofa*) | 39.56 | 1.58 | 6.12 | Markowska-Daniel et al., 2010 |
| **Mean + (n = species) SEM** | **13.6 + (10) 4.48** | **1.61 + (6) 0.48** | **1.89 + (6) 0.66** |  |
| **Birds** |  |  |  |  |
| Chicken (*Gallus gallus*) | 10.7 | 0.332 | 1.32 | Hamal et al., 2006; Çetin et al., 2010; Agrawal et al., 2016; Murai et al., 2020 |
| Duck (*Anas platyrhynchos*) | 2.0 |  | 2.5 | Ng and Higgins, 1986 |
| Turkey (*Meleagris gallopavo*) | 21.1 |  | 2.42 | Kowalczyk et al., 2019 |
| **Mean + (n = species) SEM** | **8.69 + (3) 6.21** | **0.332** | **2.08 + (3) 0.38** |  |

**Supplementary Table12.** Small intestine length and body weight in mammals

| Species | Body weight | Small intestine length | | Reference |
| --- | --- | --- | --- | --- |
|  | kg | m | cm kg^-1^ |  |
| Bandicoot (*Isoodon macrourus*) | 1.365 | 1.05 | 76.9 | McClelland et al., 1999 |
| Horse (*Equus caballus*) | 685 | 22.4 | 3.27 | Kararli, 1995 |
| Cattle (*Bos taurus*) | 488 | 46 | 9.43 | Kararli, 1995 |
| Sheep (*Ovis aries*) | 115 | 23.6 | 20.5 | Gabella 1987; Kararli, 1995 |
| Pig (*Sus scrofa*) | 82 | 18.29 | 22.3 | Kararli, 1995 |
| Dog (*Canis Lupus*) | 10 | 4.14 | 41.4 | Kararli, 1995 |
| Cat (*Felis cattus*) | 4.5 | 1.72 | 38.2 | Kararli, 1995 |
| Rabbit (*Oryctolagus cuniculus*) | 4.7 | 3.56 | 75.7 | Kararli, 1995 |
| House mouse (*Mus musculus*) | 0.024 | 0.465 | 1937.5 | Gabella, 1987; Kararli, 1995 |
| Brown rat (*Rattus norvegicus*) | 0.337 | 1.2525 | 371.7 | Kararli, 1995 |
| Human (*Homo sapiens*) | 62 | 6 | 9.68 | Kararli, 1995 |
| Guinea pig (*Cavia porcellus*) | 0.950 | 1.45 | 152.6 | Gabella, 1987 |
| Common shrew (*Sorex araneus*) | 0.0088 | 0.1004 | 1140.9 | Jaroszewska and Wilczyńska, 2006 |
| Tree shrew (*Tupaia Javanica*) | 0.075 | 0.608 | 810.7 | Kakuni et al., 2002 |
| Aberts squirrel (*Sciurus aberti*) | 0.5117 | 1.666 | 325.6 | Murphy and Linhart, 1999 |
| Fox squirrel (*Sciurus niger*) | 0.5322 | 1.44 | 270.6 | Murphy and Linhart, 1999 |
| Eastern grey squirrel *(Sciurus carolinensis*) | 0.523 | 2.12 | 405.4 | Murphy and Linhart, 1999 |
| White-faced flying squirrel (Petaurista alborufus lena) | 4.29 | 1.83 | 42.7 | Liu et al., 2017 |
| Ganges dolphin (*Platanista gangetica*) | 148 | 4.91 | 3.32 | Takahashi and Yamasaki, 1972 |
| Amazon river dolphin (*Inia geoffrensis*) | 185 | 41.5 | 22.4 | Yamasaki and Kito, 1984 |
| Southern minke whale (*Balaenoptera bonaerensis*) | 2400 | 35.4 | 1.47 | Pérez et al., 2017 |

**Supplementary Table13.** Small intestine length and body weight in birds

| Species | Body weight g | Small intestine | | Reference |
| --- | --- | --- | --- | --- |
|  |  | cm | cm kg^-1^ |  |
| Ostrich | 90000 | 434.7 | 4.83 | Yang et al., 2013 |
| Canvasback (*Aythya valisineria*) | 1206 | 139.6 | 168.4 | Kehoe and Ankner, 1985 |
| Great scaup (*Aythya marila*) | 908 | 169.6 | 186.8 | Kehoe and Ankner, 1985 |
| Lesser scaup (*Aythya affinis*) | 731 | 172.1 | 235.4 | Kehoe and Ankner, 1985 |
| Mallard/domestic duck | 2200 | 179.1 | 81.4 | Kokoszyński et al., 2018 |
| Redhead (*Aythya americana*) | 997 | 149.5 | 149.9 | Kehoe and Ankner, 1985 |
| Ring-necked duck (*Aythya collaris*) | 749 | 118 | 157.5 | Kehoe and Ankner, 1 |
| Tufted duck (*Aythya fuligula*) | 1085 | 174.8 | 161.1 | Działa-Szczepańczyk and Wesołowska, 2008 |
| Japanese Quail (*Coturnix japanica*) | 93.6 | 55.6 | 594 | Hena et al., 2012 |
| Laying hen (chicken) | 1600^A^ | 132.8 | 83 | Ding et al., 2018 |
| Goshawk (*Accipiter gentilis*) | 219 | 43.6 | 199.1 | Barton, 1992 |
| Sparrowhawk (*Accipiter niuss*) | 182 | 72.1 | 396.2 | Barton, 1992 |
| Buzzard (*Buteo buteo*) | 848 | 103.1 | 121.6 | Barton, 1992 |
| Red kite (*Milvus milvus*) | 1033 | 105.2 | 101.8 | Barton, 1992 |
| Pigeon (*Columbia livia*) | 79.6 | 6.11 | 76.8 | Hena et al., 2012 |
| Peregrine (*Falco peregrinus*) | 762 | 69.7 | 91.5 | Barton, 1992 |
| Kestrel (*Falco tinnunculus*) | 162 | 54.0 | 333.3 | Barton, 1992 |
| Merlin (*Falco columbarius*) | 156 | 48.9 | 313.5 | Barton, 1992 |
| Houbara bustard (*Chlamydotis undulata*) | 1143 | 37.1 | 32.5 | Bailey et al., 1997 |
| Kori bustard (*Ardeotis kori*) | 7274 | 107.7 | 14.8 | Bailey et al., 1997 |
| Rufous-crested Bustard (*Eupodotis ruficrista*) | 509 | 38.0 | 74.7 | Bailey et al., 1997 |
| White-bellied Bustard (*Eupodotis senegalensis*) | 944 | 41.0 | 43.4 | Bailey et al., 1997 |
| Redstart (*Setophaga ruticilla*) | 7.6 | 9.94 | 1307 | Stevenson, 1933 |
| Blackburnian warbler (*Setophaga fusca*) | 10.3 | 10.06 | 977 | Stevenson, 1933 |
| Eastern house wren (*Troglodytes aedon*) | 11.4 | 11.0 | 964 | Stevenson, 1933 |
| Black-capped chickadee (*Poecile atricapillus*) | 11.4 | 12.8 | 1125 | Stevenson, 1933 |
| Eastern chipping sparrow (*Spizella passerina*) | 11.6 | 12.6 | 1143 | Stevenson, 1933 |
| Eastern field sparrow (*Spizella pusilla*) | 13.2 | 13.2 | 999 | Stevenson, 1933 |
| Red-eyed vireo (*Vireo olivaceus*) | 16.3 | 15.1 | 925 | Stevenson, 1933 |
| Ovenbird (*Seiurus aurocapilla*) | 19.2 | 16.8 | 875 | Stevenson, 1933 |
| Song sparrow (*Melospiza melodia*) | 19.3 | 17.7 | 918 | Stevenson, 1933 |
| Northern whitebreasted nuthatch *(Sitta carolinensis*) | 20.6 | 14.9 | 724 | Stevenson, 1933 |
| Slate-colored junco (*Junco hyemalis*) | 21.2 | 18.8 | 885 | Stevenson, 1933 |
| Eastern purple finch (*Haemorhous purpureus*) | 25.3 | 34.5 | 1362 | Stevenson, 1933 |
| English sparrow (*Passer domesticus*) | 26.7 | 15.7 | 587 | Stevenson, 1933 |
| Eastern hermit thrush (*Catharus guttatus*) | 32 | 16.1 | 504 | Stevenson, 1933 |
| Eastern catbird (*Dumetella carolinensis*) | 34.6 | 19.0 | 548 | Stevenson, 1933 |
| Red-eyed towhee (*Pipilo erythrophthalmus*) | 40.7 | 21.9 | 537 | Stevenson, 1933 |
| Eastern cowbird (*Molothrus ater*) | 44.2 | 15.8 | 357 | Stevenson, 1933 |
| Starling (*Sturnus vulgaris*) | 79.5 | 29.2 | 367 | Stevenson, 1933 |
| Eastern robin (*Turdus migratorius*) | 80.5 | 26.6 | 331 | Stevenson, 1933 |
| Bronzed grackle (*Quiscalus quiscula*) | 104.6 | 29.1 | 278 | Stevenson, 1933 |
| Redstart (*Setophaga ruticilla*) | 7.6 | 10.1 | 1307 | Stevenson, 1933 |
| Blackburnian warbler (*Setophaga fusca*) | 10.3 | 10.1 | 977 | Stevenson, 1933 |
| African pied crow (*Corvus albus*) | 520 | 67.9 | 130.4 | Okpe et al., 2016 |
| Antarctic prion (*Pachyptila desolata*) | 147 | 44 | 299.3 | Olsen et al., 2002 |
| Manx shearwater (*Puffinus puffinus*) | 453 | 59 | 130.2 | Olsen et al., 2002 |
| Northern fulmar (*Fulmarus glacialis*) | 544 | 136 | 250 | Olsen et al., 2002 |
| Southern giant petrel (*Macronectes giganteus*) | 4600 | 239 | 52.0 | Olsen et al., 2002 |
| Little penguin (*Eudytula minor*) | 1100 | 148 | 134.5 | Olsen et al., 2002 |
| Rockhopper penguin (*Eudytula chrysocome*) | 2500 | 488 | 195.2 | Olsen et al., 2002 |
| Jackass penguin *(Spheniscus demersus*) | 3100 | 559 | 180.3 | Olsen et al., 2002 |
| Macaroni penguin (*Eudytula chrysolophus*) | 3900 | 633 | 162.3 | Olsen et al., 2002 |
| Magellanic penguin *(Spheniscus magellanicuss*) | 4500 | 873 | 194 | Olsen et al., 2002 |
| Adélie penguin (*Pygoscelis adeliae*) | 4900 | 297 | 60.6 | Olsen et al., 2002 |
| King penguin (*Aptenodytes patagonicus*) | 13200 | 686 | 52.0 | Olsen et al., 2002 |
| Emperor penguin (*Aptenodytes forsteris*) | 27600 | 618 | 22.4 | Olsen et al., 2002 |
| Long eared owl (*Asio otus*) | 333 | 44.2 | 132.7 | Barton, 1992 |
| Barn owl (*Tyto alba*) | 270 | 37.7 | 139.6 | Barton, 1992 |
| Short eared owl (*Asio flammeus*) | 294 | 58.1 | 197.6 | Barton, 1992 |
| Tawny owl (*Strix aluco*) | 449 | 63.9 | 142.3 | Barton, 1992 |

**Supplementary Table14.** Lengths/height (in μm) of villi in the small intestine in birds and mammals

| Species | Duodenum | Jejunum | Ileum | Reference |
| --- | --- | --- | --- | --- |
| Human *(Homo sapiens*) | 704 |  |  | Hasan and Ferguson, 1981 |
| African green monkey (*Chlorocebus aethiops*) | 605 | 663 | 398 | Paulini et al., 2006 |
| Common shrew (Sorex araneus) | 416 | 442 | 356 | Jaroszewska and Wilczyńska, 2006 |
| Guinea pig (*Cavia porcellus*) |  | 579 |  | Weaver and Carrick, 1989; Mitjans, and Ferrer, 2004 |
| Ground squirrel (*Spermophilus tridecemlineatus*) |  | 529 |  | Carey, 1990 |
| Persian squirrel (*Sciurus anomalus*) | 297 | 208 | 203 | Tootian et al., 2013 |
| House mouse (*Mus musculus*) | 386 | 346 | 156 | Short and Derrickson, 2020 |
| Cattle (*Bos taurus*) | 855 | 862 | 583 | Zitnan et al., 2008; Kvidera et al., 2017 |
| Brown rat (*Rattus norvegicus*) | 504 | 416 | 247 | Gardner and Steele, 1989; Seyyedin and Nazem, 2017 |
| Dog (*Canis lupus*) | 739 | 653 | 572 | Verma et al., 1968; Kuzmuk et al., 2005 |
| Pig (*Sus scrofa*) | 663 | 588 | 447 | Kitt et al., 2002; Chwen et al., 2013 |
|  |  |  |  |  |
| Chicken (*Gallus gallus*) | 1357 | 960 | 721 | Means over multiple studies |
| Duck (*Anas platyrhynchos*) | 1086 | 939 | 699 | Ran et al., 2020 |
| Turkey (*Meleagris gallopavo*) | 968 | 448 | 263 | Girish and Smith, 2008 |
| Semipalmated sandpiper (Calidris pusilla) | 459 | 459 | 459 | Chaves et al., 2018 |

^R^ The height/length of the villi in the duodenum of rabbits (3141 μm) was excluded being outside the mean plus 5 x SD (Oliveira et al., 2013).

**Supplementary Table 15. Glomerular filtration rate in avian and mammalian species**

|  | Body weight  (kg) | Glomerular filtration rate (GFR)  ml min^-1^ | GFR  ml min^-1^ kg^-1^ | Reference |
| --- | --- | --- | --- | --- |
| Class **Mammalia** |  |  |  |  |
| American black bear (*Ursus americanus*) | 62.8 | 117 | 1.86 | Brown et al., 1971 |
| Bottle nose dolphin (*Tursiops gilli*) | 159.1 | 465 | 2.92 | Malvin, and Rayner, 1968;  Ven Watson et al., 2007 |
| Cat (*Felis catus*) | 4.4^#^ | 12.7 | 2.88 | Von Hendy-Willson |
| Cattle (*Bos taurus*) | 85 | 230.3 | 2.71 | Imai et al., 2012 |
| Common bottle nose dolphin (*Tursiops truncatus*) | 12^3##^ | 188 | 1.53 | Ven Watson et al., 2007 |
| Cynomolgus monkey (*Macaca fascicularis*) | 4.4 | 11.88 | 2.70 | Iwama et al., 2014 |
| Donkey (*Equus asinus*) | 165^#^ | 297 | 1.80 | Meucci et al., 2015 |
| Ferret (*Mustela putorius*) | 2.37^#^ | 7.87 | 3.32 | Esteves et al., 1994 |
| Goat (*Capra aegagrus*) | 11 | 20.2 | 1.84 | Mishra et al., 2013 |
| Guinea pig (*Cavia porcellus*) | 1.02^#^ | 0.48 | 0.47 | Johnson and Spitzer, 1986 |
| Horse (*Equus caballus*) | 450^#^ | 1161 | 2.58 | Meucci et al., 2015 |
| House mouse (*Mus musculus*) | 0.0193 | 0.19 | 9.80 | Sasaki et al., 2014 |
| Pig (*Sus scrofa*) | 13.9 | 59.9 | 4.31 | Gasthuys et al., 2017 |
| Rabbit (*Oryctolagus cuniculus*) | 3.85^#^ | 15.4 | 4.01 | Michigoshi et al., 2011 |
| Brown rat (*Rattus norvegicus*) | 0.23^#^ | 0.39 | 1.70 | Katayama et al., 2010 |
| Rough toothed dolphin (*Steno bredinensi*s) | 131 | 54.5 | 2.40 | Malvin, and Rayner, 1968 |
| Sheep (*Ovis aries*) | 50 | 118 | 1.76 | Luis-Lima et al., 2020 |
| Squirrel monkey (*Saimiri sciureus*) | 0.656 | 1.45 | 2.21 | Maddox et al., 1974 |
| Class **Aves** |  |  |  |  |
| **Order *Anseriformes*** |  |  |  |  |
| Barrow’s goldeneye (*Bucephala islandica*) | 0.767 | 4.06 | 5.29 | From summary in Bennett and Hughes, 2003 |
| Canada goose (*Branta canadensis*) | 3.900 | 4.68 | 1.20 | **Calculated from Hughes,** 1980 |
| Canvasback duck (*Aythya valisineria*) | 1.052 | 2.27 | 2.16 | From summary in Bennett and Hughes, 2003 |
| Mallard duck (Anas platyrhynchos) | 0.983 | 2.6 | 2.64 | Hughes et al., 1999, Bennett & Hughes, 2003 |
| Muscovy duck (*Cairina moschata*) | 2.590 | 6.73 | 2.60 | Gasthuys et al., 2019 |
| **Order *Apodiformes*** |  |  |  |  |
| Anna's hummingbird (*Calypta anna*) | 0.0051 | 0.040 | 7.84 | From summary in Bennett and Hughes, 2003 |
| Broad-tailed hummingbird (*Selasphorus platycercus*) | 0.00365 | 0.027 | 7.40 | Hartman Bakken et al., 2004; Bakken et al., 2004 |
| Green-backed firecrown (*Sephanoides sephanoides*) | 0.00531 | 0.033 | 6.21 | Hartman Bakken and Sabat, 2006 |
| Order ***Charadriiformes*** |  |  |  |  |
| Glaucous-winged (*Larus glaucescens*) | 0.900 | 1.54 | 1.71 | **Calculated from Hughes,** 1980 |
| Herring gull (*Larus argentatus*) | 0.900 | 4.40 | 4.89 | From summary in Bennett and Hughes, 2003 |
| Kelp gull (*Larus dominicanus*) | 0.905 | 3.09 | 3.41 | From summary in Bennett and Hughes, 2003 |
| Order **Columbiformes** |  |  |  |  |
| Pigeon (*Columbia livia*) | 0.426 | 2.35 | 5.52 | Chan et al. 1972, Giladi et al., 1997 |
| Mourning Dove  (*Zenaidura macroura*) | 0.119 | 0.27 | 2.27 | From summary in Bennett and Hughes, 2003 |
| Order ***Falconiformes*** |  |  |  |  |
| American kestrel (Falco sparverius) | 0.126 | 0.27 | 2.14 | From summary in Bennett and Hughes, 2003 |
| Order ***Galliformes*** |  |  |  |  |
| Bobwhite quail (*Colinus virginus*) | 0.173 | 0.68 | 3.93 | Goldstein, 1995 |
| Chicken (*Gallus gallus*) | 1.660 | 3.89 | 2.34 | Adult from summary in (Bennett and Hughes, 2003) adults and in young broiler chickens (Wideman and colleagues) |
| Chukar partridge (*Alectoris chukar*) | 0.512 | 0.58 | 1.13 | Goldstein, 1990 |
| Desert quail (*Lophortyx gambelii*) | 0.150 | 0.132 | 0.88 | Braun and Dantzler, 1972 |
| Gambel’s quail (*Callipepia gambelii*) | 0.158 | 0.34 | 2.15 | Williams et al.,1991; Williams and Braun, 1996; |
| Japanese quail (*Coturnix japonica*) | 0.122 | 1.55 | 12.70 | Roberts and Hughes, 1983 |
| King quail (*Coturnix chinensis* or  *Excalfactoria chinensis*) | 0.053 | 0.56 | 10.57 | Roberts et al., 1985 |
| Stubble quail (*Coturnix pectoralis*) | 0.100 | 0.80 | 8.00 | Roberts et al., 1985 |
| Turkey *(Meleagris pavo*) | 7.40 | 5.68 | 0.77 | From summary in Bennett and Hughes, 2003 |
| Order ***Passeriformes*** |  |  |  |  |
| European starling (*Sturnus vulgaris*) | 0.0771 | 0.50 | 6.49 | From summary in Bennett and Hughes, 2003 |
| House sparrow (Passer domesticus) | 0.0228 | 0.13 | 5.70 | Goldstein and Braun, 1988 |
| New Holland honeyeater (*Phylidonyris novaehollandiae)* | 0.0226 | 0.095 | 4.20 | Purchase et al., 2013 |
| Palestine sunbirds (*Nectarinia osea*) | 0.00581 | 0.033 | 5.68 | McWhorter et al., 2004 |
| Red wattle bird (Anthochaera carunculata) | 0.099 | 0.33 | 3.33 | Goldstein and Bradshaw, 1998 |
| Song sparrow (Melospiza melodia) | 0.0184 | 0.13 | 7.07 | Goldstein and Rothschild,1993 |
| Swainson’s thrush (Catharus ustulatus) | 0.0278 | 0.47 | 16.912 | Gerson and Guglielmo, 2013 |
| Whitebellied sunbird (*Cinnyris talatala*) | 0.00807 | 0.023 | 2.86 | Purchase et al., 2013 |
| Order ***Psittaciformes*** |  |  |  |  |
| Budgerigar (*Melopsittacus undulates*) | 0.0375 | 0.14 | 3.73 | From summary in Bennett and Hughes, 2003 |
| Galah (*Cacatua roseicapilla*) | 0.3357 | 0.79 | 2.35 | From summary in Bennett and Hughes, 2003 |
| African grey parrot (*Psittacus erithacus*) | 0.470 | 0.52 | 1.11 | Gasthuys et al., 2019 |
| Order ***Struthioniformes*** |  |  |  |  |
| Emu (*Dromaius novaehollandiae*) | 40.700 | 16.2 | 0.40 | From summary in Bennett and Hughes, 2003 |

# estimated ## Body weight calculated from data in Pillay and Manger, 2004.

**REFERENCES FOR SUPPLEMENTARY DATA**

Abood, D.A., Reshag, A.F., Azhar, S.K. and Ahmed, M.A. 2014. Comparative anatomical and histological features of the kidney in harrier (*Circus aueroginosus*), chicken (*Gallus domesticus*) and Mallard duck (*Anas platyrhynchos*). Iraqi J. Vet. Med. 38, 107-113.

Agnvall, B., Bélteky, J. and Jensen, P. 2017. Brain size is reduced by selection for tameness in Red Junglefowl– correlated effects in vital organs. Sci. Rep. 7, 3306.

Ahlers, A.A., Mitchell, M.A., Schooley, R.L., Heske, E.J. and Levengood, J.M. 2011. Hematologic and blood chemistry reference values for free-ranging muskrats (Ondatra zibethicus). J. Wildl. Dis. 47, 685-689.

Akiba, Y., Jensen, L.S., Barb, C.R. and Kraeling, R.R. 1982. Plasma estradiol, thyroid hormones, and liver lipid content in laying hens fed different isocaloric diets. J. Nutr. 112, 299–308.

Alagaili, A.N., Omer, S.A., Bray, T.C., Mohammed, O.B. 2013. Reference data of haematology and serum biochemistry in adult wild-caught Libyan jird (*Meriones libycus*) from central Saudi Arabia. Journal of King Saud University - Science 25, 307-311.

Albers, P.H., Klein, P.N., Green, D.E., Melancon, M.J., Bradley, B.P. and Noguchi, G. 2006. Chlorfenapyr and mallard ducks: overview, study design, macroscopic effects, and analytical chemistry. Environ. Toxicol. Chem. 25, 438-445.

Aljumaah, R.S. and Hussein, M.F. 2011. Haematological, hemastatic and blood chemical values of captive Erlanger’s gazelles (*Gazalla erlangeri*). J. Anim. Vet. Adv. 10, 1699-1705.

Al-kahtani, M. A., Zuleta, C., Caviedes-Vidal, E. and Garland, T. Jr. 2004. Kidney mass and relative medullary thickness of rodents in relation to habitat, body size, and phylogeny. Physiol. Biochem. Zool. 77, 346–365. Appendix A

Andraszek, K., Wójcik, E., Gruzewska, A. and Smalec, E. 2009. Genome size of the European domestic goose (*Anser anser domesticus*). Can. J. Anim. 89, 449-455.

Andriansyah, Candra, D., Riyanto, M.A., Barry, J. and Radcliffe, R.W. 2013. Hematology and serum biochemistry of Sumatran rhinoceroses (*Dicerorhinus sumatrensis*) in a rainforest sanctuary in Way Kambas National Park, Indonesia. J. Zoo Wildl. Med. 44, 280-284.

Araújo, P.M., Viegas, I., Rocha, A.D., Villegas, A., Jones, J.G., Mendonça, L., Ramos, J.A., Masero, J.A. and Alves, J.A. 2019. Understanding how birds rebuild fat stores during migration: insights from an experimental study. Scientific Reports 9, 10065.

Aroch, I., Shpigel, N.Y., Avidar, Y., Yakobson, B., King, R. and Shamir, M. 2005. Haematological and biochemical measurements in healthy, adult, free-ranging golden jackals (*Canis aureussyriacus*) held in captivity. Vet. Rec.157, 317-321.

Aroch I, King R, Baneth G. 2007. Hematology and serum biochemistry values of trapped, healthy, free-ranging rock hyraxes (*Procavia capensis*) and their association with age, sex, and gestational status. Vet. Clin. Pathol. 36, 40-48.

Arora, K. and Samples, O. 2011. Role of body weight on reproductive and physiological traits in Japanese quail layers (*Coturnix japonica*). Int. J. Poult. Sci. 10, 640-643.

Bacon, W. L. 1981. Metabolism of lipid labeled very low density lipoprotein from laying turkey hens in laying turkey hens and immature turkeys. Poult. Sci. 60, 1525-1536.

Bacon, W. L. 1974. Plasma free fatty acid and neutral lipid concentrations in immature, laying and broody turkey hens. Poult. Sci. 53, 1154-1160.

Bailey, T.A., Mensah-Brown, E.P., Samour, J.H., Naldo, J., Lawrence, P., Garner, A. 1997. **Comparative morphology of the alimentary tract and its glandular derivatives of captive bustards.** J. Anat. 191: 387-398.

Barnes, T.S., Goldizen, A.W. and Coleman, G.T. 2008. Hematology and serum biochemistry of the brush-tailed rock-wallaby (*Petrogale penicillata*). J. Wildl. Dis. 44, 295-303.

Barton, N.W.H. 1992. Morphological adaptation and digestion in relation to raptor feeding ecology. Unpublished Ph.D. thesis, University of Glasgow.

Barton, N.W. and Houston, D.C. 1996. Factors influencing the size of some organs in some raptors. J. Raptor Res. 30, 219-223.

Bechert, U., Mortenson, J., Dierenfeld, E.S., Cheeke, P., Keller, M., Holick, M., Chen, T.C. and Rogers, Q. 2002. Diet composition and blood values of captive cheetahs (*Acinonyx jubatus*) fed either supplemented meat or commercial food preparations. J. Zoo. Wildl. Med. 33, 16-28.

Beechler, B.R., Jolles, A.E. and Ezenwa, V.O. 2009. Evaluation of hematologic values in free-ranging African buffalo (*Syncerus caffer*). J. Wildl. Dis. 45, 57-66.

Belcher, C., Heatley, J.J., Petzinger, C., Hoppes, S., Larner, C.D., Sheather, S.J. and Macfarlane, R.D. 2014. Evaluation of plasma cholesterol, triglyceride, and lipid density profiles in captive monk parakeets (*Myiopsitta monachus*). J. Exot. Pet. Med. 23, 71-78.

Bernard, S.F., Fayolle, C., Robin, J.-P. and Groscolas, R. 2002. Glycerol and NEFA kinetics in long-term fasting king penguins: phase II versus phase III. J. Exp. Biol. 205, 2745–2754.

Beltrán F. S. L., Nallar R. G., Villalba L. M. M., Delgado E. E. and Berna M. M. 2009. Inmovilización química, evaluación hematológica y coproparasitología de *Leopardus colocolo* en Khastor, Potosí, Bolivia: Chemical Immobilization and hematologyc and Endoparasitologic Evaluation of *Leopardus colocolo* in Khastor, Potosí, Bolivia. *Rev. investig*. *vet. Perú* **20** <http://www.scielo.org.pe/scielo.php?script=sci_arttext&pid=S1609-91172009000200022> accessed 1.19.14

Benn, D.M., McKeown, D.B. and Lumsden, J.H. 1986. Hematology and biochemistry reference values for the ranch fox. Can. J. Vet. Res. 50, 54–58.

Bhatavdekar, J.M., Babu, K.A. and Shah, V.C. 1977. Effects of low dose x-radiation on nucleic acids and protein content of guinea pig, rat and mouse tissues. Indian J. Exp. Biol.15, 980-985.

Black, D.M., Gilardi, K.V., Hamilton, L.P., Williams, E., Williams, D.F., Kelly, P.A. and Gardner, I. 2009. Hematologic and biochemistry reference values for the endangered riparian brush rabbit (*Sylvilagus bachmani riparius*). J. Wildl. Dis. 45, 491-496.

Boily, F., Beaudoin, S. and Measures, L.N. 2006. Hematology and serum chemistry of harp (*Phoca groenlandica*) and hooded seals (*Cystophora cristata*) during the breeding season, in the Gulf of St. Lawrence, Canada. J. Wildl. Dis. 42, 115-132.

Borin, K., Lindberg, J.E. and Ogle, R.B. 2006. Digestibility and digestive organ development in indigenous and improved chickens and ducks fed diets with increasing inclusion levels of cassava leaf meal. J. Anim. Physiol. Anim. Nutr. (Berl.) 90, 230-237.

Brown, D.C., Mulhausen, R.O., Andrew, D.J. and Seal, U.S. 1971. Renal function in anesthetized dormant and active bears. Am. J. Physiol. 220, 293–298.

Brüssow, K.-P., Egerszegi, I., Rátky, J., Soós, F., Casado, P.G., Tuchscherer, A. and Toth, P. 2004. Organometric data of the reproductive tract in cycling and early pregnant Hungarian Mangalica pigs. Archiv. fur Tierzucht 47, 585-594.

Bulla, J., Granát, J., Zelník, J. and Palanská, O. 1974. DNA and RNA concentration in the liver of Japanese quail (*Coturnix coturnix japonica*) during growth. Experientia 30, 371-372.

Bulla, J., Zelník, J., Granat, J. and Dobálová, M. 2010. DNA and RNA concentration in the liver of geese during postnatal growth. J. Anim. Breed. Genet. 97, 81 - 85.

Burrin, D.G., Britton, R.A., Ferrell, C.L. and Bauer, M.L. 1992. Level of nutrition and visceral organ protein synthetic capacity and nucleic acid content in sheep. J. Anim. Sci. 70:1137-1145.

Buse, E., Zöller, M. and Van Esch, E. 2008. The macaque ovary, with special reference to the cynomolgus macaque (*Macaca fascicularis*). Toxicol. Pathol., 36: 24S-66S.

Bush, M., Custer, R.S. and Smith, E.E. 1980. Use of dissociative anesthetics for the immobilization of captive bears: blood gas, hematology and biochemistry values. [J. Wildl. Dis.](http://www.ncbi.nlm.nih.gov/pubmed/7463600%22%20%5Co%20%22Journal%20of%20wildlife%20diseases.) 16, 481-489.

**Campbell, K.L**, **Storz, J.F.**, **Signore, A.V.**, **Moriyama**, **H**, **Catania**, **K.C.**, **Payson** **A.P.**, **Bonaventura**, **J.**, **Stetefeld**, **J.** and **Weber**, **R.E.** 2010. Molecular basis of a novel adaptation to hypoxic-hypercapnia in a strictly fossorial mole. BMC Evolutionary Biology **10**, 214.

Canales-Espinosa, D., Rovirosa-Hernández M.d.J., Thoisy, B.d., Caba, M. and García-Orduña, F. 2020. Hematology an Serum Biochemistry in wild Howler Monkeys. In: Hematology an Serum Biochemistry in wild Howler Monkeys. In: Developments in Primatology: Progress and Prospects. Howler Monkeys Adaptive Radiation, Systematics, and Morphology pp. 179-200, Eds: M.M. Kowalewski, L. Cortés-Ortiz, D. Youlatos, P.A. Garber and B. Urbani,. Springer New York.

Carey, H.V. 1990. Seasonal changes in mucosal structure and function in ground squirrel intestine. Am. J. Physiol. 259, R385–R392.

[Casanave](http://link.springer.com.ezproxy.lib.uwm.edu/search?facet-author=%22Dra.+E.+B.+Casanave%22), E. B. and [Polini](http://link.springer.com.ezproxy.lib.uwm.edu/search?facet-author=%22N.+N.+Polini%22). N. N. 1999. Comparative study of some haematological parameters of two wild Chaetophractus villosus (mammalia, dasypodidae) populations. Comp Haematol Int. 9, 13-16.

Casas-Díaz, E., López-Olvera, J.R., Marco, I., Mentaberre, G. and Lavín, S. 2008. Hematologic and biochemical values for Spanish ibex (*Capra pyrenaica*) captured via drive-net and box-trap. [J. Wildl. Dis.](http://www.ncbi.nlm.nih.gov/pubmed/?term=HEMATOLOGIC+AND+BIOCHEMICAL+VALUES+FOR+SPANISH+IBEX+(CAPRA+PYRENAICA)+CAPTURED+VIA+DRIVE-NET+AND+BOX-TRAP%22%20%5Co%20%22Journal%20of%20wildlife%20diseases.) 44, 965-972.

[Castellanos, A](javascript:__doLinkPostBack('','ss~~AR%20%22Castellanos%2C%20Armando%22%7C%7Csl~~rl','');)., [Arias, L.,](javascript:__doLinkPostBack('','ss~~AR%20%22Arias%2C%20Leonardo%22%7C%7Csl~~rl','');) [Jackson, D](javascript:__doLinkPostBack('','ss~~AR%20%22Jackson%2C%20David%22%7C%7Csl~~rl','');)., and [Castellanos, R](javascript:__doLinkPostBack('','ss~~AR%20%22Castellanos%2C%20Roberto%22%7C%7Csl~~rl','');). 2010. Hematological and serum biochemical values of Andean bears in Ecuador**.** [Ursus](javascript:__doLinkPostBack('','mdb~~aph%7C%7Cjdb~~aphjnh%7C%7Css~~JN%20%22Ursus%22%7C%7Csl~~jh','');) 21 115-120.

Castellini, M.A., Baskurt, O., Castellini, J.M., and Meiselman, H.J. 2010. [Blood rheology in marine mammals.](http://www.ncbi.nlm.nih.gov/pubmed/21423386) Front. Physiol.1:146.

Catley, A., Kock, R.A., Hart, M.G. and Hawley, C.M. 1990. Haematology of clinically normal and sick captive rein deer (*Rangifer tarandus*). Vet. Rec. 126, 239-241.

Center, S.A., Warner, K.L. and Erb, H.N. 2002. Liver glutathione concentrations in dogs and cats with naturally occurring liver disease. Am. J. Vet. Res. 63, 1187-1197.

Çetin, Silici, E., Çetin, N. and Güçlü, B.K. 2010. Effects of diets containing different concentrations of propolis on hematological and immunological variables in laying hens. Poult. Sci. 89, 1703-1708.

Chapple, R.S., English, A.W., Mulley, R.C. and Lepherd, E.E. 1991. Haematology and serum biochemistry of captive unsedated chital deer (*Axis axis*) in Australia. J. Wildl. Dis. 27, 396-406.

Challacombe, S.J. and Russell, M.W. 1979. Estimation of the intravascular half-lives of normal rhesus monkey IgG, IgA and IgM. Immunology 36, 331-338.

Chang, G.-R., Mao, F. C., Yang, C.-C. and Chan, F.-T. 2006. Hematological Profiles of the Formosan Black Bear (*Ursus thibetanusformosanus*). Zoological Studies 45, 93-97.

Chaves, F.Q., Neri, N.A., Rodrigues, R.C., Araujo, H.F.P. and Ricardo R. Guerra, R.R. 2018. Morphometry of pectoral muscle fiber and intestinal villi of *Calidris pusilla* during the wintering period in Brazil. Pesq. Vet. Bras. 38, 1849-1855.

Chege, S., Toosy A., Howlett, J., Saker, A. and Kagira, J. 2013. Haematology and biochemistry values of captive sand cats (Felis margarita) in Al Ain Wildlife Park and Resort, United Arab Emirates.J. Coastal Life Med., 1, 92-95

Chitwood, M.C., DePerno, C.S., Flowers, J.R. and Kennedy-Stoskopf, S. 2013. Physiological condition of female white-tailed deer in a nutrient-deficient habitat type. Southeast Naturalist 12, 307-316.

Chwen, L.T., Foo, H.L., Thanh, N.T. and Choe, D.W. 2013. Growth performance, plasma fatty acids, villous height and crypt depth of preweaning piglets fed with medium chain triacylglycerol. Asian-Australas J. Anim. Sci. 26, 700–704.

Clark, P. 2004. Haematology of Australian mammals. CSIRO Publishing, Collingwood, Victoria, Australia, pp. 1-250.

Clark, P., Holz, P., Booth, R., Jakob-Hoff, R., and Cooper, D.W.2003. Haematological characteristics of captive Parma wallabies (*Macropus parma*). Comp Clin Path 12, 11–16.

Crooks, K.R., Garcelon, D.K., Scott, C.A., Wilcox, J.T., Timm, S.F. and Van Vuren, D.H. 2003. Hematology and serum chemistry of the island spotted skunk on Santa Cruz Island. J. Wildl. Dis. 39, 460-466.

Cole, M.F. and Bowen, W.H. 1976. Immunoglobulins A, G, and M in serum and in some secretions of monkeys (*Macaca fascicularis* syn. *Irus*). Infect. Immun.13, 1354-1359.

Couch, C.E., Movius, M.A., Jolles, A.E., Gorman, M.E., Rigas, J.D. and Beechler, B.R. 2017. Serum biochemistry panels in African buffalo: Defining reference intervals and assessing variability across season, age and sex. PLoS ONE 12, e0176830.

Crile, G. and Quiring, G. 1940. A record of the body weight and certain organ and gland weights of 3690 animals. Ohio Journal of Science 40, 219-259.

Cui, Y.-m., Wang, J., Zhang, H.-j., Feng, J., Wu,, S.g., and Qi, G.-h. 2019. Effect of photoperiod on ovarian morphology, reproductive hormone secretion, and hormone receptor mRNA expression in layer ducks during the pullet phase. Poult. Sci. 98: 2439-2447.

Curtis, J. and Bourne, F.J. 1973. Half-Lives of immunoglobulins IgG, IgA and IgM in the serum of new-born pigs. Immunology 24, 147-155.

de Almeida Curi, N. H., Oliveira, P. A., Souto Lima, R. B., Gonçalves da Silveira, J. A., Costa Santos, J. L. and Chiarello, A. G. 2012. Haematology and several health aspects of endangered free-ranging thin-spined porcupines, *Chaetomys subspinosus* (Olfers, 1818) (Erethizontidae: Chaetomyinae). Comp. Clin .Pathol. 21, 1109–1113.

Deanesly, R. 1934. The reproductive processes of certain mammals. Part VI. The reproductive cycle of the female hedgehog. Philos. Trans. R. Soc. Lond. B 223, 239-276.

Debbie, J. G. and Clausen, B. (1975) Some hematological values of free-ranging African elephants. J. Wildl Dis. 11, 79-82.

Deem, S. L., Noss, A. J., Fiorello, C. V., Manharth, A. L., Robbins, R. G., and Karesh, W. B. 2009. Health assessment of free-ranging three-banded (*Tolypeutes matacus*) and nine-banded (*Dasypus novemcinctus*) armadillos in the Gran Chaco, Bolivia. J. Zoo. Wildl. Med. 40, 245–256.

DelGiudice, G.D., Krausman, P.R., Bellantoni, E.S., Wallace, M.C., Etchberger, R.C. and Seal, U.S. 1990. Blood and urinary profiles of free-ranging desert mule deer in Arizona. J. Wildl. Dis. 26, 83-89.

Deng, W., Dong, X.F., Tong J.M. and Zhang Q., 2012. The probiotic *Bacillus licheniformis* ameliorates heat stress-induced impairment of egg production, gut morphology and intestinal mucosal immunity in laying hens. Poult. Sci. 91: 575-582.

Diot, M., Reverchon, M., Rame, C., Froment, P., Brillard, J.-P., Brière, S., Levêque, G., Guillaume, D. and Dupont, J. 2015. Expression of adiponectin, chemerin and visfatin in plasma and different tissues during a laying season in turkeys. Reprod. Biol. Endocrinol. 13, 81.

Din, N. and Eltringham, S. 1977. Weights and measures of Ugandan pelicans with some seasonal variations. East Afr. Wildl. J. 15, 317-326.

Donaldson, J., Madziza, M.T. and Erlwanger, K.H. 2017. The effects of high-fat diets composed of different animal and vegetable fat sources on the health status and tissue lipid profiles of male Japanese quail (*Coturnix coturnix japonica*). Asian-Australas. J. Anim. Sci. 30, 700-711.

Drevemo, S., Grootenhuis, J. G. and Karstad, L. 1974. Blood parameters in wild ruminants in Kenya. J. Wildl. Dis. 10, 327-334.

Dutton, C.J., Dipl. A.C.Z.M., Randall E. Junge, R.E. and Edward E. Louis, E.E. 2008. Biomedical evaluation of free-ranging red ruffed lemurs (*Varecia rubra*) within the Masoala National Park, Madagascar. J. Zoo. Wildl. Med. 39, 76-85.

Działa-Szczepańczyk, E. and Wesołowska, I. 2008. **Morphometric characteristics of esophagus and intestine in tufted ducks (**Aythya fuligula**) wintering on the Baltic coastal areas in north-western Poland**. Electron. J. Polish Agric. Univ. 11, 1-35.

English, A.W., and Lepherd, E.E. 1981. The haematology and serum biochemistry of wild fallow deer (Dama dama) in New South Wales. J. Wildl. Dis. 17, 289-295.

Esteves, M.I., Marini, R.P., Ryden, E.B., Murphy, J.C. and Fox, J.G. 1994. Estimation of glomerular filtration rate and evaluation of renal function in ferrets (*Mustela putorius furo*). Am. J. Vet. Res. 55, 166-172.

Evans, K.D., Hewett, T.A., Clayton, C.J., Krubitzer, L.A. and Griffey, S.M. 2010. Normal organ weights, serum chemistry, hematology, and cecal and nasopharyngeal bacterial cultures in the gray short tailed opossum (*Monodelphis domestica*). [J. Am. Assoc. Lab. Anim. Sci.](http://www.ncbi.nlm.nih.gov/pubmed/?term=Normal+Organ+Weights,+Serum+Chemistry,+Hematology,+and+Cecal+and+Nasopharyngeal+Bacterial+Cultures+in+the+Gray+Short-Tailed+Opossum+(Monodelphis+domestica)%22%20%5Co%20%22Journal%20of%20the%20American%20Association%20for%20Laboratory%20Animal%20Science%20:%20JAALAS.) 49, 401-406

Fallaw, S.A., Jones, J.E. and Hughes, B.L. 1976. Hematocrit, erythrocyte, and hemoglobin values for male and female guineas at various ages. Poult. Sci. 55, 814-816.

Fayolle, C., Leray, C., Ohlmann, P., Gutbier, G., Cazenave, J.P., Gachet, C. and Groscolas, R. 2000. Lipid composition of blood platelets and erythrocytes of southern elephant seal (*Mirounga leonina*) and Antarctic fur seal (*Arctocephalus gazella*). [Comp. Biochem. Physiol. B](http://www.ncbi.nlm.nih.gov/pubmed/?term=Antarctic+Fur+Seal+fayolle+2000%22%20%5Co%20%22Comparative%20biochemistry%20and%20physiology.%20Part%20B,%20Biochemistry%20&%20molecular%20biology.) 126, 39-47.

Ferreyra, H., Beldomenico, P.M., Marchese, K., Romano, M., Caselli, A., Correa, A.I. and Uhart, M. 2015. Lead exposure affects health indices in free-ranging ducks in Argentina. Ecotoxicology 24, 735-745.

Fisher, H.I. and Bartlett, L.M. 1957. Diurnal cycles of liver weights in birds. The Condor 59, 364-372.

Fox, M., Brieva, C., Moreno, C., MacWilliams, P. and Thomas, C. 2008. Hematologic and serum biochemistry reference values in wild-caught White-Footed Tamarins (*Saguinus leucopus*) Housed in Captivity. J. Zoo. Wildl. Med. 39, 548-557.

Franzmann, A.W. 1971. Physiological values for Stone sheep. J. Wildl. Dis. 7, 139-141.

Fernández-Morán, J., Molina, L., Flamme, G., Saavedra, D. and Manteca-Vilanova, X. 2001. Hematological and biochemical reference intervals for wild caught Eurasian otter from Spain. J. Wildl Dis 37, 159-163.

Gabella, G. 1987. Numbers of neurons in the small intestine of mice, guinea-pigs and sheep. Neuroscience 22, 737-750.

Gardner, M.L.G. and Steele, D.L. 1989. Is there circadian variation in villus height in rat small intestine? Quart. J. Exp. Physiol. 74, 257-265.

Gasaway, W.C. and Buss, I.O. 1972. Zinc toxicity in the mallard duck. J. Wildl. Manage. 36, 1107-1117.

Gastal, G.D.A., Hamilton, A., Alves, B.G., de Tarso, S.G.S., Feugang, J.M., Banz, W.J., Apgar, G.A., Nielsen, C.K. and Gastal, E.L. 2017. Ovarian features in white-tailed deer (*Odocoileus virginianus*) fawns and does. PLoS ONE 12: e0177357.

Gasthuys, E., Devreese, M., Millecam, J., Sys, S., Vanderperren, K., Delanghe, J., Vande Walle, J., Heyndrickx, M. and Croubels, S. 2017. Postnatal maturation of the glomerular filtration rate in conventional growing piglets as potential juvenile animal model for preclinical pharmaceutical research. Front. Pharmacol. 8, 431.

Gasthuys, E., Montesinos, A., Caekebeke, N., Devreese, M., De Baere, S., Ardiaca, M., Paepe, D., Croubels, S. and Antonissen, G. 2019. Comparative physiology of glomerular filtration rate by plasma clearance of exogenous creatinine and exo-iohexol in six different avian species. Sci. Rep. 9, 19699.

Gates, N. L. and Goering, E. K. 1976. Hematologic values of conditioned, captive wild coyotes. J. Wildl Dis. 12, 402-404.

Gayathri, K. L. and Hegde, S. N. 2006. Alteration in haematocrit values and plasma

protein fractions during the breeding cycle of female pigeons, *Columba livia*. Anim.

Reprod. Sci. 91, 133-141.

Geraghty, D.P., Griffiths, J., Stewart, N., Robertson, I.K. and Gust, N. 2011. Hematologic, plasma biochemical, and other indicators of the health of Tasmanian platypuses (*Ornithorhynchus anatinus*): predictors of mucormycosis. J. Wildl. Dis. 47, 483-493.

Girish, C.K. and Smith, T.K. 2008. Effects of feeding blends of grains naturally contaminated with Fusarium mycotoxins on small intestinal morphology of turkeys. Poult. Sci. 87, 1075-1082.

Girling, S.J., Campbell-Palmer, R., Pizzi, R., Fraser, M.A., Cracknell, J., Arnemo, J. and Rosell, F. 2015. Haematology and serum biochemistry parameters and variations in the Eurasian beaver (*Castor fiber*). PLoS One 10, e0128775.

Gono, S. 1993. The domestication and nutrition of sambar deer (Cervus unicolor): a comparative study with red deer (*Cervus elaphus).* Ph.D. Thesis. Massey University.

Gonzalez-Quintela, A., Alende, R., Gude, F., Campos, J., Rey, J., Meijide, L.M., Fernandez-Merino, C. and Vidal, C. 2008. Serum levels of immunoglobulins (IgG, IgA, IgM) in a general adult population and their relationship with alcohol consumption, smoking and common metabolic abnormalities. Clin. Exp. Immunol. 151, 42–50.

Greig, D.J., Gulland, F.M.D., Rios, C.A. and Hall, A.J. 2010. Hematology and serum chemistry in stranded and wild-caught harbor seals in Central california: reference intervals, predictors of survival, and parameters affecting blood variables. J. Wildl. Dis. 46, 1172-1184.

Hagan, R.C., Leszczynski, D.E. and Kummerow, F.A. 1984. Comparative plasma lipid response of pullets and laying hens to estradiol and progesterone. Toxicol. Appl. Pharmacol. 76, 483-489.

Hanzal, V., Košinová, K., Pokorný, R., Janiszewski, P. and Hart, V. 2018. Weight parameters of body parts in sika deer (*Cervus nippon nippon*) from the Konstantinolázeňsko microregion, the Czech Republic. Cent. Eur. For. J. 64, 16–23.

Harms, N.J., Elkin, B.T., Gunn, A., Tracz, B., Adamczewski, J., Flood, P. and Leighton, F.A. 2012. Serum biochemistry and serum cortisol levels of immobilized and hunted muskoxen (*Ovibos moschatus*) from Northern Canada. Arctic 65, 401 – 410.

Harr, K.E., Reavill, D.R. Bursian, S.J., Cacela, D., Cunningham, F.L., Dean, K.M., Dorr, B.S.,

Hanson-Dorr, K.C., Healy, K., Horak, K., Link, J.E., Shriner, S. and Schmidt, R.E. 2017. Organ weights and histopathology of double-crested cormorants (*Phalacrocorax auritus*) dosed orally or dermally with artificially weathered Mississippi Canyon 252 crude oil. Ecotoxicol. Environ. Saf. 146, 52-61.

Harvey, J. W., Harr, K. E., Murphy, D., Walsh, M. T., Nolan, E. C., Bonde, R. K., Pate, M. G., Deutsch, C. J., Edwards, H. H. and Clapp, W. L. 2009. Hematology of healthy Florida manatees (*Trichechus manatus*). Vet. Clin. Pathol. 38, 183-193.

Harvey, S.B., Krimer, P.M., Correa, M.T. and Hanes, M.A., 2008.Hematology and plasma chemistry reference intervals for mature laboratory pine voles (*Microtus pinetorum*) as determined by using the nonparametric rank percentile method. J. Am. Assoc. Lab. Anim. Sci. 47, 35-40.

Hasan, M. and Ferguson, A. 1981. Measurements of intestinal villi in non-specific and ulcer-associated duodenitis correlation between area of microdissected villus and villus epithelial cell count. J. Clin. Pathol. 34, 1181-1186.

Hawkey, C.M., and Hart, M.G. 1985. Normal haematological values of axis deer (*Axis axis*), père davids deer (*Elaphus davidianus*) and barasingha (*Cervus duvauceli*). Res. Vet Sci. 39, 247-248.

Heard, D. J. and Whittier, D. A. 1997. Hematologic and plasma biochemical reference values for three flying fox species (*Pteropus* spp). J. Zoo Wildl. Med 28, 464–470.

Hegde, V.D. 2008. Effect of antiestrogen (tamoxifen citrate) on the ovary of the pigeon, *columba livia* during preincubation phase with reference to follicular growth. Asian J. Exp. Sci. 22, 89-94.

Hena, S.A., Sonfada, M.L., Bello, A., Umar, A.A. 2012. **Some comparative gross and morphometrical studies on the gastrointestinal tract in pigeon (**columbia livia**) and Japanese quail (**coturnix japonica**).** Sci. J. Vet. Adv. 1, 57-64.

Hermier, D., Guy, G., Guillaumin, S., Davail, S., André, J.-M. and Hoo-Paris, R. 2003. Differential channeling of liver lipids in relation to susceptibility to hepatic steatosis in two species of ducks. Comp. Biochem. Physiol. B 135, 663–675.

Hernandez-Divers, S. M., Aguilar, R., Leandro-Loria, D., and Foerste, C. R. 2005. Health evaluation of a radiocollared population of free- ranging Baird’s tapirs (*Tapirus bairdii*) in Costa Rica. J. Zoo Wildl. Med 36,176-187.

Hissa, R. 1997. Physiology of the European brown bear (Ursus arctos). Ann. Zool. Fennici 34, 267-287.

Holmes, W.N. and Stewart, D.J. 1968. Changes in the nucleic acid and protein composition of the nasal glands from the duck (*Anas platyrhynchos*) during the period of adaptation to hypertonic saline. J. Exp. Biol. 48, 509-519.

Horak, P., Jenni-Eiermann, S., Ots, I. and Tegelmann, L. 1998. Health and reproduction: the sex-specific clinical profile of great tits (*Parus major*) in relation to breeding. Can. J. Zool. 76, 2235.

Hrabčáková, P., Voslářová, E., Bedáňová, I., Pištěková, V., Chloupek, J. and Večerek, V. 2014. Haematological and biochemical parameters during the laying period in common pheasant hens housed in enhanced cages. Sci. World J. 2014: 364602.

Hunter, S.R. and Powers, L.R. 1980. Raptors hematocrit values. Condor 32, 226-227.

Hünigen, H., Mainzer, K., Hirschberg, R.M., Custodis, P., Gemeinhardt, O., Al Masri, S., Richardson, K.C., Hafez, H.M. and Plendl, J. 2016. Structure and age-dependent development of the turkey liver: a comparative study of a highly selected meat-type and a wild-type turkey line. Poult. Sci. 95, 901–911.

Ibañez, A.E., Najle, R., Larsen, K., Pari, M., Figueroa, A. and Montalti, D. 2015. Haematological values of three Antarctic penguins: gentoo (Pygoscelis papua), Adélie (P. adeliae) and chinstrap (P. antarcticus). Polar Res. 34, 25718.

Imai, K., Yamagishi, Y., Okura, N., Fukudac, T., Hirata, T., Okada, K., Sato, S. and Furuhama, K. 2012. Estimation of glomerular filtration rate in calves using the contrast medium iodixanol. Vet. J. 193, 174-179.

Inoue, H., Clifford, D.L., Vickers, T.W., Coonan, T.J., Garcelon, D.K. and Borjesson, D.L. 2012. Biochemical and hematologic reference intervals for the endangered island fox (*Urocyon littoralis*). J. Wildl Dis 48, 583-592.

Islam, M.R., Khandoker, M.A.M.Y., Afroz, S., Rahman, M.G.M. and Khan, R.I. 2007. Qualitative and quantitative analysis of goat ovaries, follicles and oocytes in view of in vitro production of embryos. J. Zhejiang Univ. Sci. B. 8: 465–469.

Islam, M.N., Zhu, X.B., Aoyama, M. and Sugita, S. 2010. Season morphological changes in the ovary of the Jungle crow (*Corvus macrorhynchos*). Anat. Sci. Int. 85, 224-234.

Ismoyowati, I. and Sumarmono, J. 2010. Fat and cholesterol contents of local duck (*Anas platyrhynchos platyrhynchos*) meat fed mash, paste and crumble feeds. Asian Journal of Poultry Science 5, 150-154

Iwama, R., Sato, T., Sakurai, K., Takasuna, K., Ichijo, T., Furuhama, K. and Satoh, H. 2014. Estimation of glomerular filtration rate in cynomolgus monkeys (*Macaca fascicularis*). J. Vet. Med. Sci. 76, 1423-1426.

Jacobson, H. A., Kirkpatrick, R. L., Burkhart, H. E. and Davis, W. 1978.Hematologic comparisons of shot and live trapped cottontail rabbits. J. Wildl Dis. 14, 82-88.

Jahantigh, M., Zaeemi, M., Razmyar, J. and Azizzadeh, M. 2019. Plasma biochemical and lipid panel reference intervals in common mynahs (*Acridotheres tristis*). J. Avian Med. Surg. 33, 15-21.

Jani, R.G., Sabapara, R.H., Bhuva, C.N. and Katatra, R.D. 2004. A study of reference intervals for the Asiatic wild ass *(Equus hemionus khur*). Zoos Print J. 19, 1332-1333.

Jaroszewska, M. and Wilczyńska, B. 2006. Dimensions of surface area of alimentary canal of pregnant and lactating female common shrews. J. Mammal. 87, 589–597.

Johns, J.T. and Bergen, W.G. 1976. Growth in Sheep. Pre - and Post-Weaning Hormone Changes and Muscle and Liver Development. J. Anim. Sci. 43, 192–200.

Johnson, O.W. 1973. Reproductive condition and other of shorebirds resident at Eniwetok during the boreal Summer. Condor 73, 336-343.

Johnson, V. and Spitzer, A.1986. Renal absorption of phosphate during development: whole-kidney events. Am. J. Physiol. 251, F251-F256.

Jones, K.R., Lall, K.R. and Garcia, G.W. 2019. Haematological and serum biochemical reference values of healthy Agoutis (*Dasyprocta leporina*) reared intensively in Trinidad, Republic of Trinidad and Tobago. Livestock Research for Rural Development 31.1.

Juráni, M., Výboh, P., Zeman, M., Lamosová, D., Kost’ál, L. Blazícek, P. 2004. Post-hatching dynamics of plasma biochemistry in free-living European starlings (*Sturnus vulgaris*). Comp. Biochem. Physiol. A 138, 89-95.

Kaabia, Z., Poirier, J., Moughaizel, M., Aguesse, A., Billon-Crossouard, S., Fall, F., Durand, M., Dagher, E., Krempf, M. and Croyal, M. 2018. Plasma lipidomic analysis reveals strong similarities between lipid fingerprints in human, hamster and mouse compared to other animal species. Sci. Rep. 8, 15893.

Kakuni, M., Makita, T., Wijayanto, H., Hondo, E. and Kiso, Y. 2002. Histological study of the intestinal diverticulum of tree shrew (*Tupaia Javanica*). Exp. Anim. 51, 411-415.

Kamiya, T., Uchida, S. and Kataoka, T. 1979. Organ weights of *Dugong dugon*. Sci. Rep. Whales Res. Inst. 31, 129-132

Kane, J.D., Steinbach, T.J., Sturdivant, R.X. and Burks, R.E. 2012. Sex-associated effects on hematologic and serum chemistry analytes in sand rats (*Psammomys obesus*). J. Am. Assoc. Lab. Anim. Sci. 51, 769–774.

Kapusta, A., Suh, A., and Feschotte, C. 2017. Dynamics of genome size evolution in birds and mammals. Proc. Natl. Acad. Sci. USA 114, E1460-E1469.

Kararli, T.L. 1995. Comparison of the gastrointestinal anatomy, physiology, and biochemistry of humans and commonly used laboratory animals. Pharmaceutics & Drug Disposition 16, 351-380.

Katayama, R., Yamaguchi, N., Yamashita, T., Watanabe, S., Satoh, H., Yamagishi, N. and Furuhama, K. 2010. Calculation of glomerular filtration rate in conscious rats by the use of a bolus injection of iodixanol and a single blood sample. J. Pharmacol. Toxicol. Methods 61, 59-64.

Kehoe, F.P. and Ankner, C.D. 1985. **Variation in digestive organ size among five species of diving ducks (*Aytha* spp.).** Can. J. Zool. 63, 2339-2342

Kennedy, J.P., Worthington, C.A. and Cole, E.R. 1974. The post-natal development of the ovary and uterus of the merino lamb. J. Reprod. Fert. 36, 275-282.

Keys, G. C., Fleischer, R.C. and Rothstein, S.I. 1986. Relationships between elevation, reproduction and the hematocrit level of brown-headed cowbirds. Comp. Biochem. Physiol. 83A, 765-769.

King, J.R., Follett, B.K., Farner, D.S. and Morton, M.L. 1966. Annual gonadal cycles and pituitary gonadotropins is *Zonotrichia leucophrys gambli*. Condor, 68, 476.

Kitt, S.J., Miller, P.S., Lewis, A.J. and Fischer, R.L. 2002. Effects of glutamine on growth performance and small intestine villus height in weanling pigs. Nebraska Swine Report. 29-32.

Kock, M. D., du Toit, R., Morton, D., Kock, N. and Paul, B. 1990. Baseline biological data collected from chemically immobilized, free-ranging black rhinoceroses (*Diceros bicornis*) in Zimbabwe. J. Zoo Wildl. Med. 21: 283–291.

Kohn, C.W., Knight, D., Hueston, W., Jacobs, R. and Reed, S.M. 1989. Colostral and serum IgG, IgA, and IgM concentrations in Standardbred mares and their foals at parturition. Am. Vet. Med. Assoc. 195, 64-68.

Kokoszyński, D., Saleh, M., Bernacki, Z., Kotowicz, M., Sobczak, M.,Żochowska-Kujawska, J. and Stęczny, K. 2018. Digestive tract morphometry and breast muscle microstructure in spent breeder ducks maintained in a conservation programme of genetic resources. Arch. Anim. Breed. 61, 373–378.

Kouamo, J., Dawaye, S.M., Zoli, A.P. and Bah, G.S. 2014. Evaluation of bovine (*Bos indicus*) ovarian potential for *in vitro* embryo production in the Adamawa plateau (Cameroon). Open Vet J. 4: 128–136.

Krapu, G.L. 1981. The role of nutrient reserves in mallard reproduction. Auk 98, 29-38.

Kusak, J., Rafaj, R.B., Zvorc, Z., Huber, D., Forsek, J., Bedrica, L. and Mrljak, V. 2005. Effects of sex, age, body mass, and capturing method on hematologic values of brown bears in Croatia. J. Wildl. Dis. 41, 843-847.

Kuzmuk, K.N., Swanson, K.S., Tappenden, K.A., Schook, L.B. and Fahey, G.C. 2005. Diet and age affect intestinal morphology and large bowel fermentative end-product concentrations in senior and young adult dog. J. Nutr. 135, 1940–1945.

Kvidera, S.K., Dickson, M.J., Abuajamieh, M., Snider, D.B., Sanz Fernandez, M.V., Johnson, J.S., Keating, A.F., Gorden, P.J., Green, H.B., Schoenberg, K.M. and Baumgard, L.H. 2017. Intentionally induced intestinal barrier dysfunction causes inflammation, affects metabolism, and reduces productivity in lactating Holstein cows. J. Dairy Sci. 100, 4113–4127.

Langer, S., Jurczynski, K. and Widmer, D. 2013. Hematologic and biochemical values in subadult and adult captive fossas (*Cryptoprocta ferox*). J. Zoo Wildl. Med. 44, 581-588.

Lautenschlager, R.A., Rothenbacher, H. and Podgwaite, J.D. 1979. Response of birds to aerial application of the nucleopolyhedrosis gypsy moth, *Lymantria dispar*. Environ. Entomol. 8, 760-764.

Lee, E. J. Moore, W. E., Fryer, H. C. and Minocha, H. C. 1982 Haematological and serum chemistry profiles of ferrets (*Mustela putorius furo*). Lab. Anim. 16, 133-137

Leat, W.M., Northrop, C.A., Buttress, N. and Jones, D.M. 1979. Plasma lipids and lipoproteins of some members of the order Perissodactyla. Comp. Biochem. Physiol B 63, 275-281.

Lewis, J. C. M., Norcott, M. R., Frost, L. M. and Cusdin, P. 2002. Normal haematological values of European hedgehogs (*Erinaceus europaeus*) from an English rehabilitation centre. Vet. Rec. 151: 567–569.

Li, M., Zhu, W., Wang, Y., Sun, Y., Li, J., Liu, X., Wu, Y., Gao, X. and Li, D. 2019. Effects of capture and captivity on plasma corticosterone and metabolite levels in breeding Eurasian Tree Sparrows. Avian Res. 10, 16.

Lien, T.-F., Lu, J.-J. and Jan, D.-F. 2001. Alterations in lipid metabolism between the growing and the laying periods of white Leghorn layers. Asian Australas. J. Anim. Sci. 14, 1460-1464.

Lien, J.-F., Chou, R.-C.R., Chen, S.-Y., Jeng, Y.-I. and Jan, D.-F. 1999. Lipid metabolism of Tsaiya ducks: plasma and liver related traits under *ad libitum* and fasting. Journal of the Science of Food and Agriculture 79, 1413-1416.

Lilburn, M.S. and Nestor, K.E. 1993. The relationship between various indices of carcass growth and development and reproduction in turkey hens. Poult. Sci. 72: 2030–2037.

Liu, X.-G., Zhang, Z.-Z., Zhang, Y.-H., Li, Y.-S., Fang, F.-G., Li, F.-G. and Yong Tao, Y. 2010. Oocyte and ovarian morphological observation of gray wolf (*Canis lupus*). Animal Biology 60, 249–257.

Liu, W.-M., Lai, S.J., Lu, L.-Z., Shi, F.-X., Zhang, J., Liu, Y., Yu. B., Tao, Z.-R., Shen, J.-D., Li, G.-Q., Wang, D.-Q., Jin-jun Li, J.-J. and Tian, Y. 2011. Effect of dietary fatty acids on serum parameters, fatty acid compositions, and liver histology in Shaoxing laying ducks. J. Zhejiang Univ. Sci. B. 12, 736–743.

Lobo, E.D., Hansen, R.J. and Balthasar, J.P. 2004 Antibody pharmacokinetics and pharmacodynamics. J. Pharmaceut. Sci. 93, 2645-2668.

Love, A.C., Lovern, M.B. and DuRant, S.E. 2017. Captivity influences immune responses, stress endocrinology, and organ size in house sparrows (*Passer domesticus*). Gen. Comp Endocrinol. 252, 18-26.

Lu, H.-P., Liu, P.-Y., Wang, Y.-b., Hsieh, J.-F., Ho, C.-H., Huang, S.-W., Lin, C.-Y., Hsieh, C.-h. and Yu, H.-T, 2017. Functional characteristics of the flying squirrel's cecal microbiota under a leaf-based diet, based on multiple metaomic profiling. Front. Microbiol. 8, 2622.

Luis-Lima, S., Mas-Sanmartin, C., Rodríguez-Rodríguez, A.E., Porrini, E., Ortiz, A., Gaspari, F., Diaz-Martin, L., Åsberg, A., Jenssen, T., Jiménez-Sosa, A., Martinez-Ros, P. and Gonzalez-Bulnes, A. 2020. A simplified iohexol-based method to measure renal function in sheep models of renal disease. Biology 9, 259.

Lv, Z., Xing, K., Li, G., Liu, D. and Guo, Y. 2018. Dietary genistein alleviates lipid metabolism disorder and inflammatory response in laying hens with fatty liver syndrome. Front. Physiol. 9,1493.

Lyons, J.E., Collazo, J.A. and Guglielmo, C.G. 2008. Plasma metabolites and migration physiology of semipalmated sandpipers: refueling performance at five latitudes. Oecologia 155, 417–427.

Maas, M., Keet, D. F. and Nielen, M. 2013. Hematologic and serum chemistry reference intervals for free-ranging lions (*Panthera leo*). Res. Vet. Sci. [95,](http://www.sciencedirect.com/science/journal/00345288/95/1%22%20%5Co%20%22Go%20to%20table%20of%20contents%20for%20this%20volume/issue)  266–268.

Maddox, D.A., Deen, W.M. and Brenner, B.M. 1974. Dynamics of glomerular ultrafiltration. VI. Studies in the primate. Kidney Int. 5, 271–278.

Madjdzadeh, S.M., Abbasnejad M., Takalloozadeh H.M. 2011. Haematology and some biochemical parameters of wild rodents in Pistachio Gardens of Kerman Province, southeast Iran. Chin. J. Appl. Environ. Biol., 17 907–909.

Mahmood, K.H., Stanford, J.L., Machin, S., Watts, M., Stuart, F.A. and Pritchard, D.G. 1988. The haematological values of European badgers (*Meles meles*) in health and in the course of tuberculosis infection. Epidemiol. Infect. 101, 231–237.

Maldonado, E.N., Casanave, E.B. and Aveldaño, M.I. 2002. Major plasma lipids and fatty acids in four HDL mammals. Comp. Biochem. Physiol. A 132, 297-303.

Malik, K., Awan, K. and Lone, K.P. 2012. Effect of feeding rapeseed meal on the nucleic acids concentrations in liver of Japanese quail. Afr. J. Microbiol. Res. 6, 3984-3988.

Malvin, R.L. and Rayner, M.R.1968. Renal function and blood chemistry in Cetacea. Am. J. Physiol. 214, 187–191.

Mankarious, S., Lee, M., Fischer, S., Pyun, K.H., Ochs, H.D., Oxelius, V.A. and Wedgwood, R.J. 1988. The half-lives of IgG subclasses and specific antibodies in patients with primary immunodeficiency who are receiving intravenously administered immunoglobulin. J. Lab. Clin. Med. 112, 634–640.

Markowska-Daniel, I., Pomorska-Mól, M. and Pejsak, Z. 2010. Dynamic changes of immunoglobulin concentrations in pig colostrum and serum around parturition. Pol. J. Vet. Sci.13, 21-27.

Marco, I. and Lavín, S. 1999. Effect of the method of capture on the haematology and blood chemistry of red deer (Cervus elaphus). Res Vet Sci. 66, 81–84.

Marco, I., Martinez, F., Pastor, J. and Lavin, S. 2000. Hematologic and serum chemistry values of the captive European wildcat. J. Wildl. Dis. 36, 445-449.

Marco, I., Cuenca, R., Pastor, J., Velarde, R., and Lavin, S. 2003. Hematology and serum chemistry values of the European brown hare. Vet. Clin. Pathol. 32, 195-198.

Marini, R.P., Otto, G., Erdman, S., Palley, L. and Fox, J.G. 2002. Biology and diseases of ferrets. Lab. Anim. Med.: 483–517.

Marler, R.J. 1975. Some hematogic and blood chemistry values in two herds of American bison in Kansas. J. Wildl.Dis. 11, 97-100.

Martino, P.E., Aráuz, S.M., Anselmino, F., Cisterna, C.C., Silvestrini, M.P., Corva, S. and Hozbor, F.A. 2012. Hematology and serum biochemistry of free-ranging nutria (*Myocastor coypus*). J. Zoo Wildl. Med. 43, 240-247.

Matsuzawa, T., Nomura, M. and Unno, T. 1993. Clinical Pathology Reference Ranges of Laboratory Animals. J. Vet. Med. Sci. 55, 351- 362.

Mattoso, C.R.S., Catenacci, L.S., Beier, S.L., Lopes, R.S. and Takahira, R.K. 2012. Hematologic, serum biochemistry and urinary values for captive Crab-eating Fox (*Cerdocyon thous*) in São Paulo state, Brazil. *Pesquisa Veterinária Brasileira*, *32*(6), 559-566.

May-Júnior, J.A., Songsasen, N., Azevedo, F.C., Santos, J.P., Paula, R.C., Rodrigues, F.H., Rodden, M.D., Wildt, D.E. and Morato, R.G. 2009. Hematology and blood chemistry parameters differ in free-ranging maned wolves (*Chrysocyon brachyurus*) living in the Serra da Canastra National Park versus adjacent farmlands. Brazil. J. Wildl. Dis. 45, 81-90.

McAlpine, D. F. 1985. Size and growth of heart, liver, and kidneys in North Atlantic fin (*Balaenoptera physalus*), sei (*B*. *borealis*), and sperm (*Physeter microcephalus*) whales. Can. J. Zool. 63, 1402-1409.

McClelland, K.L., Hume, I.D. and Soran, N. 1999. Responses of the digestive tract of the omnivorous northern brown bandicoot, *Isoodon macrourus* (Marsupialia: Peramelidae),

to plant- and insect-containing diets.

McDonald, S.E., Paul, S.R. and Bunch, T.D. 1981. Physiologic and hematologic values in Nelson desert bighorn sheep. J Wildl Dis. 17, 131-134.

Medway, W. and Kare, M.R. 1958. Blood and plasma volume, hematocrit, blood specific gravity and serum protein electrophoresis of the chicken. Poult. Sci. 624-631.

Melnychuk, V.L., Robinson, F.E., Renema, R., Hardin, R.T., Emmerson, D. and Bagley, L.G. 1997. Carcass traits and reproductive development at the onset of lay in two lines of female turkeys. Poult. Sci. 76:1197-1204.

Mendonça, M.A.C., Carvalho, C.R. and Clarindo, W.R. 2010. DNA content differences between male and female chicken (*Gallus gallus domesticus*) nuclei and z and w chromosomes resolved by image cytometry. J. Histochem. Cytochem. 58: 229–235.

Mendonça, M.A.C., Carvalho, C.R. and Clarindo, W.R. 2016. DNA amount of chicken chromosomes resolved by image cytometry. Caryologia, 69:3, 201-206.

Meucci, V., Sgorbini, M., Bonelli, F., Corazza, M., Lippi,I., Intorre, L. and Guidi, G. 2015 . Determination of glomerular filtration rate in adult horses and donkeys by single iv administration of iohexol. J. Equine Vet. Sci. 35, 36-40.

Michigoshi, Y., Yamagishi, N., Hiroshi, Show, S., Kato, M. and Furuhama, K. 2011. Using a single blood sample and inulin to estimate glomerular filtration rate in rabbits. J. Am. Assoc. Lab. Anim. Sci. 50, 702-707.

Millán, J., Gortázar, C. and Villafuerte, R. 2001. Marked differences in the splanchnometry of far,-bred and wild red-legged partridges (*Alectoris rufa* L.). Poult. Sci. 80, 972-975.

Miller, D.L., Leopold, B.D., Gray, M.J. and Woody, B.J. 1999. Blood parameters of clinically normal captive bobcats (*Felis rufus*). J Zoo Wildl Med. 30:242-247.

Milner, J.M., Stien, A., Justin Irvine, R., Albon, S.D., Langvatin, R. and Ropstad, E. 2003. Body condition in Svalbard reindeer and the use of blood parameters as indicators of condition and fitness. Can. J. Zool. 81, 1566–1578.

Mira, A. and da luz Mathias, M. 1994. Seasonal effects on the hematology and plasma proteins of two species of mice *Mus musculus domesticus* and *M. spretus* (*Rodentia: Muridae*) from Portugual. Hystrix 5, 63-72.

Mishra, A., Chatterjee, U.S. and Mandal, T.K. 2013. Induction of chronic renal failure in goats using cisplatin: a new animal model. Tox. Int. 20, 56-60.

Mitjans, M. and Ferrer, R. 2004. Morphometric study of the guinea pig small intestine during development. Misc. Res. Tech. 63, 206-214.

Moen, R., Rasmussen, J.M., Burdett, C.L. and Pelican, K.M. 2010. Hematology, serum chemistry, and body mass of free-ranging and captive Canada lynx in Minnesota. J. Wildl. Dis. 46, 13-22.

Mohri, M., Aslani, M. R. and Shahbazian, N. 2000. Haematology of Persian fallow deer (*Dama mesopotamica*). Comp Haematol Int. 10, 183-186.

Monte-Wicher, V., Wicher, K. and Arbesman, C.E. 1970. Comparative studies of monkey and human immunoglobulins. Immunochemistry 7, 839-844.

Morejohn, G.V., Ames, J.A. and Lewis, D.B. 1975. Post mortem studies of sea otters, Enhydra lutris L. in California. Marine Resources Technical Report 30. California Department of Fish and Game.

Morris, B. and Curtice, F.C. 1955. The protein and lipid composition of plasma of different animal species determined by electrophoresis and chemical analysis. J. Physiol. 40, 127 - 137

Morton, M. L. 1994. Hematocrits in montane sparrows in relation to reproductive schedule. Condor 96, 119-126.

Montes, G., Vásquez, A., Flores, E., Cattaneo, G., Acuña, M., and Cattan, P. 2004. Hematology, serum chemistry and physiological charactreristics of captive south pudu *Pudu* *pudu. Avances en Ciencias Veterinarias* **19**, 62-65.

[Moreau, B](http://www.ncbi.nlm.nih.gov/pubmed?term=Moreau%20B%5BAuthor%5D&cauthor=true&cauthor_uid=12885133)., [Vié, J.C](http://www.ncbi.nlm.nih.gov/pubmed?term=Vi%C3%A9%20JC%5BAuthor%5D&cauthor=true&cauthor_uid=12885133)., [Cotellon, P](http://www.ncbi.nlm.nih.gov/pubmed?term=Cotellon%20P%5BAuthor%5D&cauthor=true&cauthor_uid=12885133)., [De Thoisy, I](http://www.ncbi.nlm.nih.gov/pubmed?term=De%20Thoisy%20I%5BAuthor%5D&cauthor=true&cauthor_uid=12885133)., [Motard, A](http://www.ncbi.nlm.nih.gov/pubmed?term=Motard%20A%5BAuthor%5D&cauthor=true&cauthor_uid=12885133). and [Raccurt, C.P](http://www.ncbi.nlm.nih.gov/pubmed?term=Raccurt%20CP%5BAuthor%5D&cauthor=true&cauthor_uid=12885133). 2003. Hematological and serum biochemistry values in two free-ranging porcupines (Coendou prehensilis, Coendou melanurus) in French Guinea. [J. Zoo. Wildl. Med.](http://www.ncbi.nlm.nih.gov/pubmed/?term=HEMATOLOGIC+AND+SERUM+BIOCHEMISTRY+VALUES+IN+TWO+SPECIES+OF+FREE-RANGING+PORCUPINES+(COENDOU+PREHENSILIS%2C+COENDOU+MELANURUS)+IN+FRENCH+GUIANA) **34**, 159-62.

Moresco, A., Larsen, R. S., Sauther, M. L., Cuozzo, F. P., Youssouf Jacky, I. A. and Millette, J. B. 2012. Survival of a wild ring-tailed lemur (*Lemur catta*) with abdominal trauma in an anthropogenically disturbed habitat. Madagascar Conservation & Development 7, 1: 45–48. Supplementary Material. (doi:10.4314/mcd.v7i1.9)

Mossab, A., Lessire, M., Guillaumin, S., Kouba, M., Mourot, J., Peiniau, P. and Hermier, D. 2002. Effect of dietary fats on hepatic lipid metabolism in the growing turkey. Comp. Biochem. Physiol. B 132, 473–483.

Munro, H.N. and Gray, J.A.M. 1969. The nucleic acid content of skeletal muscle and liver in mammals of different body size. Comp. Biochem. Physiol. 28, 897-905.

Murphy, S.M. and Linhart, Y.B. 1999. Comparative morphology of the gastrointestinal tract in the feeding specialist *Sciurus aberti* and several generalist congeners. J. Mammal. 80, 1326-1330.

Mussart, N.B., Kozza, G.A., Solis, G., Coppo, J.A. 2009. Approach to some hematological variables of healthy captive “yaguarete” (*Panthera onca*) from Northeast Argentina. Rev. Vet., 20, 50-53.

Mustonen, A.M., Bowman, J., Sadowski, C., Nituch, L.A., Bruce, L., Halonen, T., Puukka, K., Rouvinen-Watt, K., Aho, J. and Nieminen, P. 2013. Physiological adaptations to prolonged fasting in the overwintering striped skunk (Mephitis mephitis). [Comp. Biochem. Physiol. 166A](http://europepmc.org/search?page=1&query=ISSN:%221095-6433%22), 555-563.

Needham, D., Cargill C., and Sheriff D. 1980. Haematology of the Australian sea lion, *Neophoca cinerea*. J Wildl Dis 16: 103–107.

Neill, A.R., Reichmann, K.G. and Conner, J.K. 1977. Biochemical, physiological and production indices related to fat metabolism in the laying fowl at various stages of physiological development. Br. Poult. Sci. 18, 315-324.

Němeček, T., Tůmová, E. and Chodová, D. 2019. Effect of sex on growth, biochemical and haematological parameters of blood, carcass value and meat quality in nutrias (*Myocastor coypus*). Czech Journal of Animal Science 64, 166–173.

Nimitsuntiwong, W., Homswat, S., Boonprakob, U., Kaewmokul, S., and Scmidt, A. 2000. Hematological and plasma biochemical values in captive eld’s-brow antlered deer (*Cervus eldi thamin*) in Thailand*.*  *J. Vet. Med. Sci.* 62, 93–95.

Norberg, S.E., Burkanov, V.N., Tuomi, P. and Andrews, R.D. 2011. Hematology of free-ranging, lactating northern fur seals, *Callorhinus ursinus*. J Wildl Dis. 47, 217-221.

Norman SA, Goertz CE, Burek KA, Quakenbush LT, Cornick LA, Romano TA, Spoon T, Miller W, Beckett LA, Hobbs RC. 2012. Seasonal hematology and serum chemistry of wild beluga whales (*Delphinapterus leucas*) in Bristol Bay, Alaska, USA. J Wildl Dis. 48, 21-32.

Oakeson, B. B. 1956. Liver and spleen weight cycles in non-migratory white-crowned sparrows. Condor 58, 45-50.

Ogunro, B.N., Otuh, P. I., Ogunsola, J.O., Kolawole, O.N., Jegede, H.O., Makinde, O., Ademakinwa, J. and Morenikeji O. 2019. Haemogram and serum biochemical values of four indigenous species of monkeys in South West Nigeria. Niger. J. Physiol. Sci. 34, 77-81.

Okeudo, N.J. Okoli, I.C. and Igwe, G.O.F. 2003. Hematological characteristics of ducks (*Cairina moschata*) of Southeastern Nigeria. Tropicultura 21, 61-65.

Okpe, C., Godwin, A., Nwabugwu C. and Adigwe, A. 2016. Evaluation of the morphological adaptations of the small intestine of the African pied crow (*Corvus albus*). J. Basic Appl. Zool. 75,54-60.

Old, J. M., Connelly, L., Francis, J., Branch, K., Fry, G., and Deane, E. M. 2005. Haematology and serum biochemistry of three Australian desert murids: the Plains rat (*Pseudomys australis*), the Spinifex hopping-mouse (*Notomys alexis*) and the Central rock-rat (*Zyzomys pedunculatus*). Comp. Clin. Path. 14, 130–137.

Old, J. M., Connelly, L., Francis, J. and Gogler, J. 2007. Haematology and serum biochemistry of the Carpentarian Rock-rat (*Zyzomys palatalis*). Comp. Clin. Path. 16, 249–252.

Olivier, T.R., Brand, T.S. and Gous, R.M. 2009. Growth and development of the reproductive organs of female breeding ostriches. S. Afr. J. Anim. Sci. 39, 260-262.

Oliveira, M.C.d., Silva, D.M.d. and Dias, D.B.M. 2013. Effect of feed restriction on organs and intestinal mucosa of growing rabbits. R. Bras. Zootec., Viçosa 42, 530-534.

Olsen, M., Myklebust, R., Kaino, T., Vibeke Elbrønd, V. and Svein Mathiesen, S. 2002. The gastrointestinal tract of Adélie penguins – morphology and function. Polar Biology 25, 641–649.

Ono, M., Akuzawa, H., Nambo, Y., Hirano, Y., Kimura, J., Takemoto, S., Nakamura, S., Yokota, H., Himeno, R., Higuchi, T., Ohtaki, T. and Tsumagari, S. 2015. Analysis of the equine ovarian structure during the first twelve months of life by three-dimensional internal structure microscopy. J. Vet. Med. Sci. 77, 1599–1603.

Opara, M. N., Ike, K. A. and Okoli I. C. 2006. Haematology and plasma biochemistry of the wild adult african grasscutter (*Thryonomis swinderianus*, Temminck). J. Am. Sci. 2, 17-22

Oxender, W.D., Colenbrander, B., van deWiel, D.F.M. and Wensing, C.J.G. 1979. Ovarian development in fetal and prepubertal pigs. Biol. Reprod. 21, 715-721.

Oyewale, J.O., Ogunsanmi, O.A. and Ozegbe, P.C. 1997. Haematology of the adult African white-bellied Pangolin (*Manis tricuspis*). Vet. Arch. 67, 261.

Oyewale, J.O., Olayemi, F.O. and Oke, O.A. 1998. Haematology of the wild adult African giant rat (*Cricetomys gambianus*, Waterhouse). Veterinarski arhiv 68, 91-99.

Palomares, F., Delibes, M., and Recio, F. 1992. Hematology and serum biochemistry of the Egyptian mongoose, *Herpestes ichneumon*. J. Wildl.Dis*.* 28, 659-661.

Patterson, R., Youngner, J.S., Weigle, W.O. and Dixon, F.J. 1962. The metabolism of serum proteins in the hen and chick and secretion of serum proteins by the ovary of the hen. J. Gen. Physiol. 45, 501-513.

Patterson, D.S.P., Allens, M., Berrett, S. and Sweasey, D. 1968. The chemical composition and capacity for protein synthesis of the liver of cattle suffering from clinical Johne’s disease. J. Med. Microbiol. 1, 137 - 143.

Paulini, I., Mehta, T. and A Hargis, A. 2006. Intestinal structural changes in African green monkeys after long term psyllium or cellulose feeding. J. Nutr. 117, 253-266.

Pedersen, R. J. and Pedersen, A. A.1975. Blood chemistry and hematology of elk. J. Wildl. Manag. 39, 617-620.

Peebles, E.D., Burnham, M.R., Walzem, R., Branton, S.L. and Gerard, P. 2004. Effects of fasting on serum lipids and lipoprotein profiles in the egg-laying hen (*Gallus domesticus*). Comp. Biochem. Physiol. A 138, 305-311.

Peppard, J.V. and Orlans, E. 1980. The biological half-lives of four rat immunoglobulin isotypes. Immunology 40, 683–686.

Pérez, J.M., González, F.J., Granados, J.E., Pérez, M.C., Fandos, P., Soriguer, R.C. and Serrano, E. 2003. [Hematologic and biochemical reference intervals for Spanish ibex.](http://www.ncbi.nlm.nih.gov/pubmed/12685085) J Wildl Dis. 39, 209-215.

Pérez, W., Lima, M., B**üker, M. and Clauss, M. 2017. Gross anatomy of the stomach** and intestine of an Antarctic minke whale (*Balaenoptera bonaerensis.* Mammalia 81, 111-113.

Peterson, A.J. 1955. The breeding cycle of the bank swallow. Wilson Bulletin 67, 236.

Pillay, P. and Manger, P.R. 2004. Testing thermogenesis as the basis for the evolution of cetacean sleep phenomenology. J. Sleep Res.13, 353–358.

Plön, S., Albrecht, K.H., Clif, G. and Froneman, P.W. 2012. Organ weights of three dolphin species (*Sousa chinensis*, *Tursiops aduncus* and *Delphinus capensis*) from South Africa: implications for ecological adaptation. J. Cetacean Res. Manag. 12, 265-276.

Presidente, P.J.A., Lumsden, J.H., Presnell, K.R., Rapley, W.A. and McGraw, B.M. 1973. Combination of etorphine and xylazine in captive white-tailed deer: II. Effects on hematologic serum biochemical and blood gas values. J. Wildl Dis 9, 342-348.

Pomorska-Mól, M., Krzysiak, M.K., Larska, M. and Włodarek, J. 2020. The first report of immunoglobulin G, M, and A concentrations in serum of European bison and their changes with age. J. Immunol. Res. 2020, Article ID 2614317.

Pospísil, J., Kase, F., Vahala, J. and Mouchová, I. 1984a. Basic haematological values in antelopes--II. The Hippotraginae and the Tragelaphinae. Comp. Biochem. Physiol. A 78, 799-807.

Pospísil, J., Kase, F., Vahala, J. and Mouchová, I. 1984b. Basic haematological values in antelopes--III. The Reduncinae and the Antelopinae. Comp Biochem Physiol A 78, 809-813.

Pospísil, J., Vahala, J., Kase, F. and Fraisova, L. 1985. Haematological values in the peripheral blood of zebras kept in the East-Bohemian Zoological Gardens at Dvur Kralove. Acta Vet. Brno 54, 129-140.

Pospísil, J., Kase, F. and Vahala, J. 1987a. Basic haematological values in carnivores--I. The Canidae, the Hyaenidae and the Ursidae. Comp. Biochem. Physiol. A. 86, 649-652.

Pospísil, J., Kase, F. and Váhala, J. 1987b. Basic haematological values in carnivores--II. The Felidae.

Comp. Biochem. Physiol. A 87, 387-391.

Presidente, P.J.A., Lumsden, J.H., K. Presnell, R., Rapley, W. and McCraw, B.M. 1973. Combination of etorphine and zylazine in captive white-tailed deer. II. Effects on hematologic, serum biochemical and blood gas values. J. Wildl. Dis. 9, 342 - 348.

Prihirunkit, K., Salakij, C., Apibal, S. and Narkkon, N.-A. 2007. Hematology, cytochemistry and ultrastructure of blood cells in fishing cat (*Felis viverrina*). J. Vet. Sci. 8, 163–168.

Priddel. D. and Wheeler, R. 1998. Hematology and blood chemistry of a Bryde’s whale, *Balaenoptera edeni*, entrapped in the Manning river, New South Wales, Australia. Mar.Mam. Sci. 14, 72-81.

Putaala, A. and Hissa, R. 1995. Effects of hand-rearing on physiology and anatomy in the grey partridge. Wildl. Biol. 1, 27-31.

Quist, C.F., Bounous, D.I., Kilburn, J.V., Nettles, V.F. and Wyatt R.D. 2000. The effect of dietary aflatoxin on wild turkey poults. J. Wildl. Dis. 36, 436-444.

Ran, M., Hu, B., Cheng, L., Hu, S., Liu, H., Li, L., Hu, J. and Wang, J. 2020. Paternal weight of ducks may have an influence on offspring’ small intestinal function and cecal microorganisms BMC Microbiol. 20, 145.

Ratnasooriya, W.D., Udagama-Randeniya, P.V., Yapa, W.B. and Dharmasira, M.G. 2005. Haematogical parameters of three species of wild caught microchiropteran bats, *Miniopterus schreibersii, Taphozous melanopogon* and *Hipposiderus lankadiva* in Sri Lanka. J. Sci. Univ. Kelaniya 2, 27-40.

[Reiss A](http://www.ncbi.nlm.nih.gov/pubmed?term=Reiss%20A%5BAuthor%5D&cauthor=true&cauthor_uid=18263822), [Portas T](http://www.ncbi.nlm.nih.gov/pubmed?term=Portas%20T%5BAuthor%5D&cauthor=true&cauthor_uid=18263822), [Horsup A](http://www.ncbi.nlm.nih.gov/pubmed?term=Horsup%20A%5BAuthor%5D&cauthor=true&cauthor_uid=18263822). 2008. Hematologic and serum biochemical reference values for free-ranging northern hairy-nosed wombats. [J Wildl Dis.](http://www.ncbi.nlm.nih.gov/pubmed/?term=HEMATOLOGIC+AND+SERUM+BIOCHEMICAL+REFERENCE+VALUES+FOR+FREE-RANGING+NORTHERN+HAIRY-NOSED+WOMBATS) 44, 65-70.

Rice[^,^](http://www.bioone.org/doi/abs/10.3955/0029-344X-81.3.206?journalCode=nwsc#aff1) C. G. and Hall. B. 2007. Hematologic and biochemical reference intervals for mountain goats (*Oreamnos americanus*): effects of capture conditions. Northwest Science **81**, 206-214. 2007

Richmond, J.P., Burns, J.M., Rea, L.D. and Kendall L Mashburn, K.L. 2005. Postnatal ontogeny of erythropoietin and hematology in free-ranging Steller sea lions (*Eumetopias jubatus*). Gen. Comp. Endocrinol. 141, 240-247.

Ricklefs, R.E. 1974. The chemical composition of the ovary, oviduct and follicles of the starling. Auk 93, 184 - 187.

Rivet, D.R., Nelson, O.L., Vella, C.A., Jansen, H.T. and Robbins, C.T. 2017. Systemic effects of a high saturated fat diet in grizzly bears (*Ursus arctos horribilis*). Can. J. Zool. 95, 797-807.

Robertson, J.A., Guzman D.S.-M., Graham, J.L., Stanhope, K.L., Douglas, J.M., Havel, P.J., Beaufrère, H., Knych, H., Tully, T.N. and Paul-Murphy, J.R. 2020. Atorvastatin on plasma lipid and biochemistry profiles in hypercholesterolemic Hispaniolan amazon parrots (*Amazona ventralis*). J. Avian Med. Surg. 34, 32-40.

Robel, G.L., Lochmiller, R.L., McMurry, S.T. and Qualls, C.W. Jr. 1996. Environmental, age, and sex effects on cotton rat (*Sigmodon hispidus)* hematology. J. Wildl. Dis. 32, 390-4.

Rosas, F.C.W; Neto, J.A.A. and Mattos, G. 2008. Anesthesiology, hematology and serum chemistry of the giant otter, *Pteronurabrasiliensis* (carnivora, mustelidae. Arq. Ciênc. Vet. Zool. Unipar, Umuarama 11, 81-85.

Rostal, M.K., Evans, A.L., Solberg, E.J. and Arnemo, J.M. 2012. Hematology and serum chemistry reference ranges of free-ranging moose (*Alces alces*) in Norway. [J. Wildl. Dis.](http://www.ncbi.nlm.nih.gov/pubmed/?term=HEMATOLOGY+AND+SERUM+CHEMISTRY+REFERENCE+RANGES+OF+FREE-RANGING+MOOSE+(ALCES+ALCES)+IN+NORWAY%22%20%5Co%20%22Journal%20of%20wildlife%20diseases.) 48, 548-559.

[Rothstein R](http://www.ncbi.nlm.nih.gov/pubmed?term=Rothstein%20R%5BAuthor%5D&cauthor=true&cauthor_uid=4336994), [Hunsaker D 2nd](http://www.ncbi.nlm.nih.gov/pubmed?term=Hunsaker%20D%202nd%5BAuthor%5D&cauthor=true&cauthor_uid=4336994). 1972. Baseline hematology and blood chemistry of the South American woolly opossum, *Caluromys derbianus*. [*Lab. Anim. Sci*.](http://www.ncbi.nlm.nih.gov/pubmed/4336994) 22:227-232.

Rovirosa-Hernández, M.d.J., García- Orduña, F., Morales-Mávil, J.e. Hernández- Salazar, L.T., Hermida-Lagunes, J., Lagunes-Merino, O. and Fuentes-Anaya. T. 2012. Hematological and blood chemistry values in a semi-free population of white-nosed coatis (*Nasua narica*) in La venta Tabasco, Mexico. Acta Zoológica Mexana 28, 391- 400. 2120.

Sajjad, S., Farooq, U., Malik, H., Anwar,M. and Ahmad, I. 2012. Comparative hematological variables of Bengal tigers (Panthera tigris tigris) kept in Lahore Zoo and Lahore Wildlife Park, Pakistan. Turk. J. Vet. Anim. Sci. 36, 346-351.

Salakij, C., Salakij, J. Rattanakunuprakarn, J., Tengchaisri, N., Tunwattana, W. and Apibal, S. 2000. Morphology and Cytochemistry of Blood Cells from Asian Wild Dog (*Cuon alpinus).*

<http://www.thaiscience.info/Article%20for%20ThaiScience/Article/5/Ts-5%20morphology%20and%20cytochemistry%20of%20blood%20cells%20from%20asian%20wild%20dog%20(cuon%20alpinus).pdf> accessed 12.17.13

Salakij, C., Salakij, J., Narkkong, N.-A., Tongthainun, D., Prihirunkit, K. and Itarat, S. 2007. Hematology, cytochemistry and ultrastructure of blood cells in common palm civet (*Paradoxurus hermaphroditus*). Kasetsart J. (Nat. Sci.) 41, 705 – 716.

Salakij, C., Prihirunkit, K., Narkkong, N.A., Apibal, S. and Tongthainun, D. 2008a. Hematology, cytochemistry and ultrastructure of blood cells in clouded leopard *(Neofelis nebulosa*). J. Anim. Vet. Advances 7, 847-853.

Salakij, C., Salakij, J., Narkkong, N.A., Sirinarumitr, T. and Pattanarangsan, R. 2008b. Hematologic, cytochemical, ultrastructural, and molecular findings of Hepatozoon-infected flat-headed cats (*Prionailurus planiceps*). Vet. Clin. Pathol. 37, 31-41.

Salakij, C., Prihirunkit, K., Salakij, J., Narkkong, N.-A. and Thongthainun, D. 2010. Characterisation of blood cells in jungle cat, *Felis chaus* (Carnivora, Felidae). Comp. Clin. Pathol. 20, 319-326.

Salvante, K.G., Lin, G., Walzem, R.L. and Williams, T.D. 2007. Characterization of very-low density lipoprotein particle diameter dynamics in relation to egg production in a passerine bird. J. Exp. Biol. 210, 1064-1074.

Sanches, T. C., Miranda, F. R., Oliveira, A. S. and Matushima, E. R. 2013. Hematology values of captive giant anteaters (*Myrmecophaga tridactyla*) and collared anteaters (*Tamandua tetradactyla*). Pesquisa Veterinária Brasileira 33, 557-560.

Sasaki, Y., Iwama, R., Sato, T., Heishima, K., Shimamura, S., Ichijo, T., Satoh, H. and Furuhama, K. 2014. Estimation of glomerular filtration rate in conscious mice using a simplified equation. Physiol. Rep. 2, e12135.

Schaal, T.P., Arango, J., Wolc, A., Brady, J.V., Fulton, J.E., Rubinoff, I., Ehr, I.J., Persia, M.E. and O’Sullivan, N.P. 2016. Commercial Hy-Line W-36 pullet and laying hen venous blood gas and chemistry profiles utilizing the portable i-STAT®1 analyzer. Poult Sci. 95, 466–471.

Schwacke, L.H., Hall, A.J., Townsend, F. I., Wells, R.S., Hansen, L.J., Hohn, A.A., Bossart, G.D., Fair, P.A., Rowles, T.K. 2009. Hematology and clinical blood chemistry reference intervals for free-ranging common bottlenose dolphins (*Tursiops truncatus*) and variation related to geographic sampling site. Am. J. Vet. Res. 70, 973-985.

Schinnerl, M., Aydinonat, D., Schwarzenberger, F. and Voigt, C. C. 2011. Hematological survey of common neotropical bat species from Costa Rica. J. Zoo Wildl. Med. 42, 382-391.

Schoeder, M. T.1987. Blood chemistry, hematology, and condition evaluation of black bears in Northern California. Int. Conf. Bear Res. and Manage. 7, 333-349

Seal, U.S., Siniff, D.B., Tester, J.R. and Williams, T.D. 1985. Chemical immobilization and blood analysis of feral horses (*Equus caballus*). J. Wildl. Dis. 21, 411-416.

Sedinger, J.S. 1986. Growth and development of Canada goose goslings. Condor, 88, 169-180.

Seeber, P.A., Morrison, T., Ortega, A., East, M.L., Greenwood, A.D. and Czirják, G.A. 2020. Immune differences in captive and free-ranging zebras (*Equus zebra* and *E. quagga*). Mammalian Biology 100, 155–164.

Seyyedin, S. and Nazem, M.N. 2017. Histomorphometric study of the effect of methionine on small intestine parameters in rat: an applied histologic study. Folio Morphol. 76, 620-629.

Shanmugam, A.A., Kumar, J.K., Selvaraj, I. and Selvaraj, V. 2008. Hematology of sloth bears (*Melursus ursinus ursinus*) from two locations in India. J. Wildl. Dis. 44, 509-518.

Shave, H. and Howard, V. 1976. A hematologic survey of water fowl. J. Wildl.Dis. 12, 195-201.

Sheridan, J. A., Beissinger, S. R. and Hughes, C. R. 2004. Weak association between measures of health and reproductive success in green-rumped parrotlets (*Forpus passerinus*) in Venezuela. Auk 121, 717-725.

Shideler, S.E., Stoops, M.A., Gee, N.A. and Tell, L.A. 2002. Hematologic values for tule elk (*Cervus elaphus nannodes*). J. Wildl. Dis. 38, 589-597.

Shirai, K. and Sakai, T. 1998. Haematological findings of captive dolphins and whales. Aust. Vet. J. 75, 512-514.

Short, K. and Derrickson, E.M. 2020. Compensatory changes in villus morphology of lactating *Mus musculus* in response to insufficient dietary protein. J. Exp. Biol. 223, jeb210823.

Silva, I. D. and Kuruwita, V.Y. 1993. Hematology, Plasma, and Serum Biochemistry Values in Free-Ranging Elephants (*Elephas maximus ceylonicus*) in Sri Lanka. J. Zoo Wildl. Med*.*24, 434-439.

Silverin, B. 1980. Seasonal development of reproductive organs in the female pied flycatcher *Ficedula hypoleuc*a. J. Zool. (Lond.) 190, 241 - 257.

Singh, S., Singh, C., Kumar, A., Sinha, K.K., Mishra, P.C. 1999. Hematology of tigers (*Panthera tigris tigris*), leopards (*Panthera* *pardus*) and clouded leopards (*Neofelis nebulosa*) in captivity. Zoos’ Print 14, 7-8.

St. Aubin, D.J., Forney, K.A., Chivers, S.J., Scott, M.D., Daniel, K. and Romano, T.A. 2010. Hematological, serum, and plasma chemical constituents in pantropical spotted dolphins (*Stenella attenuata*) following chase, encirclement, and tagging. Marine Mammalogy 29, 14-35.

Stewardson, C.L., Hemsley, S., Meyer, M.A., Canfield, P.J. and Maindonald, J.H. 1999. Gross and microscopic visceral anatomy of the male Cape fur seal, *Arctocephalus pusillus pusillus* (Pinnipedia: *Otariidae*), with reference to organ size and growth. J. Anat. 195, 235-255.

Sturkie, P.D. 1986. Body fluids: blood. In: Avian Physiology Ed: P.D. Sturkie, pp.102-129. Springer, New York.

Suttie, J.M. and Fennessy, P.F. 1992. Organ weights and weight relationships in Takahe and Pukeko. Notornis 39, 47-53.

Sun, J., Zhang, X., Cao, Y., Zhao, Q., Bao, E. and Lv, Y. 2016. Ovarian toxicity in female rats after oral administration of melamine or melamine and cyanuric acid. PLoS One. 11: e0149063.

[Superina, M](http://www.ncbi.nlm.nih.gov/pubmed?term=Superina%20M%5BAuthor%5D&cauthor=true&cauthor_uid=18957646)., [Mera, Y. and Sierra, R.L](http://www.ncbi.nlm.nih.gov/pubmed?term=Mera%20Y%20Sierra%20RL%5BAuthor%5D&cauthor=true&cauthor_uid=18957646). 2008. Hematology and serum chemistry values in captive and wild pichis, *Zaedyus pichiy* (Mammalia, Dasypodidae). [J. Wildl. Dis.](http://www.ncbi.nlm.nih.gov/pubmed/18957646) 44, 902-10.

Szabó, M.P.J., Matushima, E.R., de Castro, M.B., Santana, D.A., de Paula, C.D. and Duarte, J.M.B. 2005. Hematology of free-living marsh deer (*Blastocerus dichotomus*). J. Zoo. Wildl. Med. 36, 463-469.

Takahashi, K. and Yamasaki, F. 1972. Digestive tract of Ganges dolphin, *Platanista gangetica*: Small and large intestine. Okajimas Fol. Anat.jap. 48, 427-452.

Thomas, N.E. and Swanson, D.L. 2013. Plasma metabolites and creatine kinase levels of

shorebirds during fall migration in the Prairie Pothole region. Auk 130, 580-590.

Tocidlowski, M.E., Spelman, L.H., Sumner, P.W., and Stoskopf, M.K. 2000. Hematology and serum biochemistry parameters of North American river otters (*Lontra Canadensis).* J. Zoo Wildl. Med. 31, 484–490.

Tome, M.W. 1984. Changes in the nutrient reserves ands organ size of female ruddy duckas breeding in Manitoba. Auk 101, 830 – 837.

Tomkins, N.W. and Jonsson, N.N. 2005. Haematological values of young male rusa deer (*Cervus timorensis*). Aust. Vet. J. 83, 496-498.

Tootian, Z., Sadeghinezhad, J., Sheibani, M.T., Fazelipour, S., De Sordi, N. and Roberto Chiocchetti, R. 2013. Histological and mucin histochemical study of the small intestine of the Persian squirrel (*Sciurus anomalus*). Anat. Sci. Int. 88, 38–45.

Travis, E.K. and Eby, C. 2006. Clotting profiles and selected hematology of captive Speke's gazelles (*Gazella spekei*). [J. Zoo Wildl. Med.](http://www.ncbi.nlm.nih.gov/pubmed/17312817%22%20%5Co%20%22Journal%20of%20zoo%20and%20wildlife%20medicine%20:%20official%20publication%20of%20the%20American%20Association%20of%20Zoo%20Veterinarians.) 37, 64-67.

Turner, J.P., Clark, L.S., Haubold, E.M., Worthy, G.A.J., and Cowan, D.F. 2006. Organ weights and growth profiles in bottlenose dolphins (*Tursiops truncatus*) from the Northwestern Gulf of Mexico. Aquatic Mammals *32*, 46-57.

Vaughan, R.J., Warren, K.S., Mills, J.S., Palmer, C., Fenwick, S., Monaghan, C.L. and Friend, A.J. 2009. Hematological and serum biochemical reference values and cohort analysis in the Gilbert's potoroo (*Potorous gilbertii*). J. Zoo Wildl. Med. 40, 276-288.

Venn-Watson, S., Smith, C.R., Dold, C. and Ridgway, S.H. 2007. Use of a serum‐based glomerular filtration rate prediction equation to assess renal function by age, sex, fasting, and health status in bottlenose dolphins (*Tursiops truncatus*). Marine Mammal Science 24, 71 - 80

Vergneau-Grosset, C., Polley, T., Carrade, D., Holt, Vernau, W. and Paul-Murphy, J. 2016. Hematologic, plasma biochemical, and lipid panel reference intervals in orange-winged Amazon parrots (*Amazona amazonica*). J. Avian Med. Surg. 30, 335-344.

Verma, S., Sehgal, A.K., Chakravarti, R.N. and Chhuttan, P.N. 1968. Intestinal villi in the dog and effect of *Ankostoma caninum* infestation. J. Pathol. Bacteriol. 95, 568-571.

Vogel, I., Vie, J. C., De Thoisy, B. and Moreau, B. 1999. Hematological and serum chemistry profiles of free-ranging southern two-toed sloths in French Guiana. J. Wildl. Dis. 35, 531–535.

Von Hendy-Willson, V.E. and Pressler, B.M. 2011. An overview of glomerular filtration rate testing in dogs and cats. Vet J. 188, 156–165.

Wagner, E.C., Prevolsek, JS., Wynne-Edwards, K.E. and Williams, T.D. 2008. Hematological changes associated with egg production: estrogen dependence and repeatability. J. Exp. Biol. 211, 400-408.

Wall, C.W. and Anthony, N.B.1995. Inheritance of carcass variables when giant jungle fowl and broilers achieve a common physiological body weight. Poult. Sci. 74, 231-236.

Wallace, C., and Oppenheim, Y. C. 1996. Hematology and serum chemistry profiles of captive Hoffmann's Two-toed sloths (*Choloepus hoffmanni)*. J. Zoo. Wildl. Med. 27, 339-345.

Weaver, L.T. and Carrick, B.M. 1989. Changes in upper intestinal epithelial morphology and kinetics in the growing guinea pig. Pediatr. Res. 26, 31-33.

Weber Rosas, F.C. and Monteiro-Filho, E.L.A. 2002. Reproduction of the estuarine dolphin (*Sotalia guianensis*) on the coast of Parana ́, Southern Brazil. J. Mammol. 8:507–515.

Weber, D. K., Danielson, K., Wright, S. and Foley, J. E. 2002. Hematology and serum biochemistry values of dusky-footed wood rat (*Neotoma fuscipes*). J. Wildl Dis 38, 576-582.

White, D.H., Finley, M.T. and Ferrell, J.F. 1978. Histopathologic effects of dietary cadmium on kidneys and testes of mallard ducks. J. Toxicol. Environ. Health. 4: 551-558.

Wiedmeyer, C.E., Crossland, J.P., Veres, M., Dewey, M.J., Felder, M.R., Barlow, S.C., Vrana, P.B. and Gabor Szalai, G. 2014. Hematologic and serum biochemical values of 4 species of *Peromyscus* mice and their hybrids. J. Am. Assoc. Lab. Anim. Sci. 53, 336–343.

Wiersma, P., Nowak, B. and Williams, J. B. 2012. Small organ size contributes to the slow pace of life in tropical birds. J. Exp. Biol. 215, 1662-1669.

Wiesel, I., Zimmerman, D.M. and Suedmeyer, W.K. 2018. Select serologic screening of brown hyenas (*Parahyaena brunnea*) from the Namib desert, Namibia. J. Zoo Wildl. Med. 49, 931-942.

Weiss, D.J., Wustenberg, W., Bucci, T.J. and Perman, V. 1994. Hematologic and serum chemistry reference values for adult brown mink. J. Wildl. Dis. 30, 599-602.

Wiger, R., 1977. Hematology of the Norwegian lemming (*Lemmus lemmus(L.)*. Acta Zool. 58, 143–150.

Wilkanowska, A., Kokoszyński, D. and Cieślińska, J. 2013. Body conformation and morphometry of some internal organs of Pharaoh quail of different ages. J. Cent. Eur. Agric. 14, 358-368.

Williams, T. D. and Pulley L.T. 1983. Hematology and blood chemistry in the sea otter (*Enhydra lutris*). [J Wildl Dis.](http://www.ncbi.nlm.nih.gov/pubmed/18436689) 19, 44-47.

Woolf, A. and Kradel, D.C. 1970. Hemnatological values of captive rocket mountain bighorns. J. Wildl. Dis. 6, 67 - 68.

Wolff, M. J., Bratthauer, A., Fischer, D., Montali, R. J., Banish, L. D. and Bush, M. 1990. Hematologic and serum chemistry values for the Red Panda (*Ailurus fulgens*): Variation with sex, age, health status, and restraint. J. Zoo Wildl. Med. 21, 326-333.

Wołk, E. 1985. Hematology of a hibernating rodent – the Northern Birch Mouse. Acta Theriologica 30, 337-348.

Yamanaka, Y. 1989. Hematological study in sika deer (*Cervus nippon yesoensis*). Jpn. J. Vet. Res. 37 http://eprints.lib.hokudai.ac.jp/dspace/bitstream/2115/3182/1/KJ00002377285.pdf

Yamasaki, F. and Kito, K. 1984. A morphological note on the intestine of the boutu with emphasis on its length and ileo-colic transition compared with other platanistids. Sci. Rep. Whales Res. Inst. 35, 165 – 172.

Yang, H.M., Wang, W., Wang, Z.Y., Wang, J., Cao, Y.J. and Chen, Y.H. 2013. **Comparative study of intestinal length, weight and digestibility on different body weight chickens**. Afr. J. Biotechnol. 12, 5087-5100.

Yaralioğlu, S., Şahin, T., Şahin, N. and Yűreki, U.F. 2004. Investigation of some hematologic and biochemical parameters in the serum of gazelles (*Gazella subgutturosa*) in Ceylanpinar, Şanliurfa, Turkey. Turk*.*  J. Vet. Anim. Sci. 28, 369-372.

Yin, W., Carballo-Jane, E., McLaren, D.G., Mendoza, V.H., Gagen, K., Geoghagen, N.S.,McNamara, L.A., Gorski, J.N., Eiermann, G.J., Petrov, A., Wolff, M., Tong, X., Wilsie, L.C., Akiyama, T.E., Chen, J., Thankappan, A., Xue, J., Ping, X., Andrews, G., Wickham, L.A., Gai, C.L.,Trinh, T., Kulick, A.A., Donnelly, M.J., Voronin, G.O., Rosa, R., Cumiskey, A.-M., Bekkari, K., Mitnaul, L.J., Puig, O., Chen, F., Raubertas, R., Wong, P.H., Hansen, B.C., Koblan, K.S., Roddy, T.P., Hubbard, B.K. and Strack, A.M. 2012. Plasma lipid profiling across species for the identification of optimal animal models of human dyslipidemia. J. Lipid Res. 53, 51–65.

Youatt, W.G., Fay, L.D., Howe, D.L. and Harte, H.D. 1961. Hematologic data on some small mammals. Blood 18, 758-763.

Yochem, P.K., Stewart, B.S., Mazet, J.A. and Boyce, W.M. 2008. Hematologic and serum biochemical profile of the northern elephant seal (M*irounga angustirostri*s): variation with age, sex, and season. J. Wildl. Dis. 44, 911-921.

Zhan, Y.M., Yasuda, J. and Too, K. 1991. Reference data on the anatomy and serum biochemistry of the silver fox. [Jpn. J. Vet. Res.](http://www.ncbi.nlm.nih.gov/pubmed/1956149) 39, 39-50.

Zhao, S., Zhanga, C., Wang, J., Liu, G., Bu, D., Cheng, J. and Zhou, L. 2010. Variations of immunoglobulins in colostrum and immune milk as affected by antigen releasing devices. Asian-Aust. J. Anim. Sci. 23, 1184 – 1189.

Zitnan, R., Voigt, J., Kuhla, S., Wegner, J., Chudy, A., Schoenhusen, U., Brna, M., Zupcanova, M. and Hagemeister, H. 2008. Morphology of small intestinal mucosa and intestinal weight change with metabolic type of cattle. Veterinarni Medicina 53, 525–532
